# Photoinduced nucleophilic substitution of iodocubanes with arylthiolate and diphenylphosphanide ions. Experimental and computational approaches†

**DOI:** 10.1039/c8ra06275g

**Published:** 2018-11-23

**Authors:** Liliana B. Jimenez, Marcelo Puiatti, Diego M. Andrada, Federico Brigante, Karina F. Crespo Andrada, Roberto A. Rossi, Ronny Priefer, Adriana B. Pierini

**Affiliations:** INFIQC, Departamento de Química Orgánica, Facultad de Ciencias Químicas, Universidad Nacional de Córdoba, Ciudad Universitaria X5000HUA Córdoba Argentina ljimenez@fcq.unc.edu.ar; Krupp-Professur für Allgemeine und Anorganische Chemie, Universität des Saarlandes 66123 Saarbrücken Germany; College of Pharmacy, Western New England University Springfield Massachusetts 01119 USA

## Abstract

A new synthetic route to modify the cubane nucleus is reported here. Methyl-4-iodocubane-1-carboxylate (1) and 1,4-diiodocubane (2) were employed as reagents to react with arylthiolate and diphenylphosphanide ions under irradiation in liquid ammonia and dimethylsulphoxide. The reactions proceed to afford thioaryl- and diphenylphosphoryl- cubane derivatives in moderate to good yields. It is also found that the monosubstituted product with retention of the second iodine is an intermediate compound. Mechanistic aspects are supported by DFT calculations.

## Abstract 2

Photoinduced substitution of the cubane nucleus is reported here.

## Graphical abstract

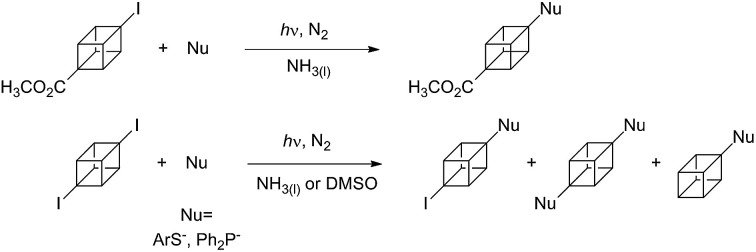

## Introduction

**Scheme 1:**
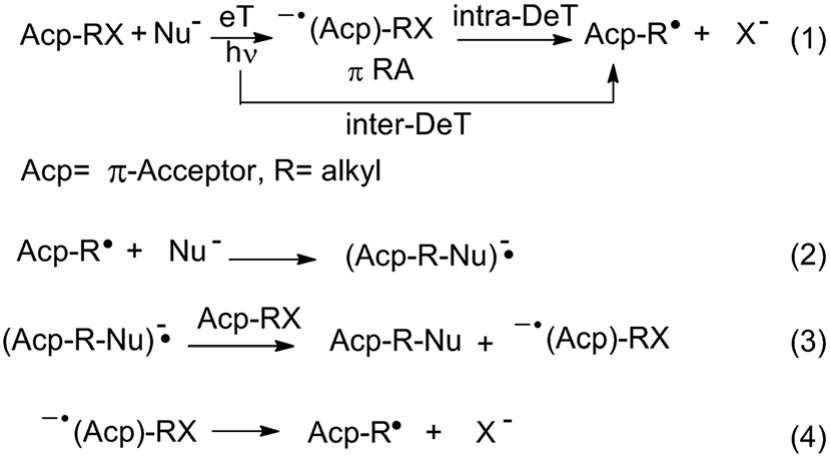
Mechanism of the unimolecular radical nucleophilic substitution or S_RN_1 process.

Within the different nucleophilic substitution mechanisms known, the unimolecular radical nucleophilic substitution or S_RN_1 process^1^ is an interesting alternative to the classical S_N_2 and S_N_1 mechanisms, with a continuously growing synthetic scope.^1,2^ The S_RN_1 reaction is a cyclic process mediated by electron transfer (eT) steps. Initiation can be spontaneous, thermal, induced by inorganic salts, electrochemically, or in most cases, photostimulated.^1^ When light is used, an electron can be transferred from the excited state of the nucleophile to the substrate to initiate the cycle. This event can be dissociative, which means that the carbon–halogen (C–X in Scheme 1)§§X refers to a leaving group. Most common leaving groups used in S_RN_1 are: I^−^, Br^−^, Cl^−^, (EtO)_2_P(O)O, RS (R = Ar, alkyl), ArSO, ArSO_2_, PhSe, Ph_2_S^+^, RSN_2_ (R = *t*-Bu, Ph), N_2_BF_4_, R_3_N^+^, N^2+^, N_3_, NO_2_, and XHg. bond breaks as the electron is being transferred (intermolecular dissociative eT (inter-DeT)), or can follow a two-step pathway with the formation of radical anion intermediates (eqn (1)). This latter pathway occurs preferentially when the molecules have a π-system that acts as an acceptor (Acp, Scheme 1). In the second step the intermediates, through an intramolecular dissociative eT (intra-DeT) mostly from the π system to the σ* C–X bond, fragment into the radical (eqn (1)) that enter the propagation cycle (eqn (2)–(4)) plus the halide anion, as is shown in Scheme 1.

This family of reactions represents an attractive alternative to conventional reactions since they are metal-free reactions leading to high yields under mild conditions. The latter feature provides suitable conditions for the inclusion of many compatible substituents (such as alkyl, OR, SAr, CF_3_, NHBoc, NHCOR, SO_2_R, among others) in substrate structure.^1^ Additionally, substrates with strain or steric factors which have been shown to react sluggishly or not at all by polar mechanisms, are excellent substrates towards nucleophilic substitutions involving eT steps.^1^ Examples recounted are those such as neopentyl, bicyclic, and polycycloalkyl bridgehead halides.^1^

Different heteroatom-centered anions have been used in S_RN_1 reactions as nucleophiles to generate a new C-heteroatom bond.^1,2^ In particular, it has been demonstrated that benzenethiolate and *p*-substituted benzenethiolate ions react with 1-iodoadamantane (1-IAd) under photochemical induction resulting in excellent substitution yields.^3^ Indeed, it has been also reported that the coupling of 1-XAd (X = halogen) is possible with Ph_2_P^−^, Ph_2_As^−^, PhSe^−^, PhTe^−^, Se^2−^ and Te^2−^ ions. Furthermore, it has been reported that sterically hindered substrates with two leaving groups, such as 1,4-dihaloadamantanes and 4-halo-1-iodobicyclo[2.2.2]octanes, led to disubstituted and/or monosubstituted products in very good yields depending on the nature of the second halogen and the anion used under the S_RN_1 conditions.^4^ Nucleophiles as benzenethiolates and Ph_2_P^−^ showed high substitution yields over carbanions on hindered substrates.^1^

In order to broaden the scope of the S_RN_1 reaction, we decided to explore its potential in the highly strained systems like cubanes. More than 50 years have passed since the first synthesis of the cubane carbon skeleton was reported.^5^ At least ten synthetic steps are needed to obtain dimethyl cubane-1,4-dicarboxylate and cubane.^6^ Derivatives of this regular polyhedron have attracted attention due to their unexpected chemical stability^7^ which resulted in applications in different areas of the chemistry from medicine to nanostructure design.^8–10^ Several modifications have been applied to the cubane scaffold; halogenations, photochemical solvolysis, metal-catalyzed substitutions, cross coupling substitutions based on single electron transfer using Ni and Fe as catalysts^11^ and halogen–metal exchange are some examples of landmark studies.^6,12^ Moreover, the formation of reactive intermediates such as cubyl radicals,^7*a*,13,14^ detected also by electron paramagnetic resonance (EPR),^13*a*^ cations^15^ and anions^16^ was confirmed through different chemical pathways.

In this context, we have been encouraged to make a contribution to the scope of the cubane chemistry by exploring the possibility of substituting the halogens within halocubane derivatives by means of S_RN_1. Clearly, the cubane skeleton does not favor both the back side attack on the traditional S_N_2 mechanism and the S_N_1 mechanism because of the considerable energy necessary to form the highly strained cubyl cation^15^ intermediate. Thus, the rigid strained structure of these compounds makes them interesting substrates to react by eT which might involve radical species with the cubyl skeleton.

The stability of both methyl-4-iodocubane-1-carboxylate (1) and 1,4-diiodocubane (2) has been studied under thermolytic conditions.^17^ They have not experienced any cage/rearrangement or cage opening/fragmentation reactions as is well-known to occur for iodinated cubane analogues.^18^ Therefore, each one was separately exposed to reactions with S- and P-centered anions, under eT conditions. Notably, the sulfur^7*c*,19^ or phosphine^7*c*^ cubane derivatives are not a recurrent motif in the current literature. Herein, we present a thorough experimental and computational study on the transformation of cubane scaffolds *via* S_RN_1 reaction with several sulfur and phosphorous nucleophiles.

## Results and discussion

### Experimental results

The substrates for S_RN_1 reactions, methyl-4-iodocubane-1-carboxylate (1) and 1,4-diiodocubane (2), were synthesized following previously published procedures.^17^ Both substrates are photostable since 95% and 97% of 1 and 2, respectively, were recovered after 2 hours of irradiation in absence of a nucleophile.

**Scheme 2:**
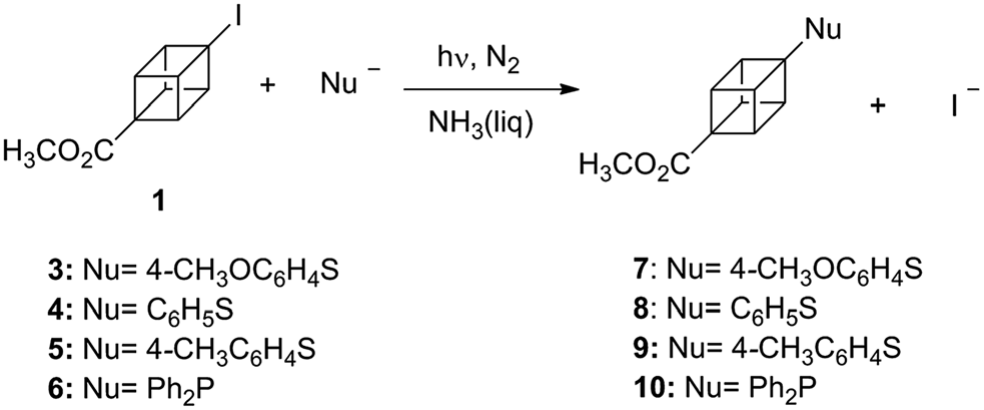
Photostimulated reactions of methyl-4-iodocubane-1-carboxylate (1) with aryl thiolates and Ph_2_P^−^ ions in NH_3(l)_.

Scheme 2 represents the reactions of substrate 1 with arylthiolate and diphenylphosphanide ions. The photoinitiated reaction of cubane 1 in the presence of 4-methoxybenzenethiolate (3^−^) led to the production of compound 7 in 61% isolated yield (Table 1, entry 1). The reaction did not take place without photostimulation since 95% of the substrate was recovered after 2 h (Table 1, entry 2). Besides, as expected in the presence a 20% mol *ca.* of radical [TEMPO] and radical anion [*m*-DNB] scavengers (Table 1, entries 3 and 4), yield of compound 7 was found to be lower (22 and 42%, respectively). These decreasing of yields indicate that a S_RN_1-type mechanism could be involved. Mechanism is represented in Scheme 4.

**Table 1:** Photostimulated reactions of methyl-4-iodocubane-1-carboxylate (1) with 4-methoxybenzenethiolate (3^−^), benzenethiolate (4^−^), 4-methylbenzenethiolate (5^−^) and diphenylphosphanide (6^−^)^a^

Entry	Nu^−^	Product (%), Nu–C_8_H_6_–COOCH_3_
1	*p*-CH_3_OC_6_H_4_S^−^ (3^−^)	7, 61 (78^b^)
2^c^		—
3^d^		7, 22^b^
4^e^		7, 42^b^
5	C_6_H_5_S^−^ (4^−^)	8, 75
6	*p*-CH_3_C_6_H_4_S^−^ (5^−^)	9, 49
7^f^	Ph_2_P^−^ (6^−^)	10, 43^b^

a Photostimulated reactions in NH_3(l)_ as solvent. Irradiation time = 2 h. [1] = 2.9 mM, [Nu^−^] = 14.5 mM. Isolated yields.

b The yield of the substituted product was determined by ^1^H-NMR with *p*-nitroacetophenone as internal standard.

c Dark reaction.

d To the reaction mixture was added 18 mol% of TEMPO.

e To the reaction mixture was added 20 mol% of *m*-dinitrobenzene (*m*-DNB).

f Irradiation time = 60 min, [6^−^] = 5.2 mM. It was isolated as the derivated acid.

We further explored the photoinitiated reactions of the cubane 1 with benzenethiol (4) and 4-methylbenzenethiol (5) which led to the substitution products in 75% (8) and 49% (9) yield, respectively (Table 1, entries 5 and 6). It had been already demonstrated that the Ph_2_P^−^ ion works well as nucleophile in the S_RN_1-type reactions.^1^ The reaction with the anion 6^−^, afforded the substituted methyl-4-(diphenylphosphino)cubane-1-carboxylate (10) in 43% yield (Table 1, entry 7).

**Scheme 3:**
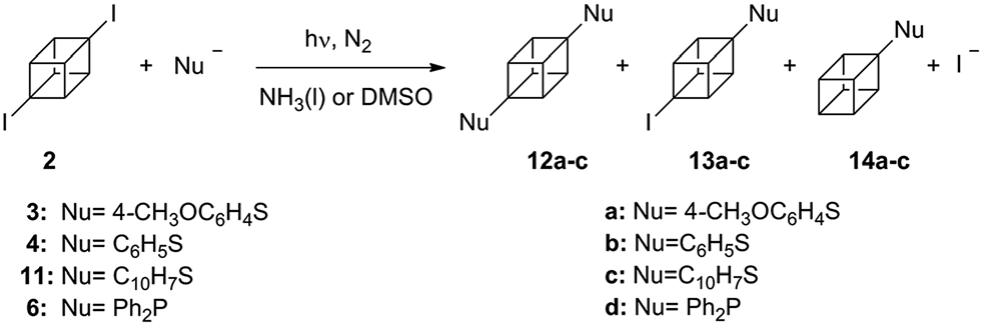
Photostimulated reactions of 1,4-diiodocubane (2) with arylthiolates and Ph_2_P^−^ (6^−^) ions in NH_3(l)_ or DMSO.

We next examined the diiodocubane (2) in similar reaction conditions. A special property of interest with this substrate was the potential capability to extend symmetrically over the 1,4-cubyl linear axis by two substitutions in a one-pot synthesis (Scheme 3).

**Scheme 4:**
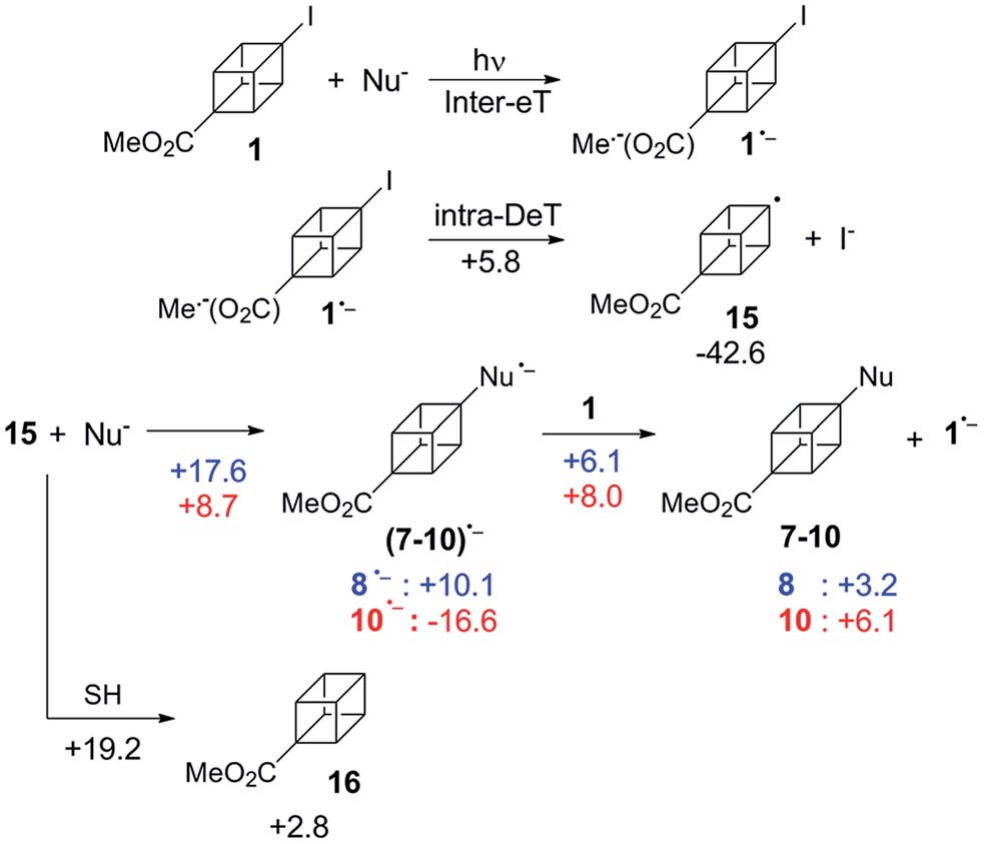
Proposed mechanism for the coupling between 1 and Nu^−^ (ArS^−^ and Ph_2_P^−^). The numbers are the computed Gibbs energies in [kcal mol^−1^] at the PCM-[M06-2X/def2-TZVP] level of theory. In blue are the energies for Nu^−^ = PhS^−^ and in red for Nu^−^ = Ph_2_P^−^. SH: the solvent considered is NH_3_.

The reaction indeed proceeded giving a mixture of disubstituted 1,4-bis((4-methoxyphenyl)thio)cubane (12a) and monosubstituted 4-methoxybenzenethiolcubane (14a) being formed in 48% and 10% yields, respectively (Table 2, entry 1). The substrate is photostable and under non-irradiated conditions (Table 2, entry 2), or in the presence of a good electron acceptor (*i.e. m*-DNB) afforded no products (Table 2, entry 3). When the nucleophile was the benzenethiolate (4^−^), reaction yields were quite similar to the ones in entry 1, for the di- (12b) and mono-substituted (14b) cubanes with 47% and 8%, respectively (Table 2, entry 6).

**Table 2:** Photoinitiated reactions of 1,4-diiodocubane (2) with 4-methoxybenzenethiolate (3^−^), benzenethiolate (4^−^), naphthalene-2-thiolate (11^−^) and diphenylphosphanide (6^−^)^a^

Entry	Nu^−^	Solv.	Yield (%)			
			2	12	13	14
1	*p*-CH_3_OC_6_H_4_S^−^ (3^−^)	NH_3(l)_	<5	12a, 48	—	14a, 10
2^b^		NH_3(l)_	90^c^	—	—	—
3^d^		NH_3(l)_	83^c^	—	—	—
4		DMSO	—	12a, 27	13a, 9	14a,11^e^
5^f^		DMSO	<5	12a, 39^g^	—	14a,10^e^
6	C_6_H_5_S^−^ (4^−^)	NH_3(l)_	14^e^	12b, 47	—	14b, 8
7		DMSO	—	12b, 19	13b, 15	14b, 8^e^
8^b^		DMSO	95	—	—	—
9	C_10_H_7_S^−^ (11^−^)	NH_3(l)_	73^c^	—	—	—
10		DMSO	59	—	13c, 12	—
11^h^	Ph_2_P^−^ (6^−^)	NH_3(l)_	—	—	—	14d, 75^c^

a Photostimulated reactions in NH_3(l)_ or DMSO_(l)_ as solvent. Irradiation time = 2 h. [2]_NH3(l)_ = 2.8 mM. [2]_DMSO_ = 28 mM, [Nu^−^]_NH3(l)_ = 17 mM. [Nu^−^]_DMSO_ = 168 mM. Isolated yields.

b Dark reaction.

c Quantified by ^1^H-NMR with *p*-nitroacetophenone as internal standard.

d 25 mol% of *m*-DNB was added to the reaction mixture.

e Yield determined by GC using the internal standard method.

f [2] = 2.8 mM.

g Products were quantified by HPLC using external standard method.

h [Nu^−^] = 11.7 mM.

**Fig. 1:**
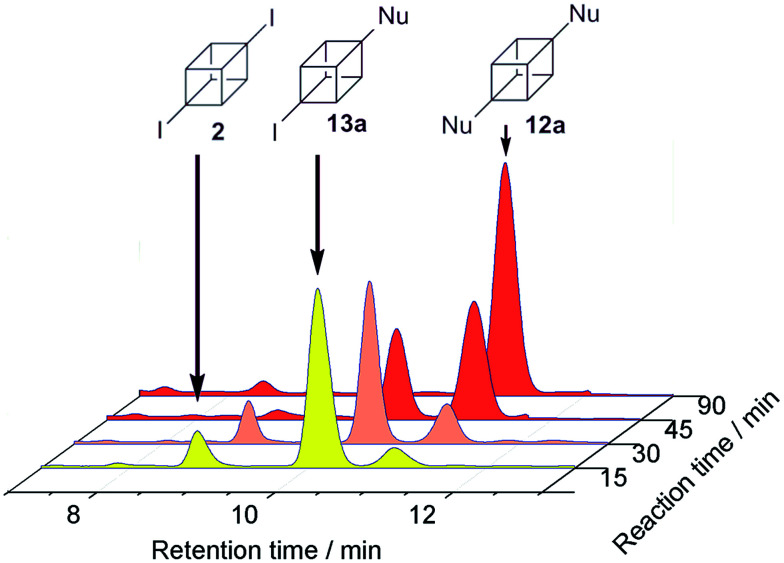
Photoinduced reaction between 2 and 3^−^ in DMSO analysed by RP-HPLC.

The reactions of 2 were also performed in DMSO. This solvent was chosen because the reaction setup becomes experimentally easier.^20^ Under photoinductive conditions with nucleophile 3^−^, not only 12a and 14a were observed, the intermediate (4-iodocuban-1-yl)(4-methoxyphenyl)sulfane (13a) was also detected in yields of 27%, 11%, and 9%, respectively (Table 2, entry 4). Aliquots of the reaction between nucleophile 3^−^ and cubane 2 in DMSO were collected at regular time intervals to analyze the reaction evolution (Fig. 1). After 15 minutes, both 13a (predominant compound) and 12a were observed, as well as substrate 2. After 45 minutes, the amount of 12a and 13a were observed in almost identical quantities, however after an additional 45 minutes of irradiation, 12a was the major product and 13a appeared only in trace amounts. When the reaction was carried out in diluted conditions in DMSO (same concentration of substrate than in NH_3(l)_), product 13a was not observed after 120 min of reaction (Table 2, entry 5). With nucleophile 4^−^, in DMSO, the presence of the similar intermediate 13b was found in 15% as well as the substituted products 12b (19%) and 14b (8%) (Table 2, entry 7). As expected, without irradiation, 95% of the substrate was recovered in the reaction in DMSO (Table 2, entry 8).

Previous studies have demonstrated that naphthalene-2-thiolate (11^−^) is a good nucleophile but at the same time is unable to transfer an electron to the aliphatic substrate in photoinduced electron transfer reactions.^3^ To facilitate reactions with good nucleophiles which are also poor electron donors, an entrainment compound can be added which is usually a good electron donor (reactive at initiation step) but kinetically slow for coupling as a nucleophile (non-reactive at propagation step).^1,21^ Aliphatic radicals, for example 1-haloadamantyl or cyclopropyl, were generated from aliphatic carbanions in S_RN_1-type reactions employing an entrainment agent, in DMSO^22^ as well as in NH_3(l)_^23^ as solvents. Therefore in the reactions of 11^−^ with 2, we employed the anions of acetone or pinacolone (3,3-dimethylbutan-2-one) as entrainments, but the coupling was still unsuccessful in NH_3(l)_. On the other hand, using DMSO as the solvent, and without any entrainment compound, it was possible to obtain 12% of the substituted compound with retention of the second leaving group 13c recovering 59% of substrate 2.

The possibility to obtain diphosphocubane was also explored by using Ph_2_P^−^ ion as nucleophile. The coupling did indeed occur, however, only 75% of monosubstituted-reduced product 14d was quantified by ^1^H-NMR spectroscopy.¶¶Compound 14d was difficult to isolated because it decomposes on silica gel as a similar reported phosphide cubanes derivatives (ref. 7*c*). This yield of the reduced product could be ascribed to the stronger reducing character of diphenylphosphanide with respect to that of the thiophenoxide ion; a difference of *ca.* 1.5 eV was estimated by DFT calculations (see ESI†).

In order to form a new C–C bond at the bridgehead position of the cubane nucleus, we conducted studies on the reactivity of the anion of nitromethane with 1 or 2. It is already known that this nucleophile can couple halo and dihaloadamantanes, among other halosubstrates, very efficiently but is unable to initiate the photostimulated S_RN_1 reaction.^1^ Indeed, no reaction was observed in our systems. This low reactivity for the eT nucleophilic substitution agrees with previous calculations on halo-bridgehead compounds. Pierini *et al.* proposed that an increase in the angular strain of aliphatic bridgehead substrates could involve a more negative reduction potential determined through their LUMO energies.^24^ It was also found that the 1-halocubanes studied are the halobridged substrates with lowest reactivity for photostimulated eT reactions.

### Calculation results

Based on the results of the photoinduced, dark, and inhibited reactions, we propose that 1 reacts with arylsulfide and diphosphide ions by the S_RN_1 mechanism, according to the mechanism proposed in Scheme 4.

Prior to the analysis of the energy profile of the proposed mechanism we carry out an evaluation of different DFT methods for the reaction between radical 15 and SPh^−^ ion, to select the most appropriate one. After a careful work and analysis we decided to choose the M06-2X method with def2-TZVP basis set, which is one of the methods that gave energetic barriers closer to those obtained with DPLNO-CCSD(T)/cc-pVTZ. Although this last method is the best evaluated method for its accuracy, it involves a higher computational cost (see Table in ESI, section 3.3.3).

The initiation step may follow an intermolecular eT from the excited state of the Nu^−^ to its CO_2_Me π-acceptor through a non-dissociative pathway with the intermediacy of 1˙^−^.||||The photoinduced process at the initiation step corresponds to the electron transfer from the excited state of the nucleophile to the substrate. One electron photoejection is reported for nucleophiles 3^−^, 4^−^ and 5^−^ when they are irradiated.^3^ UV-experiments of the mixture (substrate + arenethiolate ion) were carried out in order to observe if a charge transfer complex could be formed, but any new band in addition to those of the two reactants was observed. UV-spectra of the nucleophiles 4^−^ and 6^−^ are shown in the ESI,† as well as of the substrate 2. The experimental results suggest an efficient intramolecular-eT step from the π-acceptor to the C–I σ* bond through the aliphatic cubyl bridge. The calculations showed that the fragmentation of the 1˙^−^ into the radical 15 and I^−^, is favored by 42.6 kcal mol^−1^. An activation barrier for this intramolecular-eT of ∼5.8 kcal mol^−1^ was also obtained. This value of Δ*G*§ for the fragmentation process could be compared with those energies (intramolecular-eT from a carbonyl π-system to a C–X σ* bond) found for other constrained systems such as norbornyl, bicyclo[2,2,2]octane or adamantyl, which are always lower than 3 kcal mol^−1^.^25^ The higher energy found within the cubane system could be attributed to the lack of flexibility of the bridge which assists the intramolecular-eT process.

After the dissociation, the radical 15 couples with the Nu^−^ to form the radical anion of the product (7–10)˙^−^. According to our calculations, in terms of Gibbs energy, the process involves 10.1 kcal mol^−1^ and −16.6 kcal mol^−1^, for PhS^−^ and Ph_2_P^−^, respectively, and requires a barrier of 17.6 and 8.7 kcal mol^−1^ (in the same order) to be overcome.

The competitive reaction of 15 to produce the reduced product 16 is kinetically not favored with respect (Δ*G*§ = 19.2 kcal mol^−1^) to the coupling. Finally, an intermolecular eT from (7–10)˙^−^ to 1 is responsible to continue the propagation cycle to afford products (Scheme 4). The associated Gibbs energies are slightly endergonic, *i.e.*, 3.2 and 6.1 kcal mol^−1^ with and energy barrier of 6.1 and 8.0 kcal mol^−1^ for 8 and 10, respectively. Even though, the total energy released after fragmentation of the radical anion 1 (∼39 kcal mol^−1^) could be the driving force of the eT step.**This process could be even more favored if the eT takes place from the excited state of (7–10)˙^−^ to 1. However, due to the low concentration of these radical anions, it is difficult to determine if excited states participate in this eT step.

As it happens with other aliphatic systems,^1^ Ph_2_P^−^ is expected to be a better nucleophile than PhS^−^. Moreover, Ph_2_P^−^ is also a better reducing agent than PhS^−^, and that could justify why the yield of product 10 is lower than the yield of 8 (Table 1, entries 7 and 5). When the nucleophile acts as an electron donor, the substrate reduction process is favored in ∼32.7 kcal mol^−1^ for Ph_2_P^−^ (Ph_2_P^−^/Ph_2_P˙ ≈ 82.7 kcal mol^−1^ or 3.58 eV) compared with PhS^−^ (PhS^−^/PhS˙ ≈ 115.4 kcal mol^−1^ or 5.05 eV).

Although our reactions were carried out under eT conditions, other possible pathways for the formation of the substituted products were evaluated. Calculations indicated that the preferred reaction is the coupling with the nucleophile to follow a typical S_RN_1 pathway. Relevant energetic factors of the mechanisms explored are presented in Scheme 1-SI and Table 1-SI of the ESI.†

**Scheme 5:**
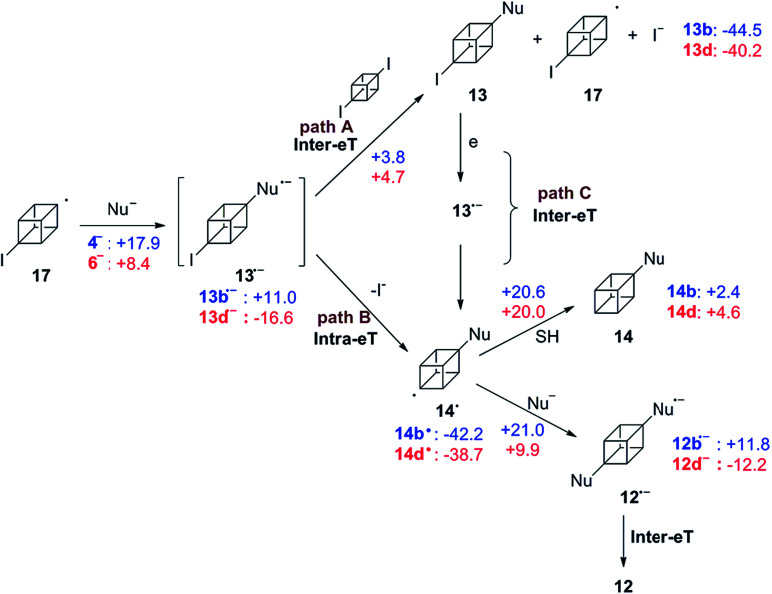
Proposed mechanism for the coupling between 2/17 and Nu^−^ (PhS^−^ and Ph_2_P^−^). The numbers are the computed Gibbs energies in [kcal mol^−1^] at the PCM-[M06-2X/def2-TZVP] level of theory. In blue are the energies for Nu^−^ = PhS^−^ and in red for Nu^−^ = Ph_2_P^−^. SH: the solvent considered is NH_3_.

Similarly, the S_RN_1 mechanism is proposed for substrate 2 in the photoinduced coupling reactions with arylthiolate and diphenylphosphanide ions as nucleophiles. Once radical 17 is formed at initiation step, it couples with the nucleophile PhS^−^ affording the radical anion 13b˙^−^. It is proposed that this radical anion could transfer its extra electron according to two paths represented in the mechanism shown in Scheme 5. Path A depicts the process when the electron transfer is intermolecular from 13b˙^−^ to 2 to obtain the isolated compound 13b and the radical of the substrate which is responsible for continuing the propagation chain. In path B the electron transfer could be an intramolecular process between the π acceptor (ArS) to the C–I σ* bond through the aliphatic cubyl bridge.

As is observed in Fig. 1, product 13b is the first compound generated at the beginning of the reaction, when the reaction keeps going, the signal of product 12b increases. At the end, 12b is the main product observed and also traces of 13b. This fact could indicate that the Inter-eT (path A) should predominate compared to the Intra-eT (path B) from 13b˙^−^. According to our calculations, path A is thermodynamically favored by 2.3 kcal mol^−1^ over path B. After formation of compounds 13b, these could start a new chain mechanism, involving an Inter-eT from the nucleophile to generate the radical 14b˙ (path C) which continues to finally obtain the disubstituted or the monosubstituted-reduced compounds. We were unable to obtain experimental evidence to determine if path B does occur or not. Even when carrying out the reaction at shorter times, compound 12b was already generated as well as 13b.††††Even when a low energetic barrier for the fragmentation of 13b˙^−^ into radical 14 and iodide ion is expected, since we could not find the geometry of the transition state, this value is not included in the scheme. After an exploration of the reaction pathway an estimated value close to 3.8 kcal mol^−1^ was found, which is comparable to that found for the eT in path A, *i.e.*, 3.8 kcal mol^−1^.

Differences with the ArS^−^ behavior are observed when the nucleophile is Ph_2_P^−^ (6). As it was mentioned previously, Ph_2_P^−^ is a good reducing agent and based on the fact that 14d, the monosubstituted-reduced compound, is the major product (Table 2, entry 11), we can postulate that path B is the most efficient electron transfer process. Similar results were observed for the rigid substrate 1,2-dichloroadamantane in its reaction with 6^−^,^1^ and no disubstitution product was observed. The reduction of radical 14d˙ to give 14d seems to be effective and two possibilities are proposed about this formation. One possibility is that an eT occurs from the nucleophile to the radical followed by protonation in the reaction workup, which has been proposed elsewhere.^16*a*^ The other possibility is a hydrogen abstraction from the solvent to the radical. However, liquid ammonia is not a good hydrogen donor, it can be seen from the Gibbs energy barriers informed in Scheme 5. Although the computed energies suggest that the disubstituted compounds 12b and 12d can be obtained, only the first one was observed in the experiments. Therefore, the reduction of 14d˙^−^*via* formation of an anion followed by proton abstraction during the reaction workup seems to be a plausible explanation.

## Conclusions

In summary, we have developed a mild synthetic protocol to introduce thioaryl and diphenylphosphine moieties onto the cubane core *via* S_RN_1 mechanism with moderate to good yields. The mono-halosubstituted cubane derivative used (1) affords the substitution reaction of the halogen atom in moderate yields. In the case of the dihalosubstituted cubane (2), different products can be obtained depending on the conditions employed. When arenethiolate nucleophiles are used, three products can be formed, namely mono-substituted, di-substituted, and mono-substituted with retention of a halogen atom. Their relative yield can be controlled by the reaction conditions. On the other hand, the use of diphenyl phosphine as the nucleophile precursor leads only to the mono-substituted cubane derivative. This could be ascribed to the higher reduction power of this nucleophile.

The experimental evidences collected give hints of a reaction where radical species are involved. DFT calculations supports an operating S_RN_1 reaction mechanism where the rate determining steps are the nucleophilic coupling into the cubyl radical intermediate.

## Experimental

### Materials

Potassium *tert*-butoxide, 4-methoxybenzenethiol (3), benzenethiol (4), naphthalene-2-thiol (11) and potassium diphenylphosphide solution (0.5 M in THF) are commercially available and used as received. Methyl-4-iodocubane-1-carboxylate (1) and 1,4-diiodocubane (2) were synthesized according to ref. 18. DMSO is Carlo Erba and stored under molecular sieves (4 Å). ^1^H NMR and ^13^C NMR spectra were recorded on a 400 MHz Bruker nuclear magnetic resonance spectrometer. HR-MS were recorded on a Bruker, MicroTOF Q II equipment, operated with an ESI source in (positive/negative) mode, using nitrogen as nebulizing and drying gas and sodium formate 10 mM as internal standard. Gas chromatographic analyses were performed on a Varian 3900 GC with flame ionization detector on a FactorFour capillary column (VF-5 MS, 30 m, 0.32 mm, 0.25 micron). GC-MS analyses were carried out on a Shimadzu GC-MS QP5050 spectrometer, employing a 30 m, 0.32 mm, 0.25 micron, DB-5 MS column. Irradiation was performed in a reactor equipped with two 400 W lamps (Philips model Master HPI-T Plus, air- and water-cooled). The Fig. SI-1 in ESI† shows the spectrum of the lamps. HPLC analyses were carried out on a Waters 1525 Binary HPLC Pump connected to a Waters 2998 Photodiode Array Detector, and employing an Agilent Zorbax Eclipse XDB-C18 Analytical column (4.6 × 150 mm, 5 μm).

### Photoinitiated reaction in NH_3(l)_, (*T* = −33 °C)

The following procedure is representative for all reactions in NH_3(l)_ as solvent. The equipment used is a close system, composed by a 100 mL three-necked round bottomed pyrex-flask with a Dewar condenser, N_2_ inlet and NH_3_ inlet (see Picture SI-2 in ESI†), was dried under vacuum. Liquid ammonia (100 mL), previously dried over Na metal, was distilled into the flask under nitrogen atmosphere (see Picture SI-3–5 in ESI†). Potassium *tert*-butoxide and then the precursor of the nucleophile were added to the distilled ammonia. After 15 min, the cubane substrate was dissolved (1 mL) in freshly distilled THF and then added to the mixture. The reaction mixture was irradiated for 120 min. In dark reactions (without irradiation), the reaction flask was protected from light with aluminium foil and keep under N_2_ atmosphere (Picture SI-6 in ESI†).

After 2 hours, the mixture was quenched with an excess of CH_3_I and NH_4_NO_3_. The ammonia was allowed to evaporate, and acidic deionized water (50 mL) was added to the residue and extracted twice with diethyl ether (30 mL). The organic extract was dried (Na_2_SO_4_) and filtered. The solvent was removed under reduced pressure before separation by column chromatography.

### Photoinduced reactions in DMSO, (*T* = 30 °C)

Into a previously dried 20 mL Schlenk-type flask (Pyrex) equipped with nitrogen inlet and magnetic stirrer, 10 mL of dried DMSO stored under molecular sieves (4 Å) was added. The solvent was degassed three times under vacuum and stirring, interspersed with N_2_. Afterwards, potassium *tert*-butoxide and the anion source were added and 5 min later, the corresponding amount of cubane substrate dissolved in freshly distilled THF (0.5 mL) was added. The mixture was irradiated for 120 min then quenched by the addition of CH_3_I, NH_4_NO_3_, and deionized water. In dark reactions (without irradiation), the reaction flask was protected from light with aluminium foil and keep under N_2_ atmosphere. Water at pH < 3 is added followed by extraction with diethyl ether (3 × 20 mL). The ether extract was washed until no residual of DMSO remained. The organic extract was dried with Na_2_SO_4_, filtered, and evaporated under reduced pressure.

### Computational procedure

All the calculations were performed with the Gaussian09 program,^26^ the M06-2X DFT functional and the def2-TZVP basis set for C, H, O, S and P were employed. The def2-TZVP^27^ basis set and pseudo-potential was used for I. Calculations were performed with full geometry optimization including in all cases the effect of the solvent (methanol as polar solvent) through the Tomasi's polarized continuum model (IEFPCM)^28,29^ as implemented in the Gaussian package. After refinement the characterization of stationary points was done by Hessian matrix calculations, with all positive eigenvalues for a minimum and only one negative eigenvalue for the TSs. The energy informed for TSs and radicals includes zero-point corrections.

### Methyl-4-((4-methoxyphenyl)thio)cubane-1-carboxylate (7)

Compound was isolated by semi-preparative HPLC, employing a constant flow of 2.0 mL min^−1^ of a mixture of hexane/ethyl acetate (90 : 10). Melting point: 210 °C with decomposition. Yield: 61% (54.9 mg). ^1^H NMR (400 MHz, acetone-d_6_): *δ* 7.28 (d, 2H, H_Ar_); 6.93 (d, 2H, H_Ar_); 4.15–4.12 (m, 3H, H_cubyl_); 3.98–3.95 (m, 3H, H_cubyl_); 3.80 (s, 3H, CH_3_); 3.65 (s, 3H, CH_3_). ^13^C NMR (acetone-d_6_): *δ* 171.9 (q, C=O); 160.3 (q, C_Ar_–O); 133.8 (2C, C_Ar_–H); 125.0 (q, C_Ar_–S); 115.7 (2C, C_Ar_–H); 62.5 (q, C_cubyl_–S); 57.3 (q, C_cubyl_); 55.7 (CH_3_); 51.6 (CH_3_); 50.2 (3C, C_cubyl_–H); 47.1 (3C, C_cubyl_–H). For more details in the assignment and 2D NMR experiments, see ESI.† IR (neat): *ν* = 2993, 2946, 1724 (C=O), 1581, 1479, 1436, 1325, 1228, 1198, 1089, 837, 739 and 691 cm^−1^. HRMS (ESI-TOF) *m*/*z*: [M + H]^+^ calcd. for C_17_H_17_O_3_S 301.0893; found 301.0913.

### Methyl-4-(phenylthio)cubane-1-carboxylate (8)

The compound was isolated by semi-preparative HPLC, employing a constant flow of 2.0 mL min^−1^ of a mixture of hexane/ethyl acetate (90 : 10). Melting point: 198 °C with decomposition. Yield: 75% (60.8 mg). ^1^H NMR (400 MHz, acetone-d_6_): *δ* 7.35–7.32 (m, 2H, H_Ar_); 7.24–7.20 (m, 3H, H_Ar_); 4.25–4.22 (m, 3H, H_cubyl_); 4.07–4.04 (m, 3H, H_cubyl_); 3.66 (s, 3H, CH_3_). ^13^C NMR (acetone-d_6_): *δ* 171.9 (q, C=O); 136.2 (q, C_Ar_–S); 130.7 (2C, C_Ar_–H); 129.7 (C_Ar_–H); 126.9 (2C, C_Ar_–H); 61.3 (q, C_cubyl_–S); 56.9 (q, C_cubyl_); 51.7 (CH_3_); 50.3 (3C, C_cubyl_–H); 47.4 (3C, C_cubyl_–H). For more details in the assignment and 2D NMR experiments, see ESI.† HRMS (ESI-TOF) *m*/*z*: [M + H]^+^ calcd. for C_16_H_15_O_2_S 271.0787; found: 271.0787.

### Methyl-4-(4-methylphenylthio)cubane-1-carboxylate (9)

The compound was isolated from the crude by column chromatography, employing a linear gradient of eluent composed of pentane and diethyl ether, from 0% to 30% of diethyl ether. Melting point: 201 °C with decomposition. Yield: 49% (41.8 mg). ^1^H NMR (400 MHz, acetone-d_6_): *δ* 7.15 (d, *J* = 2.2 Hz, 4H); 4.21–4.18 (m, 3H, H_cubyl_); 4.02–3.99 (m, 3H, H_cubyl_); 3.65 (s, 3H, CH_3_); 2.29 (s, 3H, CH_3_). ^13^C NMR (acetone-d_6_): *δ* 171.9 (q, C=O); 137.0 (q, C_Ar_–S); 132.1 (q, C_Ar_); 130.7 (2C, C_Ar_–H); 130.5 (2C, C_Ar_–H); 61.6 (q, C_cubyl_–S); 56.9 (q, C_cubyl_); 51.6 (OCH_3_); 50.3 (C_cubyl_–H); 47.3 (C_cubyl_–H), 20.9 (CH_3_). HRMS (ESI-TOF) *m*/*z*: [M + H]^+^ calcd. for C_17_H_17_O_2_S 285.0943; found: 285.0943.

### 4-(Diphenylphosphoryl)cubane-1-carboxylic acid (10a)

The cubane analogue was isolated as the derivated acid form of the product 9 from the crude by column chromatography, employing a linear gradient of eluent composed by pentane and diethyl ether, from 10% to 100% of diethyl ether. Yield: 21% (21.9 mg). ^1^H NMR (400 MHz, dimethyl sulfoxide-d_6_): *δ* 12.46 (bb, 1H, OH); 7.62–7.54 (m, 10H); 4.21–4.19 (m, 6H, H_cubyl_). ^13^C NMR (dimethyl sulfoxide-d_6_): *δ* 171.7 (q, C=O); 131.9 (2C, d, ^4^*J*_P-C_ = 2.4 Hz, C_Ar_–H); 131.7 (2C, d, ^1^*J*_P-C_ = 98.4 Hz, q); 130.4 (4C, d, ^3^*J*_P-C_ = 9.3 Hz, C_Ar_–H); 128.9 (4C, d, ^2^*J*_P-C_ = 11.3 Hz, C_Ar_–H); 55.5 (d, ^4^*J*_P-C_ = 4.3 Hz, q, C_cubyl_); 52.4 (d, ^1^*J*_P-C_ = 67.4 Hz, q, C_cubyl_); 47.5 (3C, d, ^2^*J*_P-C_ = 8.2 Hz, C_cubyl_–H); 44.3 (3C, d, ^3^*J*_P-C_ = 5.2 Hz, C_cubyl_–H). HRMS (ESI-TOF) *m*/*z*: [M − H]^−^ calcd. for C_21_H_16_O_3_P– 347.0843; found: 347.0839.

### 1,4-Bis((4-methoxyphenyl)thio)cubane (12a)

Isolated as a white solid from the crude by column chromatography, employing a linear gradient of eluent composed by pentane and diethyl ether, from 0% to 30% of diethyl ether. Melting point: 237 °C with decomposition. Yield: 48% in NH_3(l)_ (51.3 mg). ^1^H NMR (400 MHz, CDCl_3_): *δ* 7.22 (d, *J* = 8.8 Hz, 4H); 6.83 (d, *J* = 8.8 Hz, 4H); 3.93 (s, 6H, H_Cubyl_); 3.78 (s, 6H, CH_3_). ^13^C NMR (CDCl_3_): *δ* 159.1 (q, 2C, C_Ar_–O); 132.9 (4C, C_Ar_–H); 124.6 (2C, q, C_Ar_–S); 114.7 (4C, C_Ar_–H); 61.8 (2C, q, C_cubyl_–S); 55.4 (2C, CH_3_); 49.0 (6C, C_cubyl_–H). HRMS (ESI-TOF) *m*/*z*: [M + Na]^+^ calcd. for C_22_H_20_O_2_S_2_Na 403.0797; found 403.0797.

### 1,4-Bis(phenylthio)cubane (12b)

The cubane derivative was isolated as a white solid from the crude by column chromatography. Employed was a linear gradient of eluent composed by pentane and diethyl ether, from 0% to 20% of diethyl ether. Melting point: 230 °C with decomposition. Yield: 47% (42.3 mg). ^1^H NMR (400 MHz, CDCl_3_): *δ* 7.30–7.26 (m, 4H); 7.20–7.16 (m, 6H); 4.09 (s, 6H, H_Cubyl_). ^13^C NMR (CDCl_3_): *δ* 135.5 (2C, q, C_Ar_–S); 129.2 (4C, C_Ar_–H); 129.1 (4C, C_Ar_–H); 126.1 (2C, C_Ar_–H); 60.5 (2C, q, C_cubyl_–S); 49.5 (6C, C_cubyl_–H). IR (neat): *ν* = 3072, 3059, 2990, 1582, 1480, 1435, 1191, 1088, 1069, 732 and 691 cm^−1^. HRMS (ESI-TOF) *m*/*z*: [M + Na]^+^ calcd. for C_20_H_16_S_2_Na 343.0586; found 343.0586.

### (4-Iodocuban-1-yl)(4-methoxyphenyl)sulfane (13a)

Isolated as a pale yellow solid from the crude by column chromatography, employing a linear gradient of eluent composed by pentane and diethyl ether, from 0% to 30% of diethyl ether. Melting point: 256 °C with decomposition. Yield: 9% (9.9 mg). ^1^H NMR (400 MHz, CDCl_3_): *δ* 7.21 (d, *J* = 8.7 Hz, 2H); 6.84 (d, *J* = 8.7 Hz, 2H); 4.22–4.20 (m, 3H, H_Cubyl_); 4.13–4.11 (m, 3H, H_Cubyl_); 3.80 (s, 3H, CH_3_). ^13^C NMR (CDCl_3_): *δ* 159.3 (q, C_Ar_–O); 133.2 (2C, C_Ar_–H); 124.0 (q, C_Ar_–S); 114.9 (2C, C_Ar_–H); 62.2 (q, C_cubyl_–S); 55.5 (CH_3_); 54.4 (3C, C_cubyl_–H); 52.6 (3C, C_cubyl_–H), 37.1 (q, C_cubyl_–I). HRMS (ESI-TOF) *m*/*z*: [M + H]^+^ calcd. for C_15_H_14_IOS 368.9805; found: 368.9805.

### (4-Iodocuban-1-yl)(phenyl)sulfane (13b)

The cubane compound was isolated as a pale yellow solid from the crude by column chromatography, employing a linear gradient of eluent composed by pentane and diethyl ether, from 0% to 20% of diethyl ether. Melting point: 251 °C with decomposition. Yield: 15% (14.2 mg). ^1^H NMR (400 MHz, CDCl_3_): *δ* 7.30–7.13 (m, 5H); 4.31–4.28 (m, 3H, H_Cubyl_); 4.23–4.20 (m, 3H, H_Cubyl_). ^13^C NMR (CDCl_3_): *δ* 135.1 (q, C_Ar_–S); 129.25 (2C, C_Ar_–H); 129.23 (2C, C_Ar_–H); 126.3 (C_Ar_–H); 61.1 (q, C_cubyl_–S); 54.7 (3C, C_cubyl_–H); 52.7 (3C, C_cubyl_–H); 36.5 (q, C_cubyl_–I). HRMS (ESI-TOF) *m*/*z*: [M + H]^+^ calcd. for C_14_H_11_IS 338.9699; found 338.9683.

### (4-Iodocuban-1-yl)(naphthalen-2-yl)sulfane (13c)

Isolated as a white solid from the crude by semi-preparative HPLC, employing as eluent of acetonitrile/water at a ratio of 90 : 10 and constant flow of 2 mL min^−1^. Melting point: 261 °C with decomposition. Yield: 12% (13.1 mg). ^1^HNMR (400 MHz, CDCl_3_): *δ* 7.80–7.70 (m, 3H); 7.52 (s, 1H); 7.50–7.42 (m, 2H); 7.29–7.26 (m, 1H); 4.35–4.33 (m, 3H, H_cubyl_); 4.29–4.27 (m, 3H, H_cubyl_). ^13^C-NMR (CDCl_3_): *δ* 133.9 (q, C_Ar_–S); 132.6 (q, C_Ar_); 131.9 (q, C_Ar_); 128.8 (C_Ar_–H); 127.9 (C_Ar_–H); 127.4 (C_Ar_–H); 127.3 (C_Ar_–H); 127.3 (C_Ar_–H); 126.8 (C_Ar_–H); 125.9 (C_Ar_–H); 61.2 (q, C_cubyl_–S); 54.8 (3C, C_cubyl_–H); 52.8 (3C, C_cubyl_–H); 36.5 (q, C_cubyl_–I). HRMS (ESI-TOF) *m*/*z*: [M + H]^+^ calcd. for C_18_H_14_IS 388.9855; found: 388.9858.

### Cuban-1-yl(4-methoxyphenyl)sulfane (14a)

Isolated as a white solid from the crude by column chromatography, employing a linear gradient of eluent composed by pentane and diethyl ether, from 0% to 30% of diethyl ether. Melting point: 226 °C with decomposition. Yield: 10% (6.8 mg). ^1^H NMR (400 MHz, CDCl_3_): *δ* 7.20 (dt, *J* = 8.8 Hz, 2H); 6.84 (dt, *J* = 8.8 Hz, 2H); 4.07–4.00 (m, 4H, H_Cubyl_); 3.99–3.95 (m, 3H, H_Cubyl_); 3.79 (s, 3H, CH_3_). ^13^C NMR (CDCl_3_): *δ* 158.8 (q, C_Ar_–O); 132.3 (2C, C_Ar_–H); 125.7 (q, C_Ar_–S); 114.7 (2C, C_Ar_–H); 60.9 (q, C_cubyl_–S); 55.4 (CH_3_); 52.2 (3C, C_cubyl_–H); 48.3 (q, C_cubyl_–S); 44.8 (3C, C_cubyl_–H). For more details in the assignment and 2D NMR experiments, see ESI.† HRMS (ESI-TOF) *m*/*z*: [M + H]^+^ calcd. for C_15_H_15_OS 243.0838; found: 243.0853.

### Cuban-1-yl(phenyl)sulfane (14b)

Isolated as a pale brown solid from the crude by column chromatography, employing a linear gradient of eluent composed by pentane and diethyl ether, from 0% to 20% of diethyl ether. Melting point: 300 °C with decomposition. Yield: 8% (5.4 mg). ^1^H NMR (400 MHz, CDCl_3_): *δ* 7.29–7.25 (m, 2H); 7.17–7.13 (m, 3H); 4.12–4.05 (m, 7H, H_Cubyl_). ^13^C NMR (CDCl_3_): *δ* 136.6 (q, C_Ar_–S); 129.1 (2C, C_Ar_–H); 128.5 (2C, C_Ar_–H); 125.5 (C_Ar_–H); 57.0 (q, C_cubyl_–S); 52.3 (3C, C_cubyl_–H); 47.9 (C_cubyl_–H); 45.1 (3C, C_cubyl_–H). HRMS (ESI-TOF) *m*/*z*: [M + H]^+^ calcd. for C_15_H_15_OS 243.0838; found: 243.0853.

### Cuban-1-yl-diphenylphosphine oxide (14d)

The cubane derivative was isolated as a white solid from the crude by column chromatography (gradient from pentane to ethyl acetate 100%) followed by semi-preparative HPLC, employing a mixture of pentane/isopropanol (gradient from 100% of pentane to 40% isopropanol at the end) at constant flow of 2 mL min^−1^. Melting point: nd. >300 °C. Yield: 18% (15.4 mg). ^1^H NMR (400 MHz, CDCl_3_): *δ* 7.64–7.59 (m, 4H); 7.54–7.50 (m, 2H); 7.47–7.43 (m, 4H); 4.35–4.30 (m, 3H, H_Cubyl_); 4.12–4.05 (m, 4H, H_Cubyl_). ^13^C NMR (CDCl_3_): *δ* 132.0 (2C, d, ^1^*J*_P-C_ = 99.5 Hz, q, C_Ar_); 131.8 (2C, d, ^4^*J*_P-C_ = 2.7 Hz, C_Ar_–H); 131.0 (4C, d, ^3^*J*_P-C_ = 9.4 Hz, C_Ar_–H); 128.8 (4C, d, ^2^*J*_P-C_ = 11.5 Hz, C_Ar_–H); 52.5 (d, ^1^*J*_P-C_ = 66.1 Hz, q, C_cubyl_); 47.9 (d, ^4^*J*_P-C_ = 5.0 Hz, C_cubyl_–H); 47.8 (3C, d, ^3^*J*_P-C_ = 5.8 Hz, C_cubyl_–H); 46.8 (3C, d, ^2^*J*_P-C_ = 8.0 Hz, C_cubyl_–H). IR (neat): *ν* = 3056, 2989, 1437 (P-C deformation band), 1220, 1178, 1116, 722, 700, 554 and 523 (cm^−1^). HRMS (ESI-TOF) *m*/*z*: [M + H]^+^ calcd. for C_20_H_18_OP 305.1090; found: 305.1086.

## Conflicts of interest

There are no conflicts to declare.

## Supplementary Material

RA-008-C8RA06275G-s001
