# Supplementary material for: Photoinduced nucleophilic substitution of iodocubanes with arylthiolate and diphenylphosphanide ions. Experimental and computational approaches
Source: RSC Adv. 2018 Nov 23;8(69):39222–30. doi: 10.1039/c8ra06275g (PMC9090936; doi:10.1039/c8ra06275g)
Supplement: RA-008-C8RA06275G-s001 [file RA-008-C8RA06275G-s001.pdf]

Electronic Supplementary Material (ESI) for RSC Advances.
This journal is © The Royal Society of Chemistry 2018

Photoinduced Nucleophilic Substitution of Iodocubanes with Arylthiolate and Diphenylphosphanide ions. Experimental and Computational Approaches

Liliana B. Jimenez, Marcelo Puiatti, Diego Andrada,[‡] Federico Brigante, Karina F. Crespo Andrada, Roberto A. Rossi, Ronny Priefer,[§] Adriana B. Pierini[#]*

INFIQC, Departamento de Química Orgánica, Facultad de Ciencias Químicas, Universidad Nacional de Córdoba, Ciudad Universitaria, X5000HUA Córdoba, Argentina.

[‡]Krupp-Professur für Allgemeine und Anorganische Chemie, Universität des Saarlandes, 66123 Saarbrücken, Germany.

[§]College of Pharmacy, Western New England University, Springfield, Massachusetts 01119, United States

[#] Prof. Adriana B. Pierini deceased

SUPPORTING INFORMATION

1. Experimental Information

1.1	Figure **SI-1**: Spectrum of lamps used for photoinduced $S_{RN}1$ reactions	S1
	Pictures **SI-2-6**: Experimental device for the $NH_{3(l)}$ reactions	S1-S3
1.2	Figure **SI-7**: UV-visible spectra of **2**, **4**$^-$ and **6**$^-$	S4-S5

2. NMR spectra

2.1	^{1}H, ^{13}C and 2D NMR spectra of **Methyl 4-((4-methoxyphenyl)thio)cubane-1-carboxylate (7)**	S6-S10
2.2	^{1}H, ^{13}C, 2D NMR and IR spectra of **Methyl 4-(phenylthio)cubane-1-carboxylate (8)**	S11-S16
2.3	^{1}H and ^{13}C of **Methyl 4-(4-methylphenylthio)cubane-1-carboxylate (9)**	S17-S18
2.4	^{1}H and ^{13}C of **4-(diphenylphosphoryl)cubane-1-carboxylic acid (10a)**	S19-S21
2.5	^{1}H and ^{13}C of **1,4-bis((4-methoxyphenyl)thio)cubane (12a)**	S22-S23
2.6	^{1}H, ^{13}C and IR spectra of **1,4-bis(phenylthio)cubane (12b)**	S24-S26
2.7	^{1}H and ^{13}C spectra of **(4-iodocuban-1-yl)(4-methoxyphenyl)sulfane (13a)**	S27-S28
2.8	^{1}H and ^{13}C spectra of **(4-iodocuban-1-yl)(phenyl)sulfane (13b)**	S29-S30
2.9	^{1}H and ^{13}C spectra of **(4-iodocuban-1-yl)(naphthalen-2-yl)sulfane (13c)**	S31-S32
2.10	^{1}H, ^{13}C and 2D NMR spectra of **Cuban-1-yl(4-methoxyphenyl)sulfane (14a)**	S33-S37
2.11	^{1}H and ^{13}C of **Cuban-1-yl(phenyl)sulfane (14b)**	S38-S39
2.12	^{1}H, ^{13}C and IR spectra of **Cuban-1-yl diphenylphosphine oxide (14d)**	S40-S42

3. Computational Information

3.1	Test of different solvent´s models	S43
3.2	Calculation of the redox potentials. Geometries. Spin densities of radicalary species.	S43-S45
3.3	Description of the hypothetical formation of 1,4-dehydrocubane **A**	S45-S47
	References	S48
3.4	Calculations involved in Schemes 4 and 5	S49-S77
3.4.1	Cartesian coordinates and energies (au) at PCM(methanol)-[M06-2X/def2-TZVP]. Spin densities of radicalary species. Imaginary frequencies of Transition States.	S49
3.4.2	HOMO-LUMO Levels of selected compounds	S73
3.4.3	Estimation of the Activation barrier by Marcus and Savéant’s models	S75
3.4.3	Table 1. DFT functional and post-HF methods for Activation Gibb Energies ($\Delta G^‡$, in kcal/mol), Activation Enthalpy ($\Delta H^‡$, in kcal/mol). Kinetic Parameters.	S76-S77

1. *Experimental Information*

1.1. Experimental system description for $NH_{3(l)}$ reaction

- Figure **SI-1**: Spectrum of lamps used for photoinduced $S_{RN}1$ reactions. It was obtained from the manufacturer webpage (http://www.lighting.philips.com.eg/prof/lamps/high-intensity-discharge-lamps/quartz-metal-halide/master-hpi-t-plus/928481600096_EU/product).

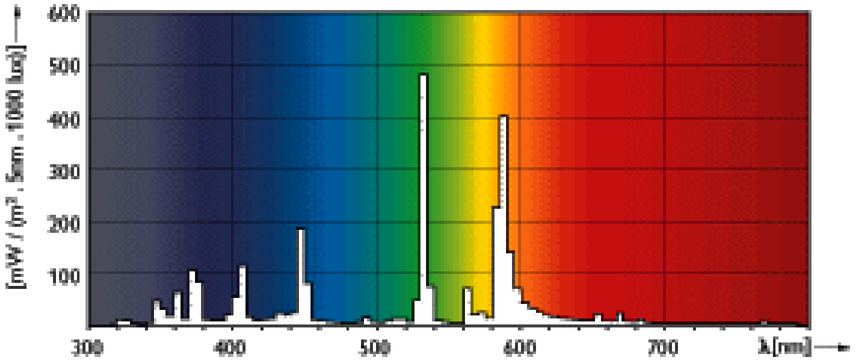

- Picture **SI-2**: Experimental device for the $NH_{3(l)}$ reactions

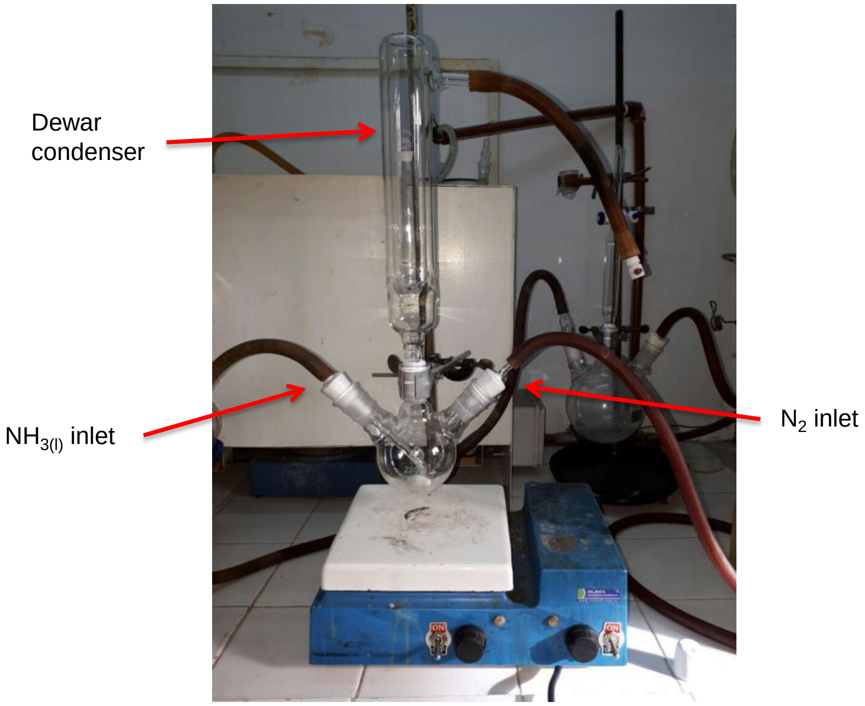

- Picture **SI-3**: Experimental device for the $NH_{3(l)}$ reactions. Solvent drying process.

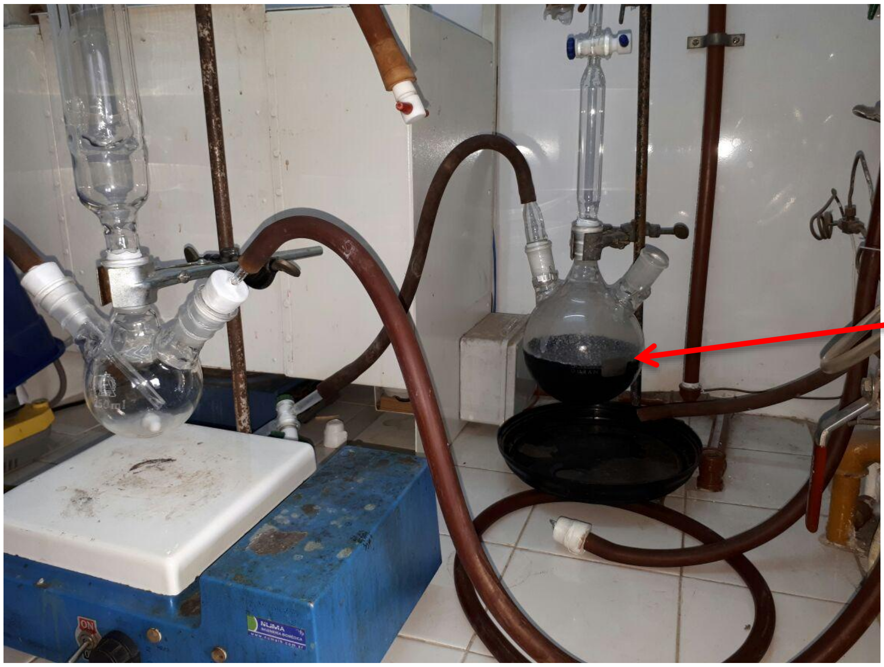

$NH_{3(l)}$ with Na before to distill it

- Picture **SI-4**: Experimental device for the $NH_{3(l)}$ reactions. Distillation of $NH_{3(l)}$ dry.

N_2(l)- ethanol bath within the coldfinger's Dewar condenser

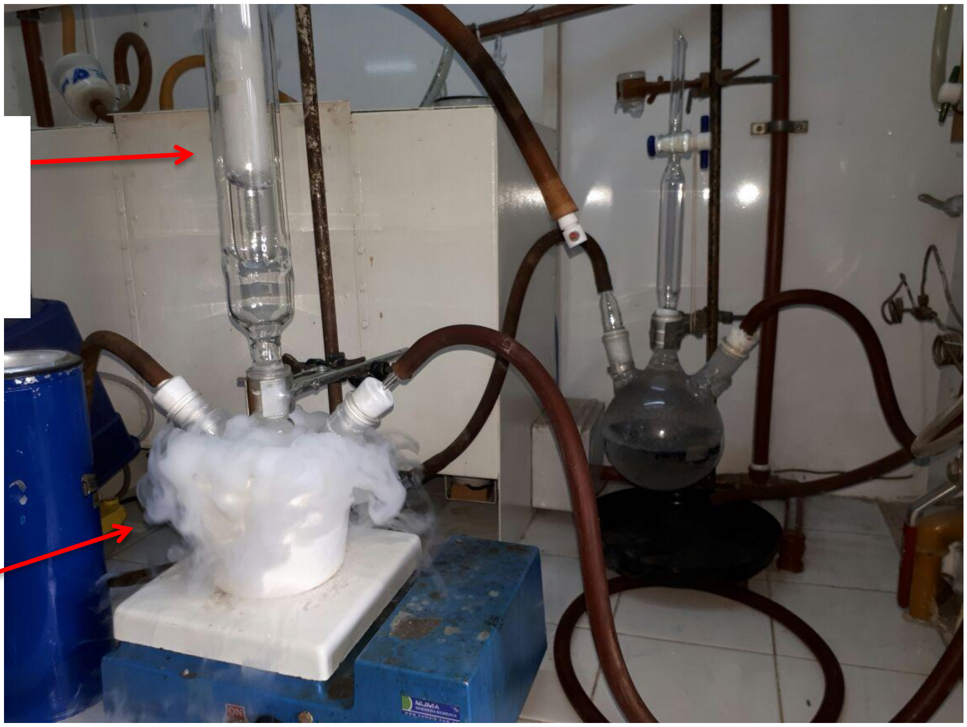

N_2(l)- ethanol bath

-Picture **SI-5**: Experimental device for the $NH_{3(l)}$ reactions. Distilled $NH_{3(l)}$ before adding the substrates.

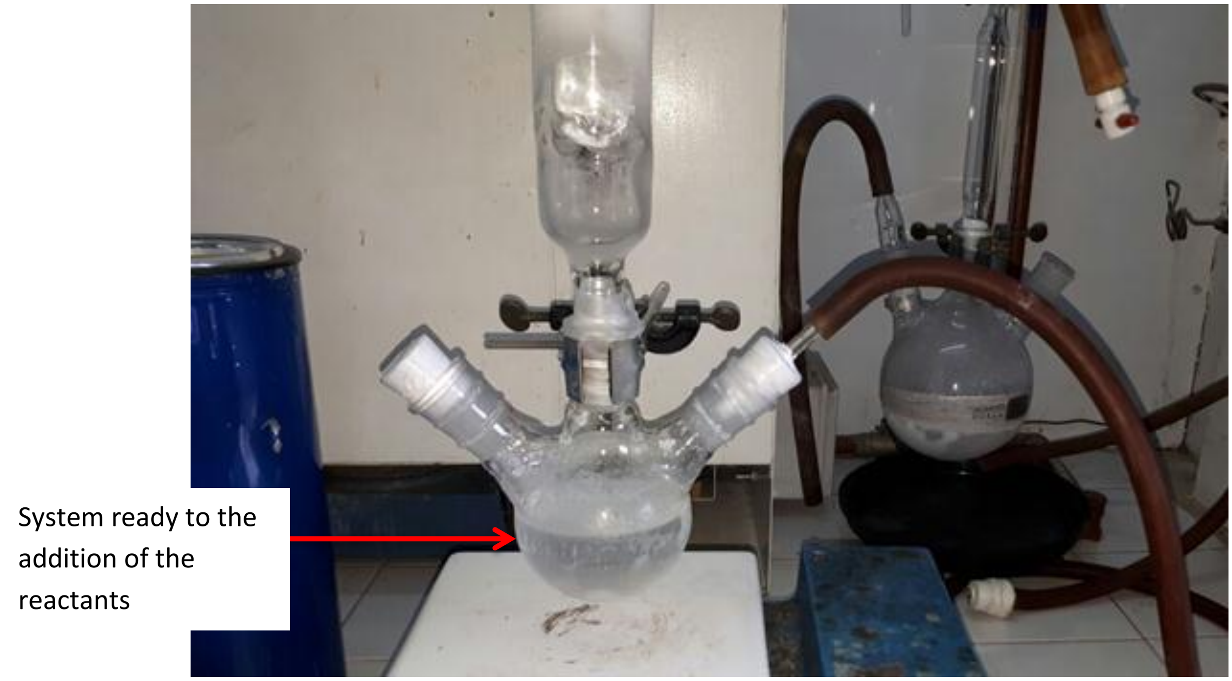

Picture **SI-6**: Experimental device for the $NH_{3(l)}$ reactions. Dark reaction.

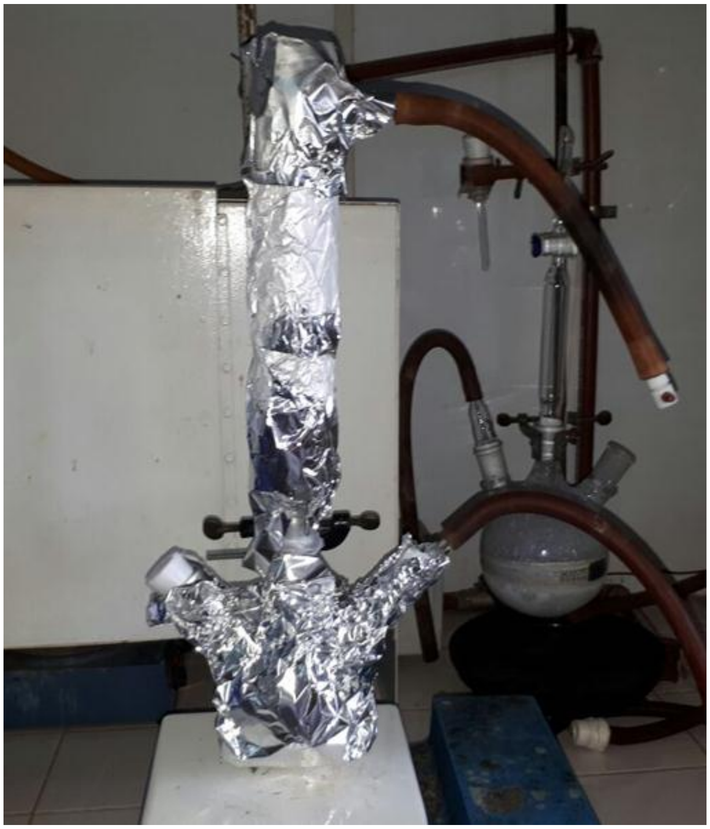

1.2 ***Figure SI-7:*** *UV-visible spectra of* ***2*****,** ***4*****⁻,** ***6*****⁻.**

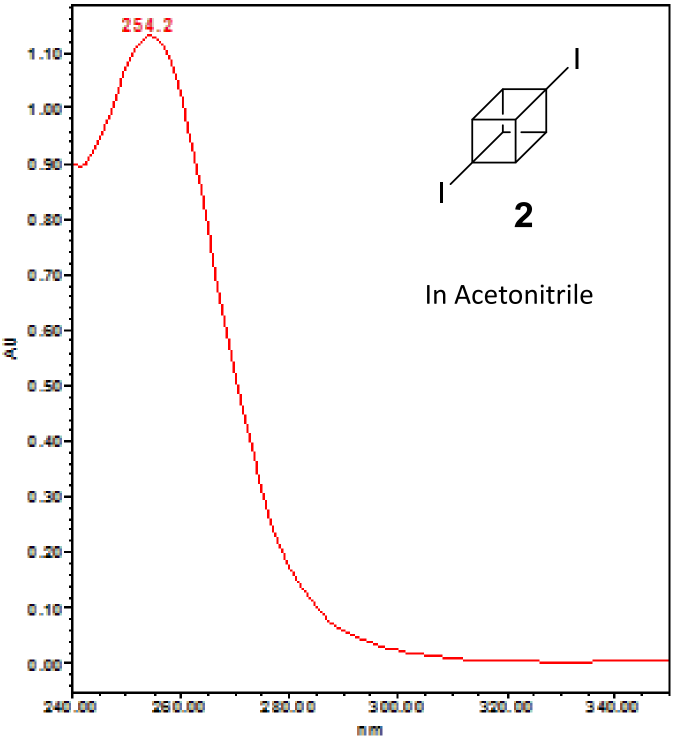

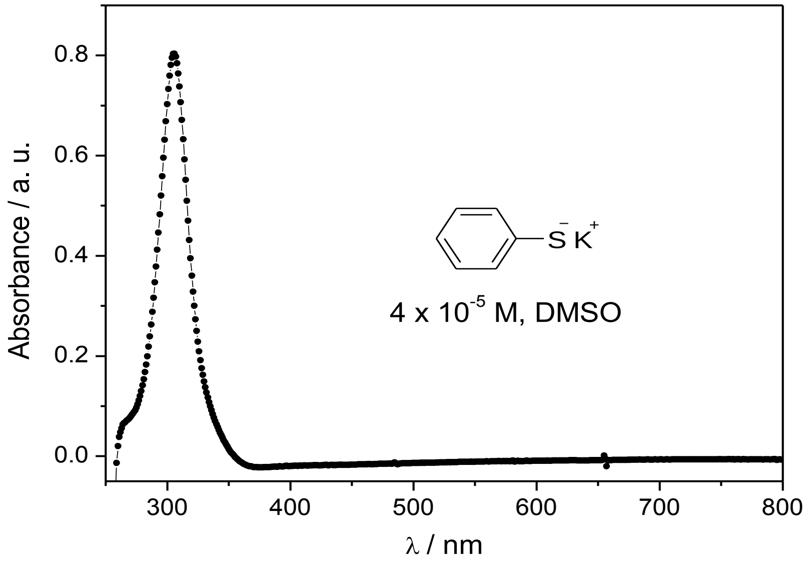

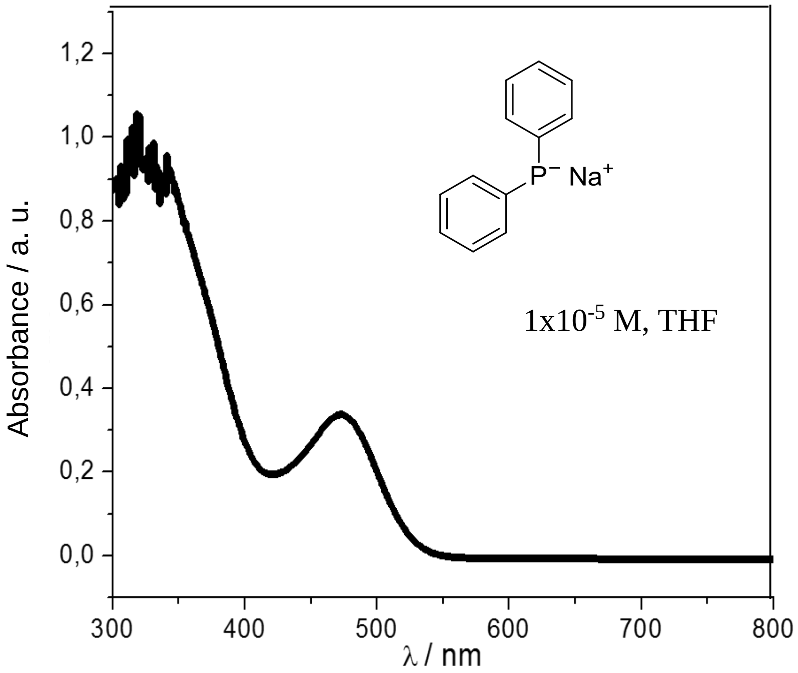
P⁻ Na⁺
1x10-5 M, THF
Absorbance / a. u.
λ / nm
1,2
1,0
0,8
0,6
0,4
0,2
0,0
300
400
500
600
700
800

Methyl 4-((4-methoxyphenyl)thio)cubane-1-carboxylate (7)

^{1}H-NMR (400 MHz, acetone-d_6)

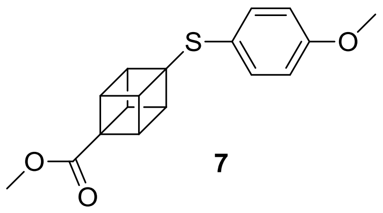

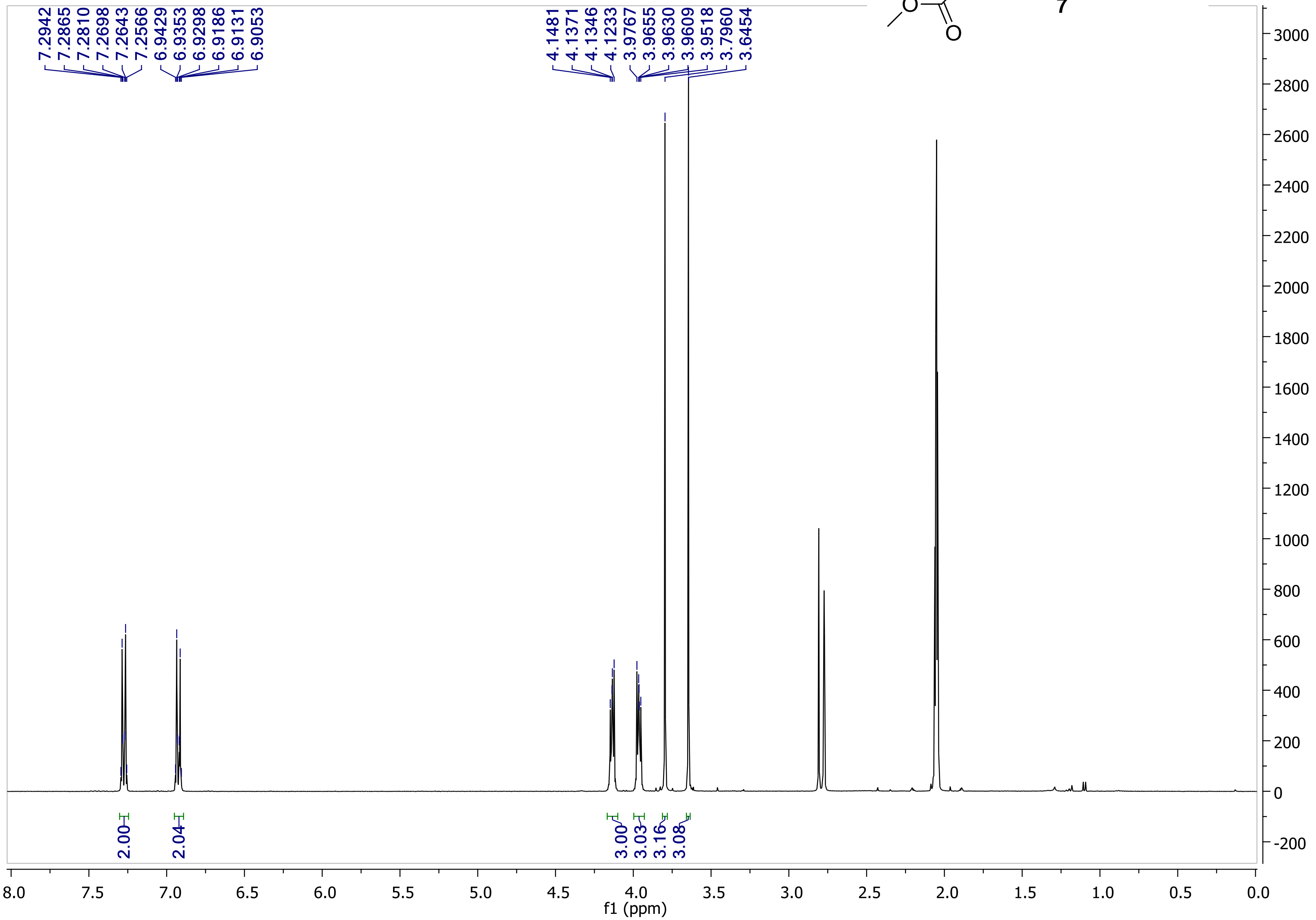

^{13}C-NMR (100 MHz, acetone-d_6)

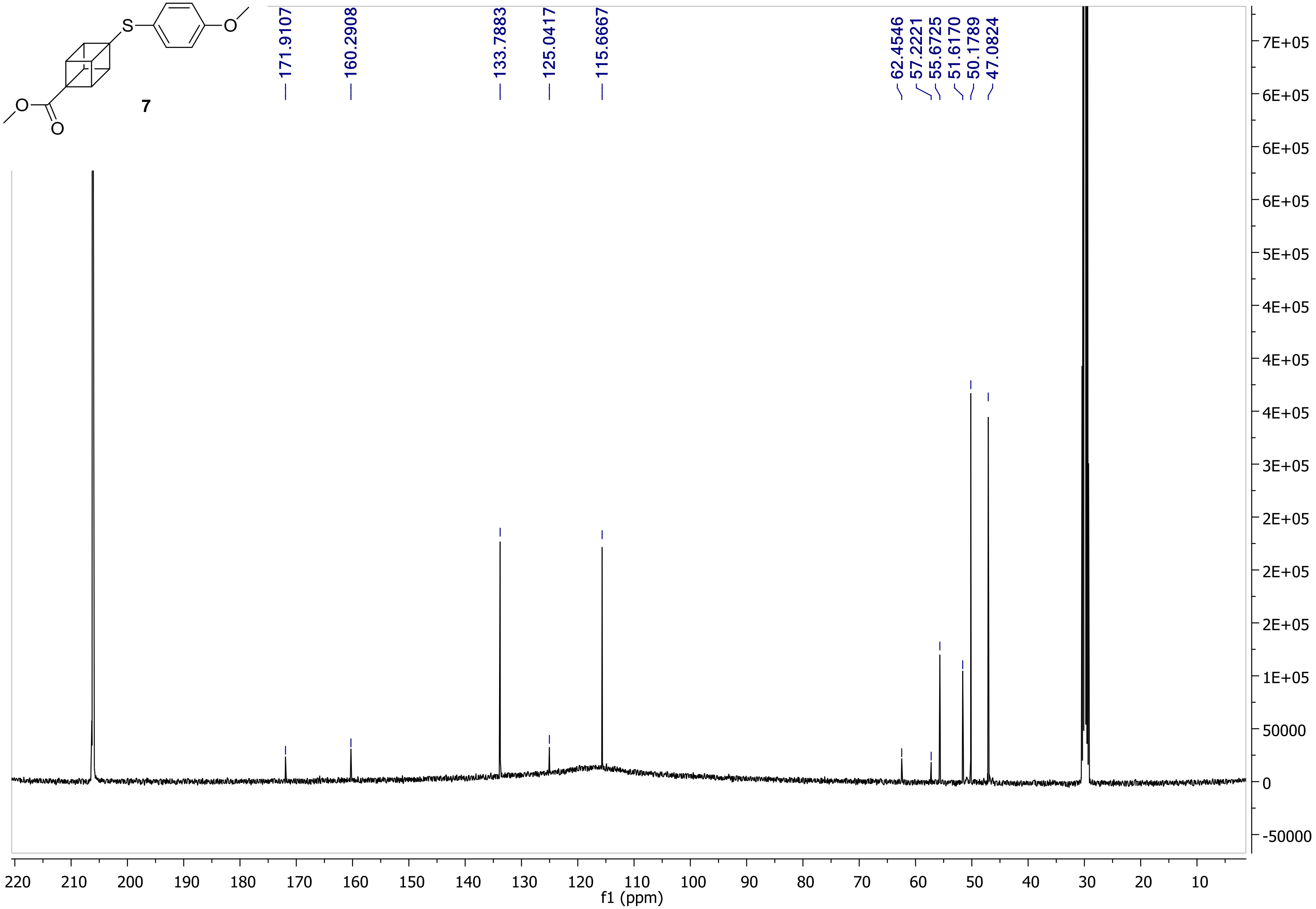

COSY (acetone-d_6)

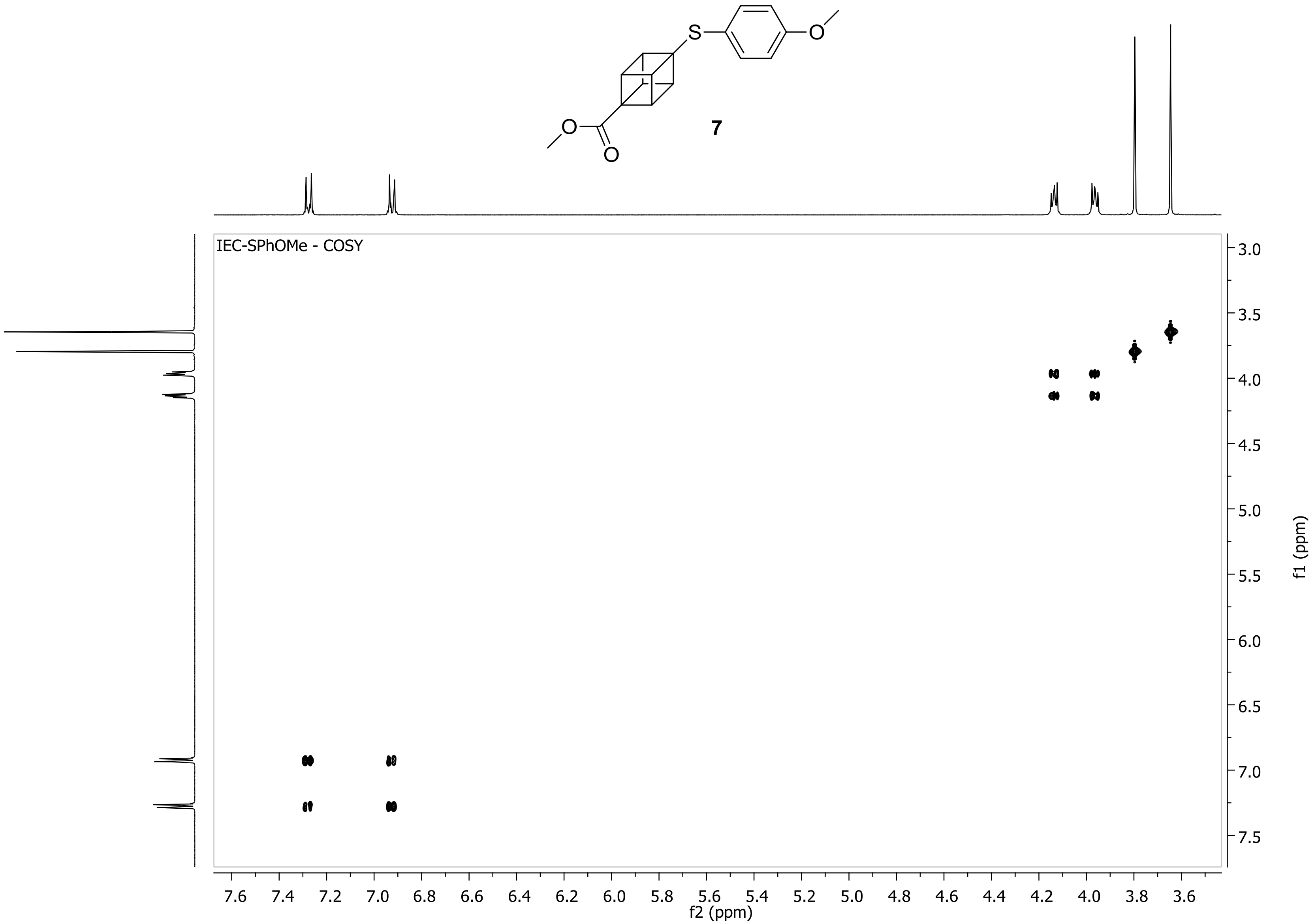

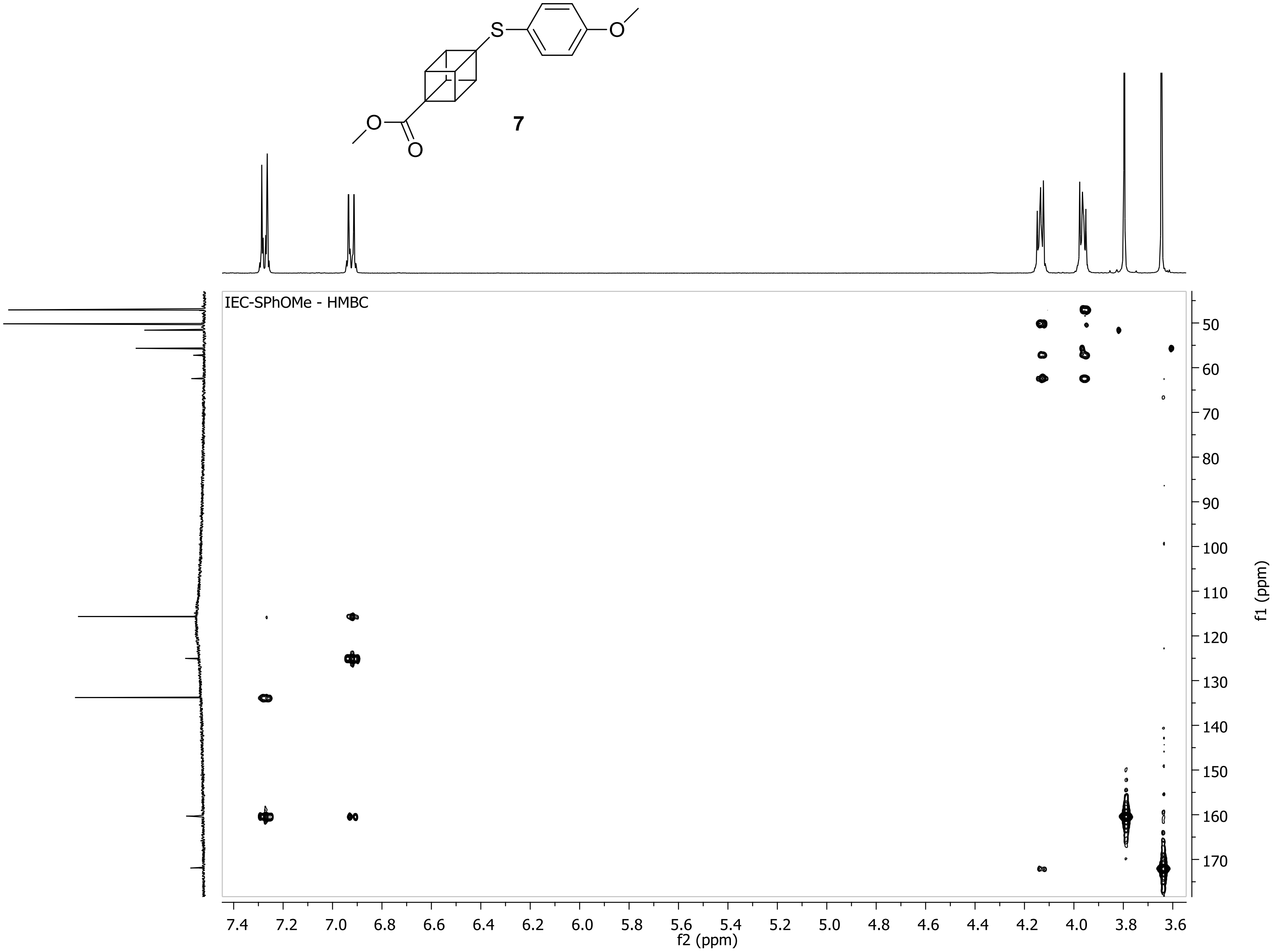
7
IEC-SPhOMe - HMBC
f2 (ppm)
f1 (ppm)

HSQC-DEPT (acetone-d_6)

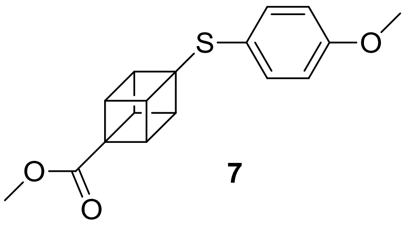

7

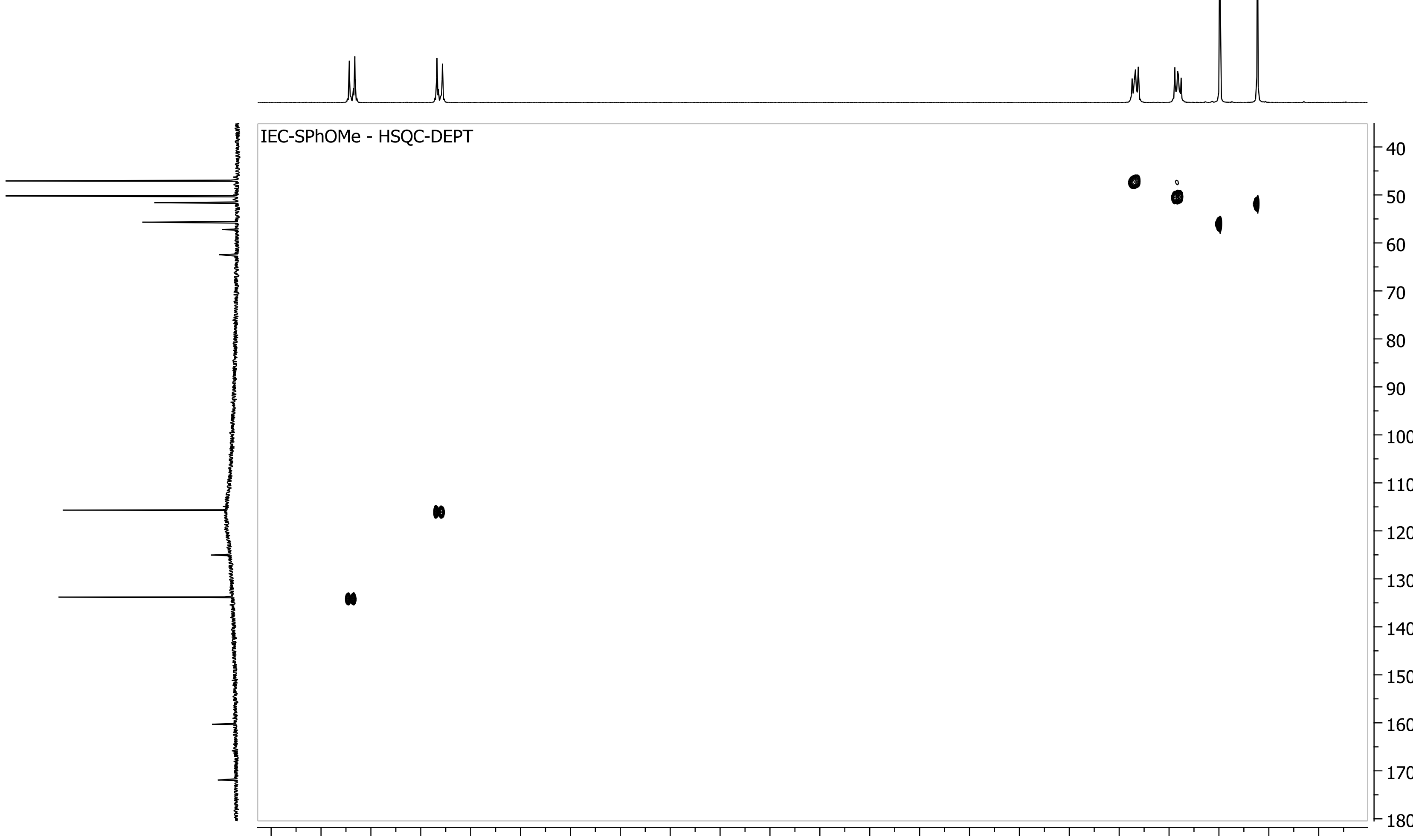

Methyl 4-(phenylthio)cubane-1-carboxylate (8)

^{1}H-NMR (400 MHz, acetone-d_6)

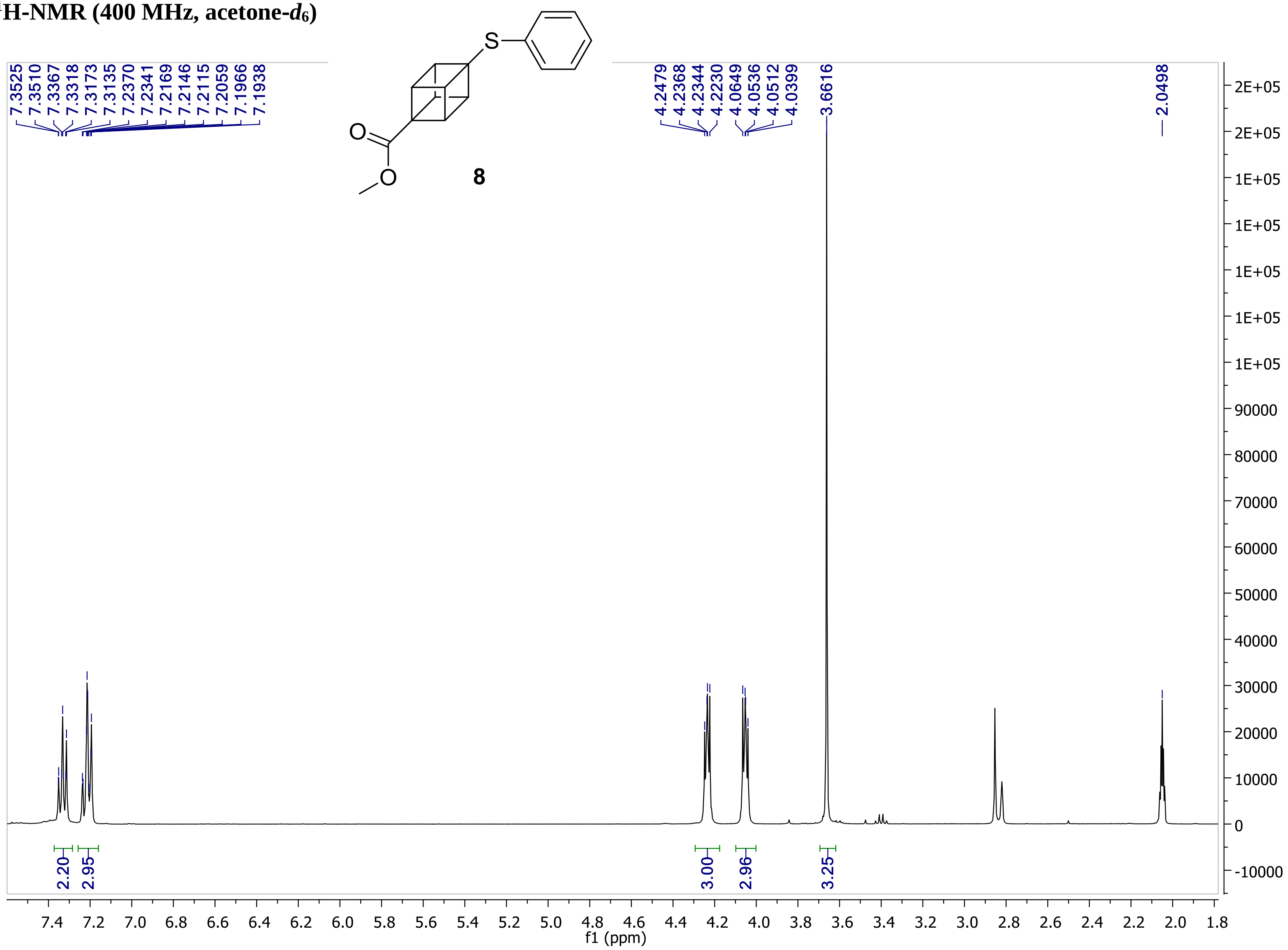

^{13}C-NMR (100 MHz, acetone-d_6)

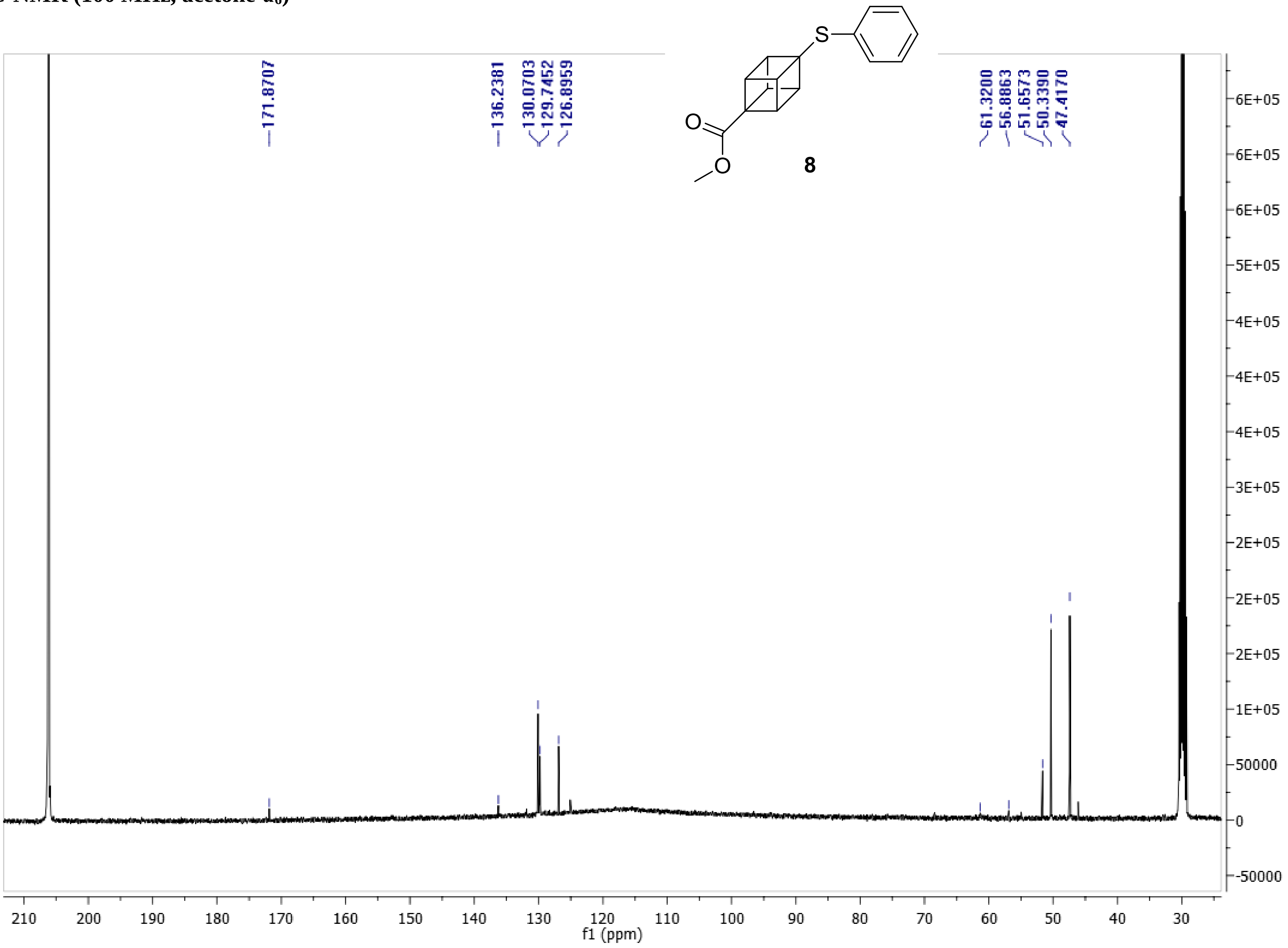

COSY (acetone-d_6)

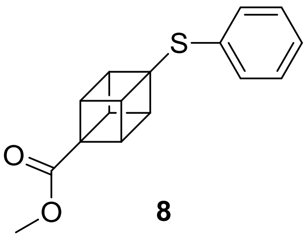

8

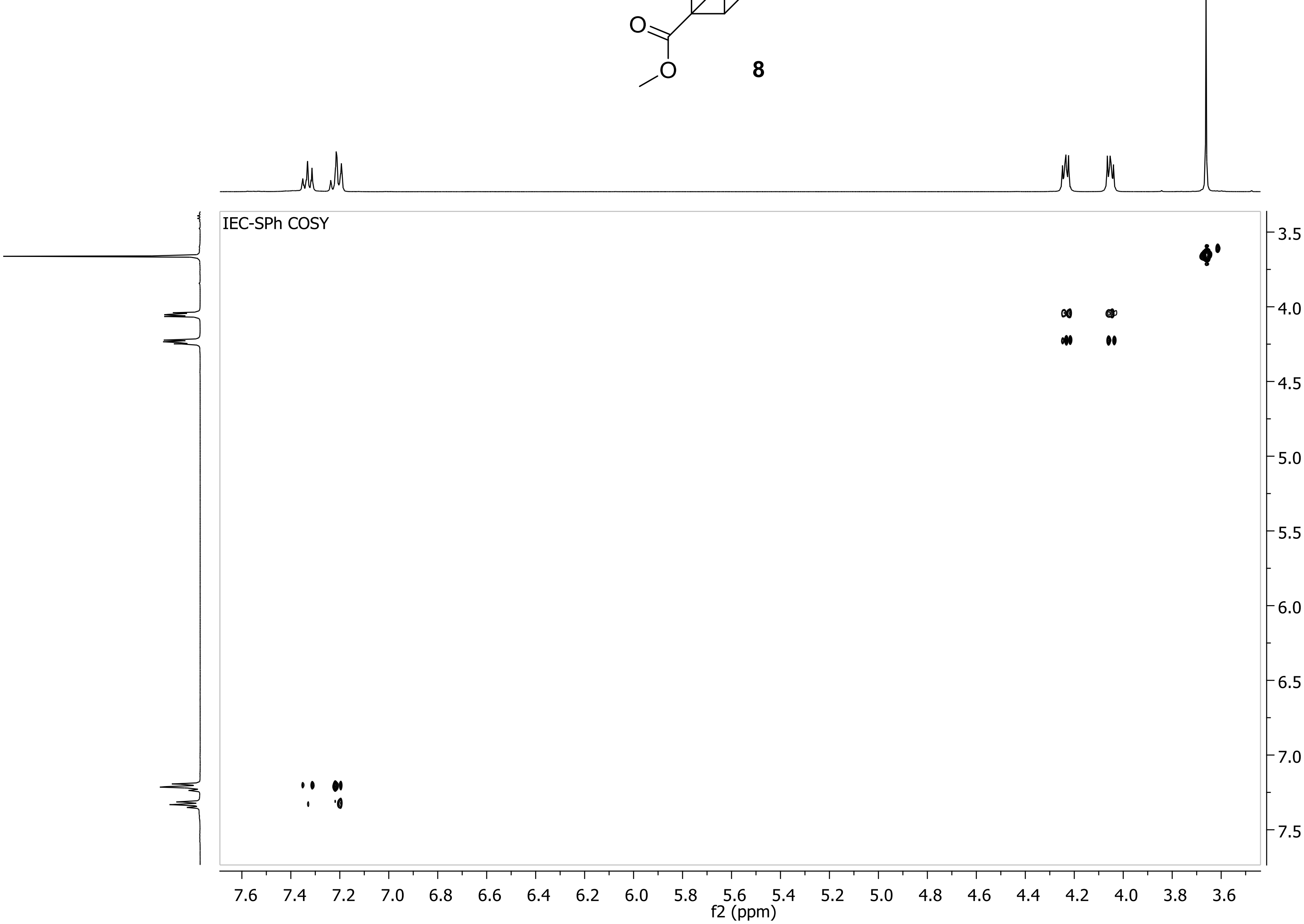

HMBC (acetone-d_6)

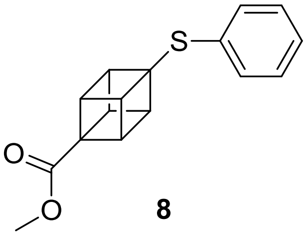

8

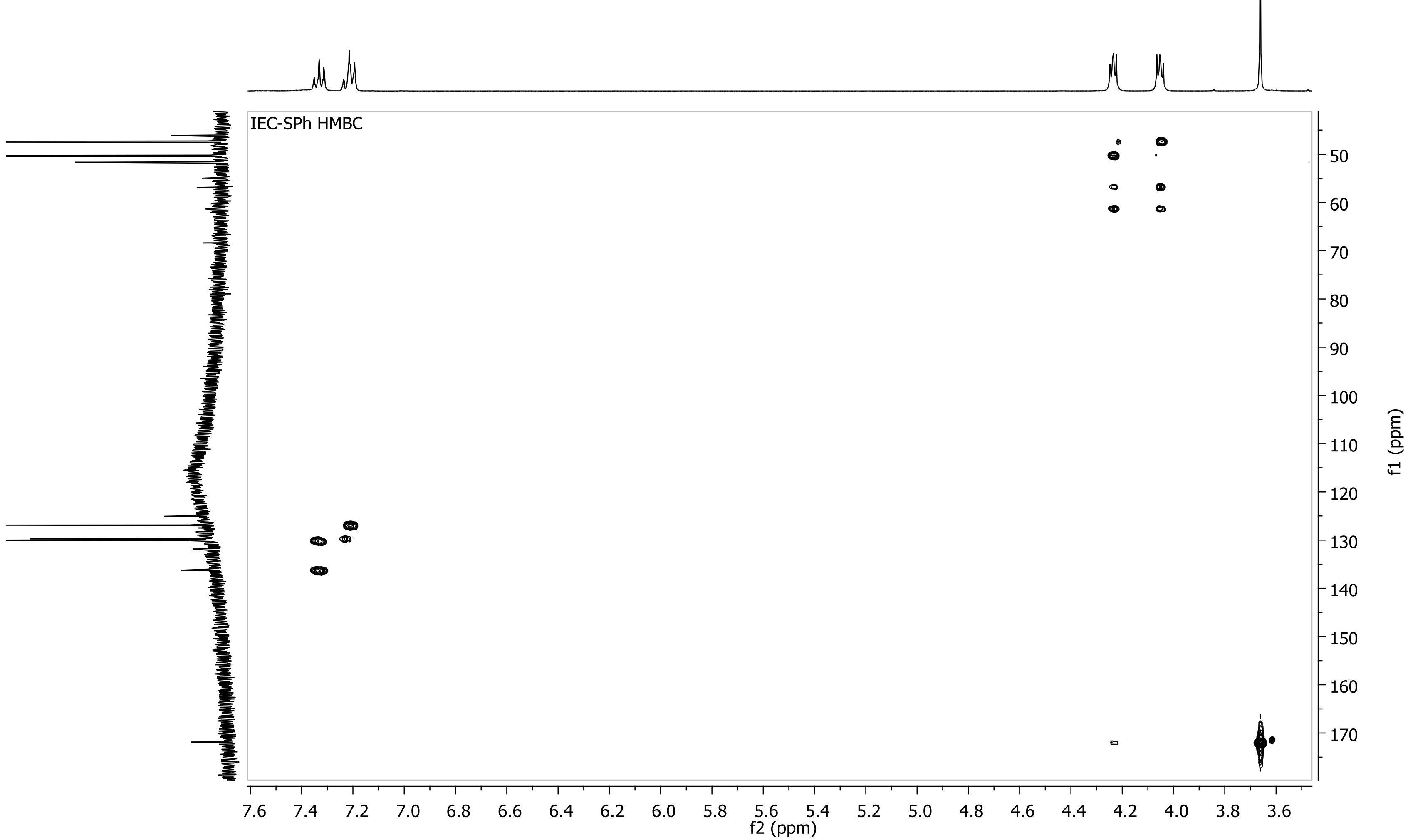

HSQC-DEPT (acetone-d_6)

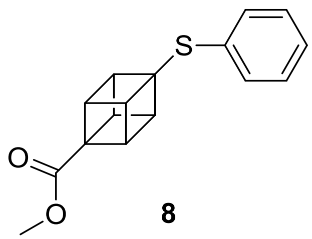

8

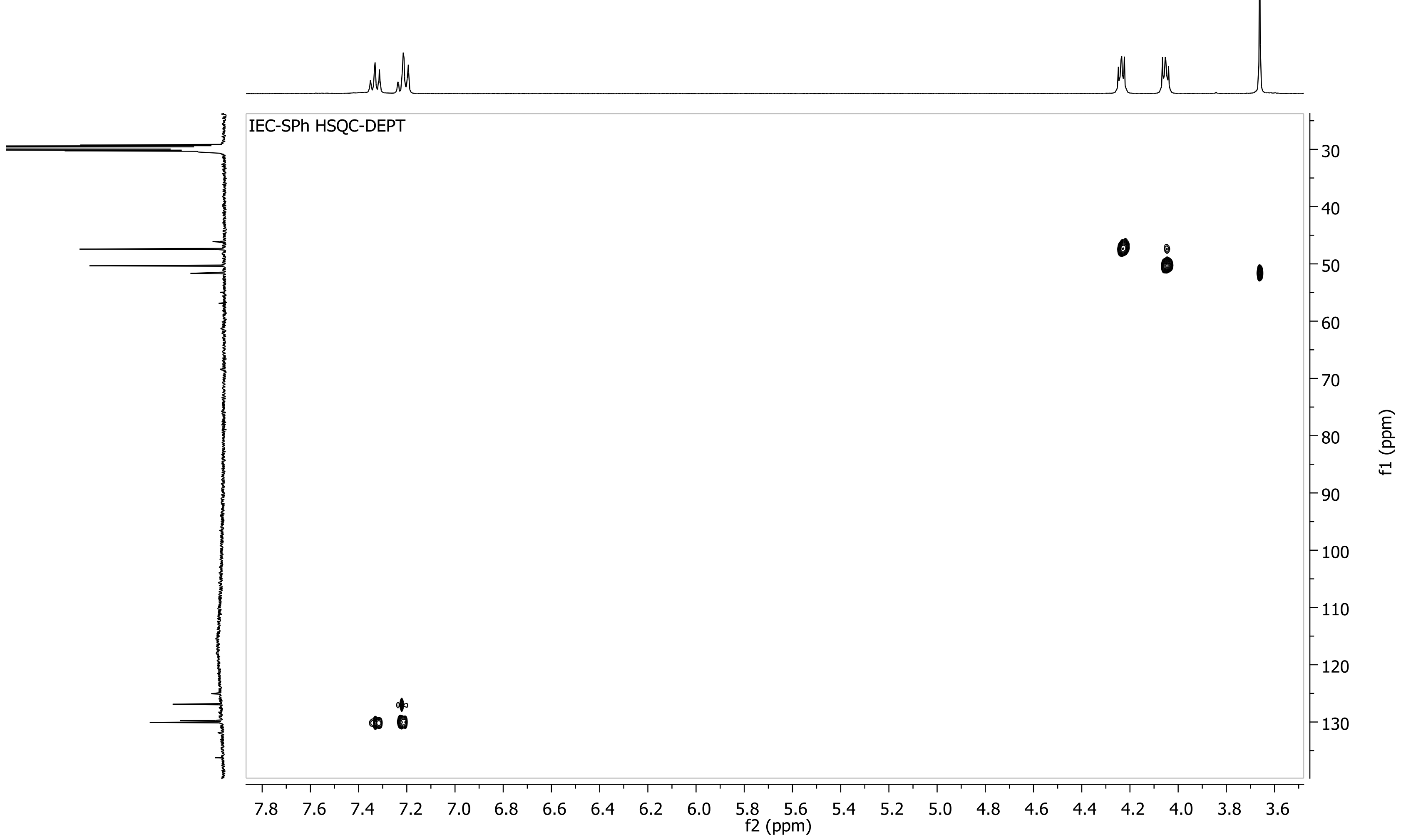

IR

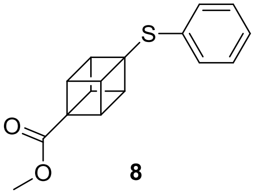

8

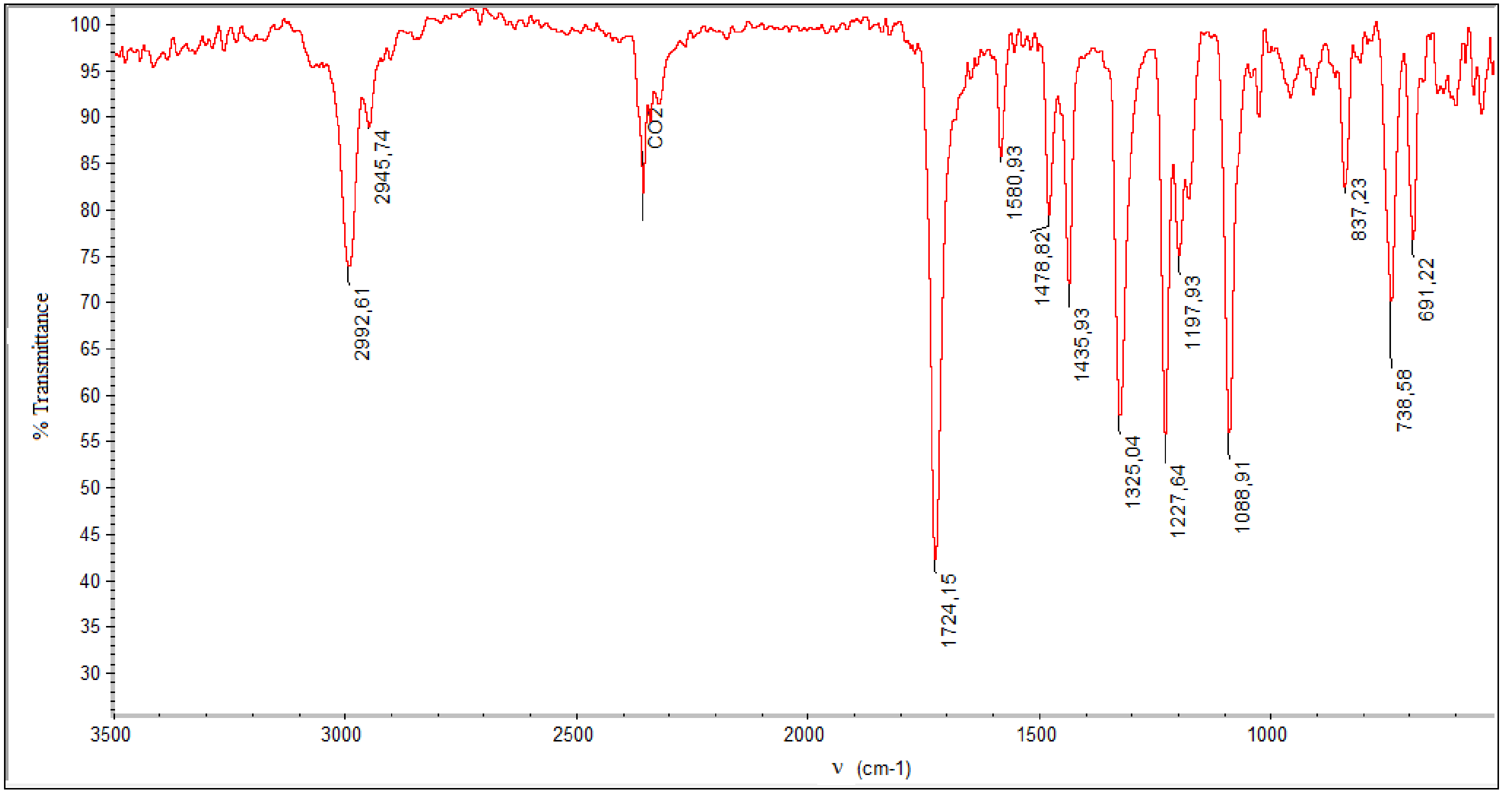

100
95
90
85
80
75
70
65
60
55
50
45
40
35
30
% Transmittance
3500
3000
2500
2000
1500
1000
ν (cm-1)
2992,61
2945,74
CO2
1724,15
1580,93
1478,82
1435,93
1325,04
1227,64
1197,93
1088,91
837,23
738,58
691,22

Methyl 4-(4-methylphenylthio)cubane-1-carboxylate (9)

^{1}H-NMR (400 MHz, acetone-d_6)

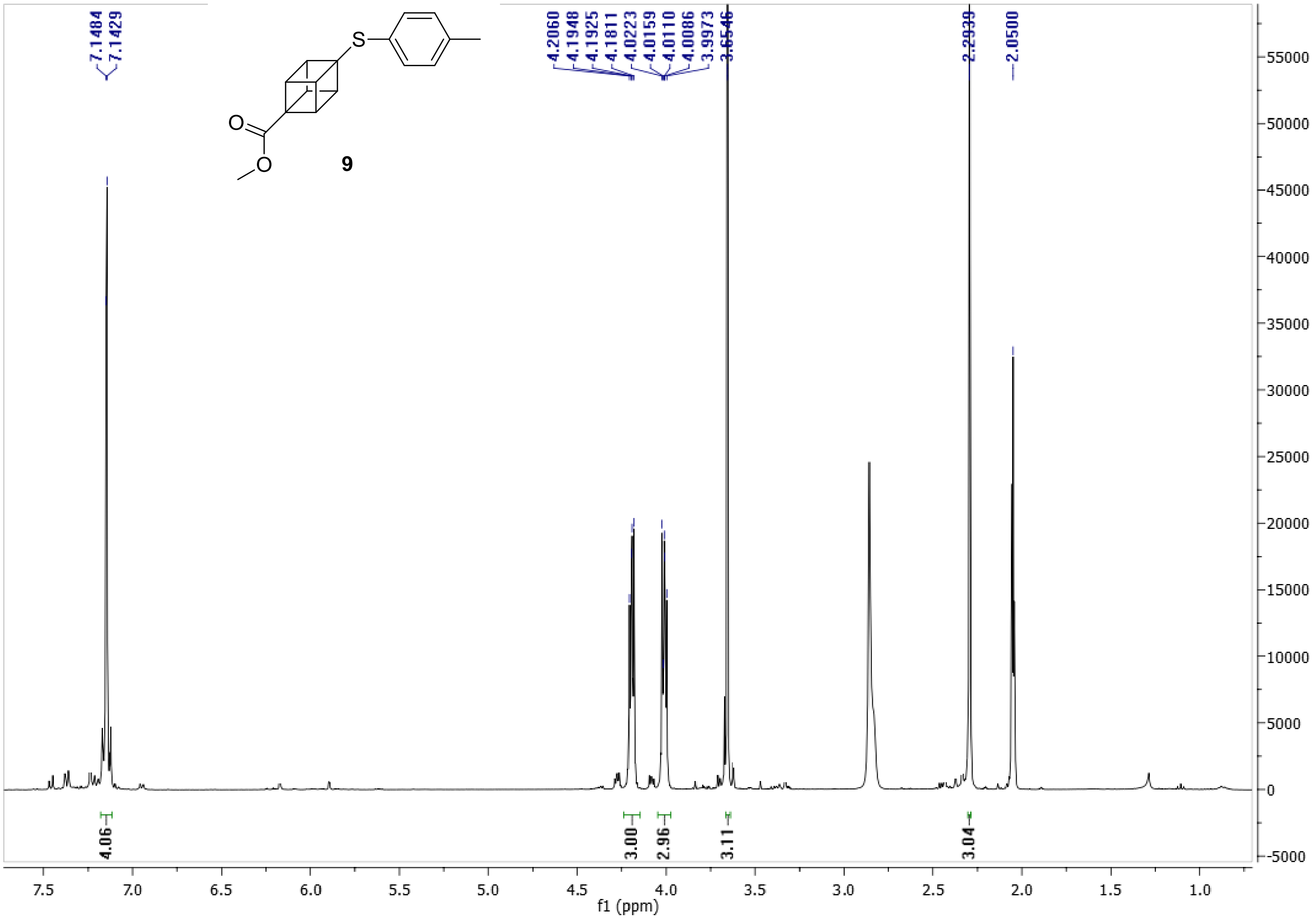

^{13}C-NMR (100 MHz, acetone-d_6)

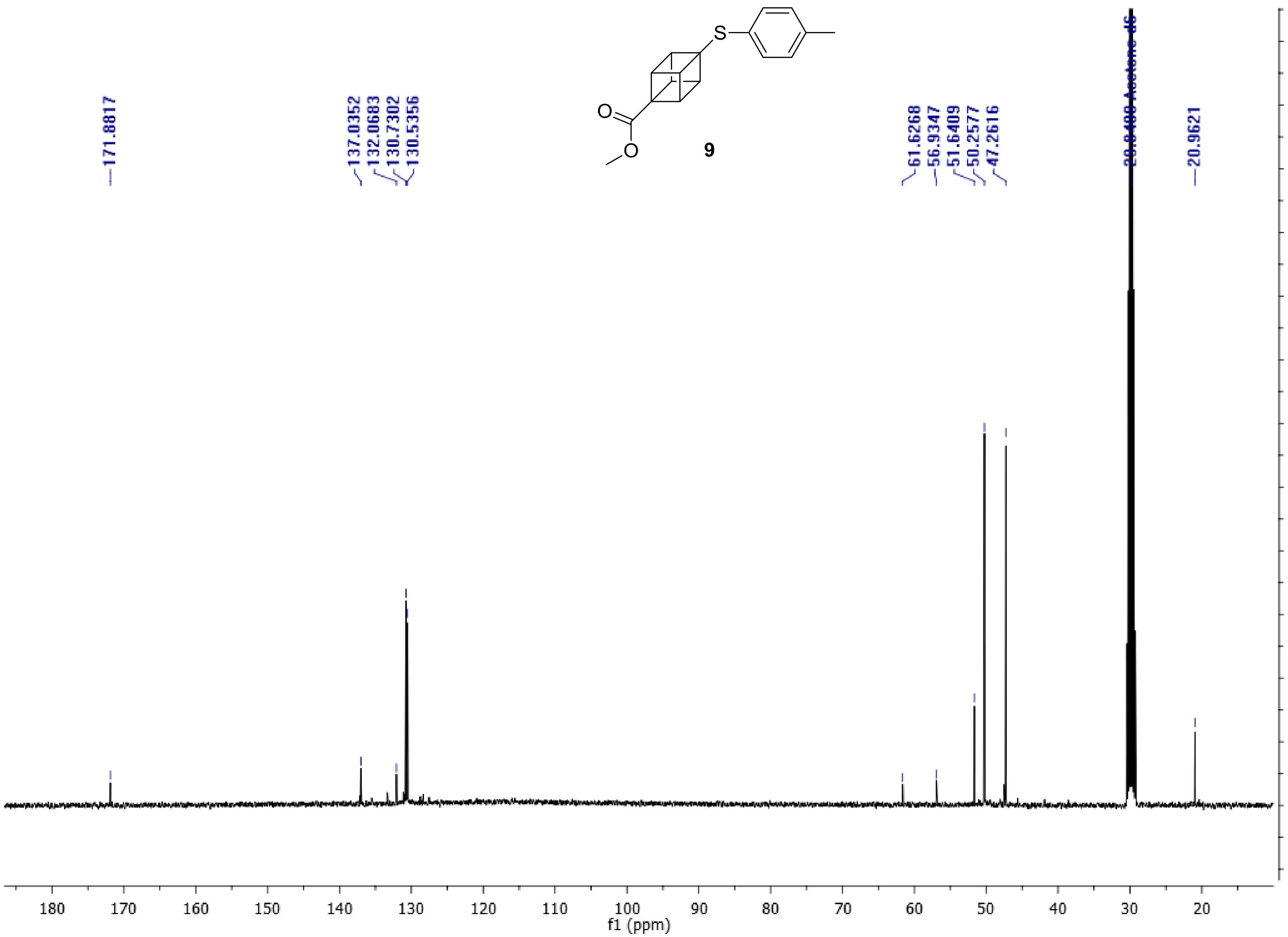

4-(diphenylphosphoryl)cubane-1-carboxylic acid (9a)

^{1}H-NMR (400 MHz, dimethyl sulfoxide-d_6)

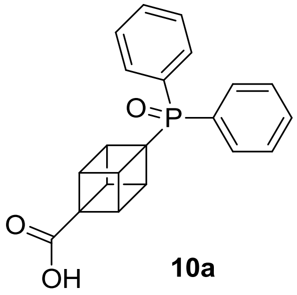

10a

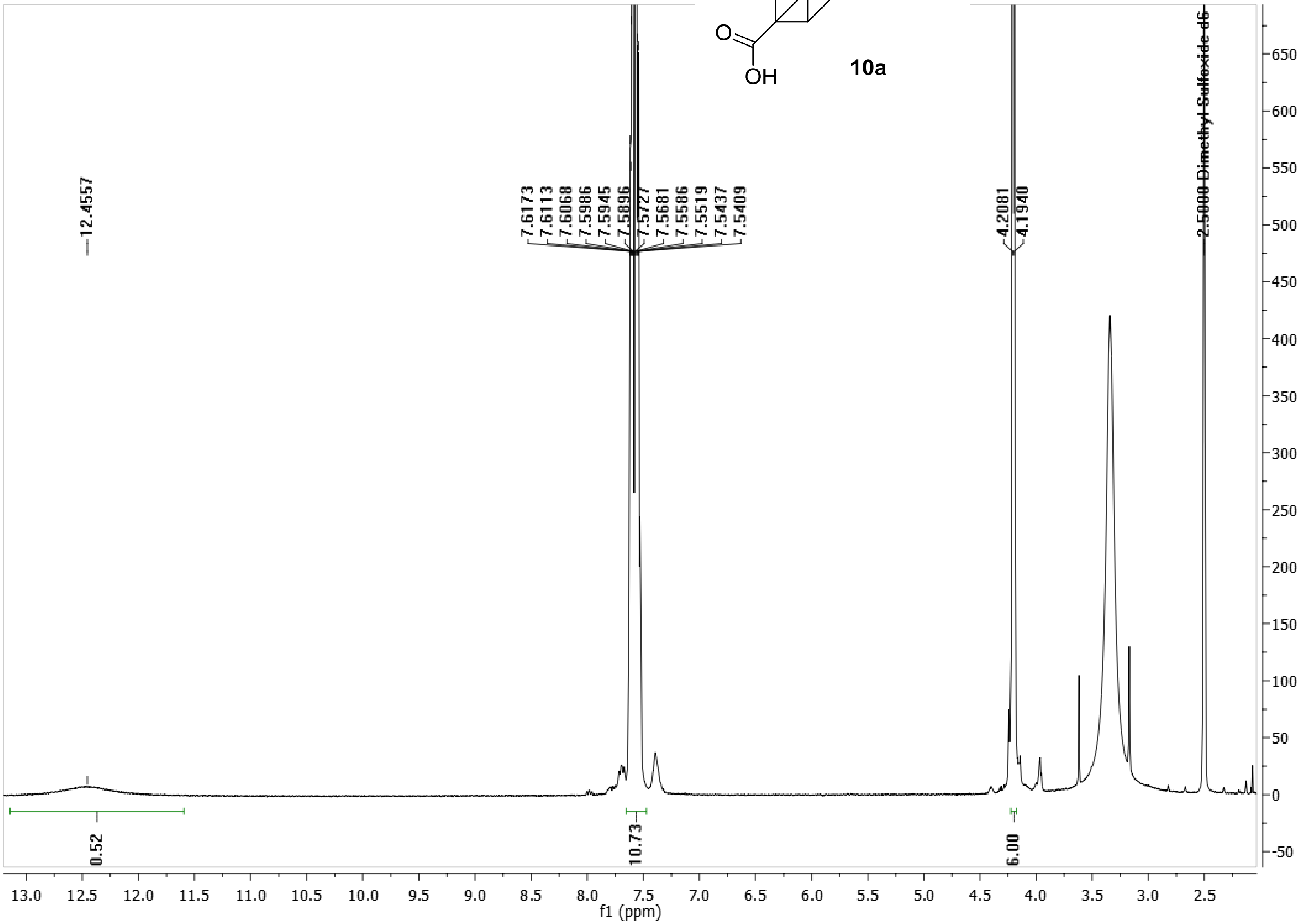

^{13}C-NMR (100 MHz, dimethyl sulfoxide-d_6)

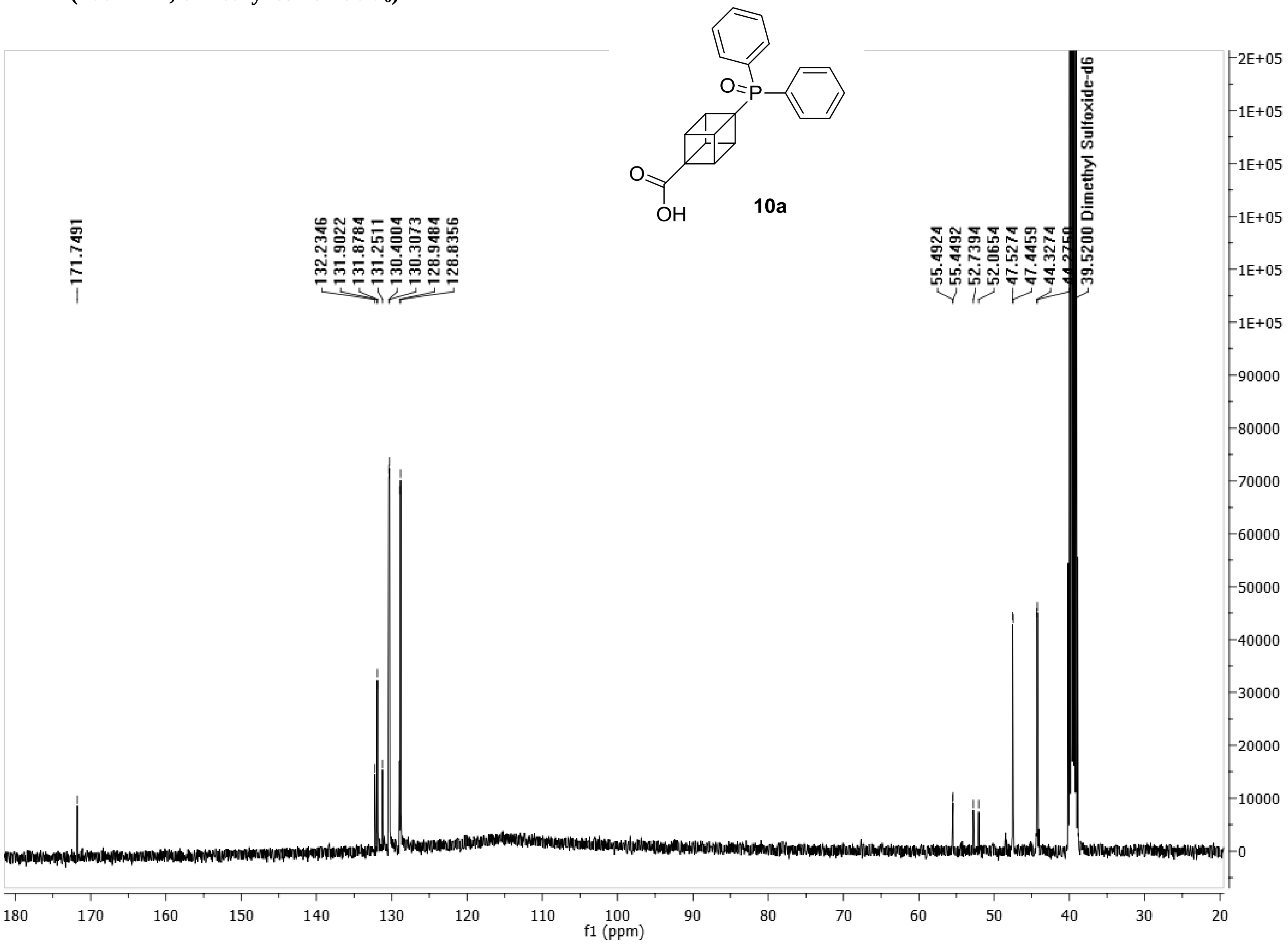

^{13}C-NMR (100 MHz, dimethyl sulfoxide-d_6)

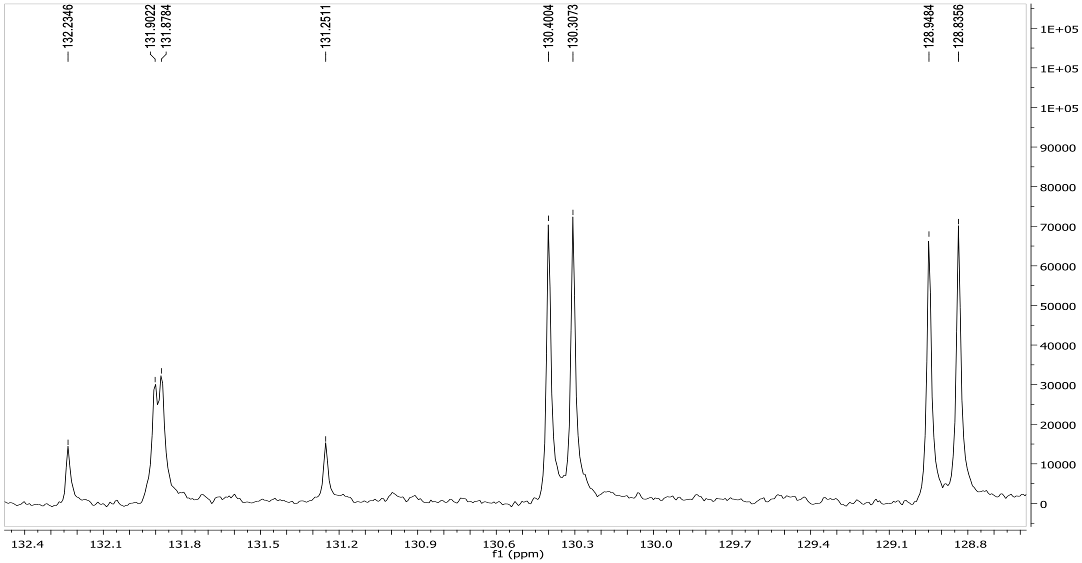

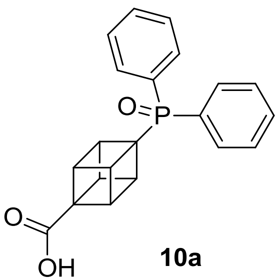

10a

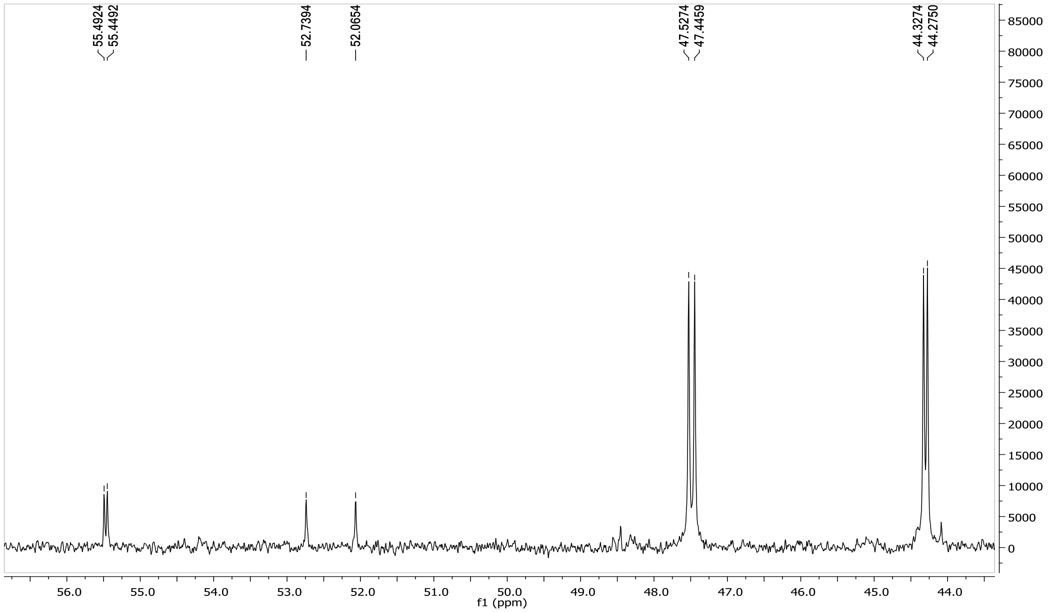

1,4-bis((4-methoxyphenyl)thio)cubane(12a)

^{1}H-NMR (400 MHz, chloroform-*d*)

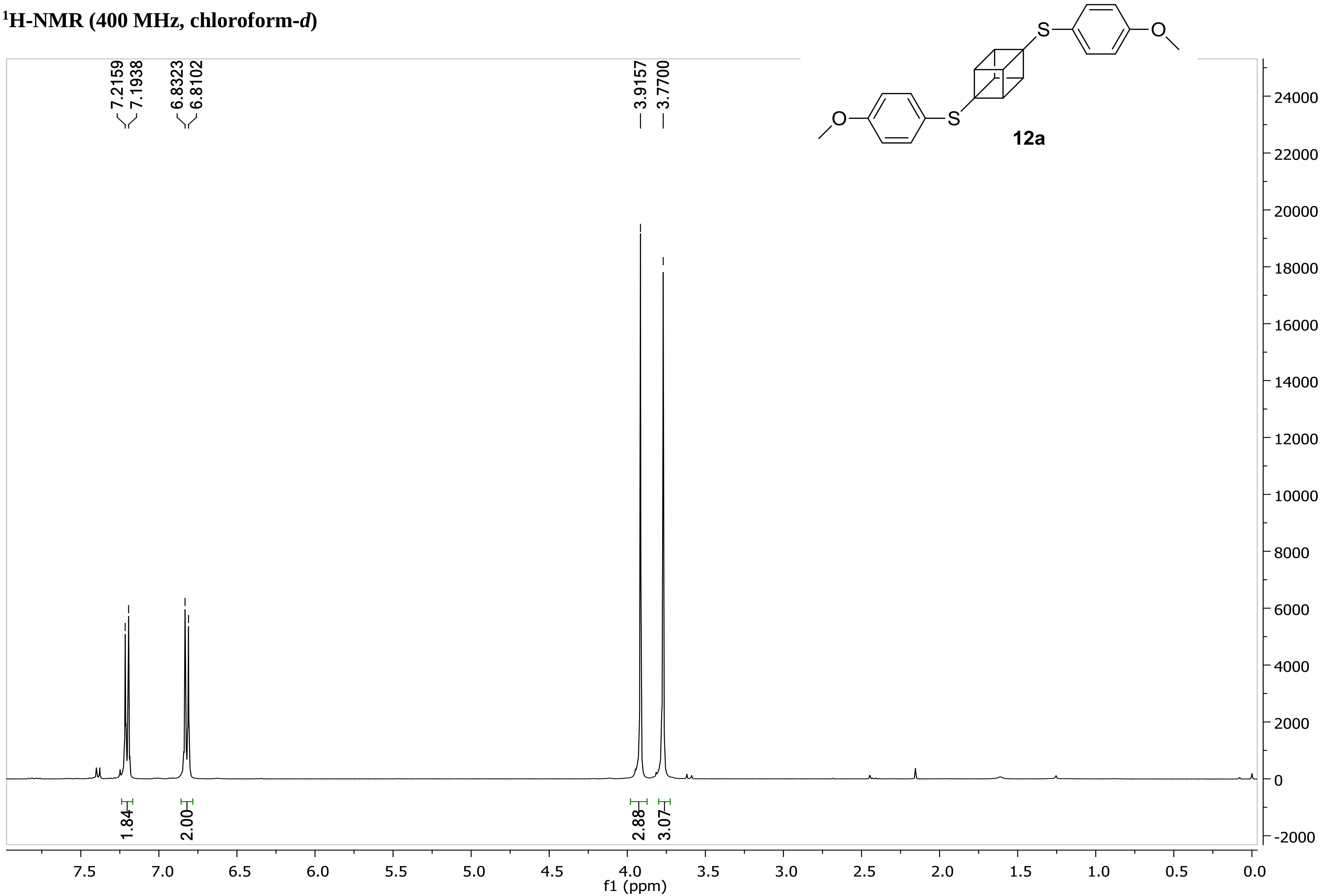

^{13}C-NMR (100 Hz, chloroform-*d*)

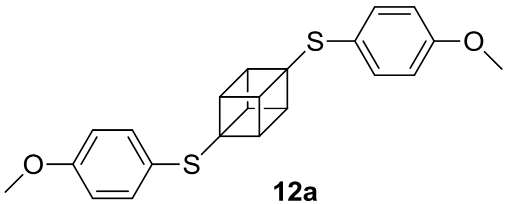

12a

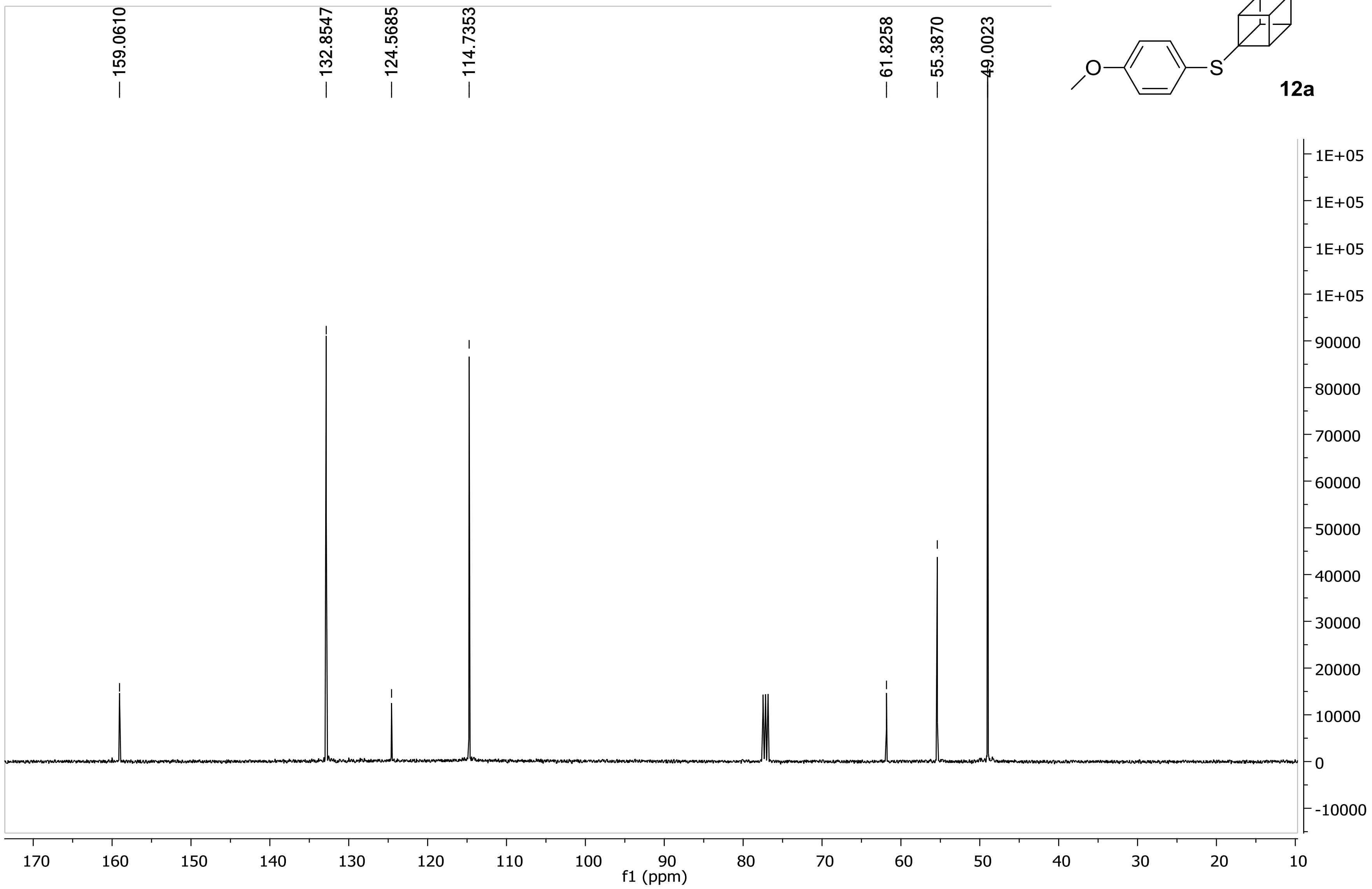

1,4-bis(phenylthio)cubane (12b)

^{1}H-NMR (400 MHzchloroform-*d*)

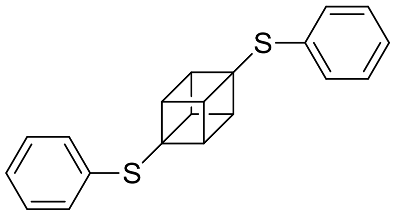

12b

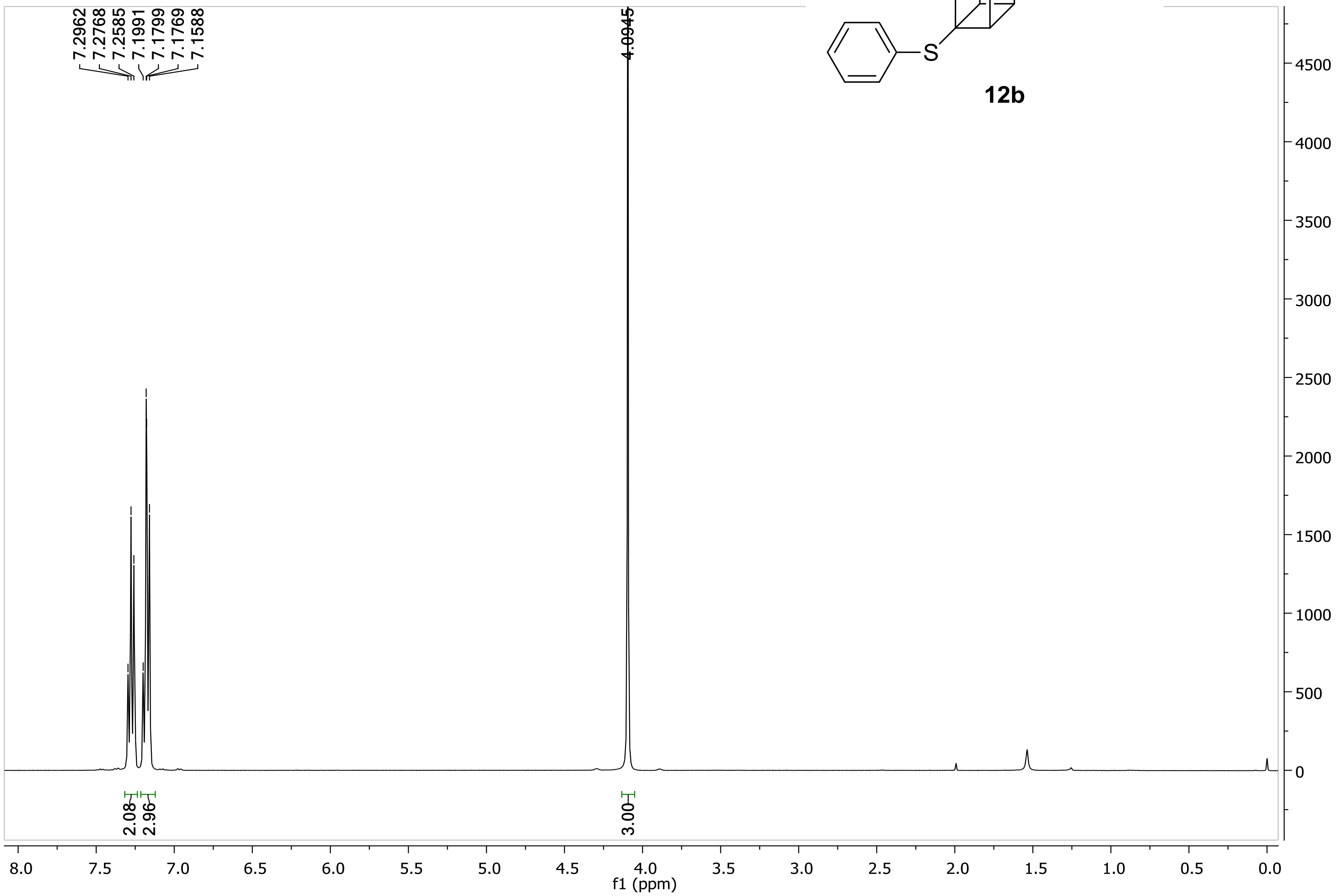

^{13}C-NMR (100 MHz, chloroform-*d*)

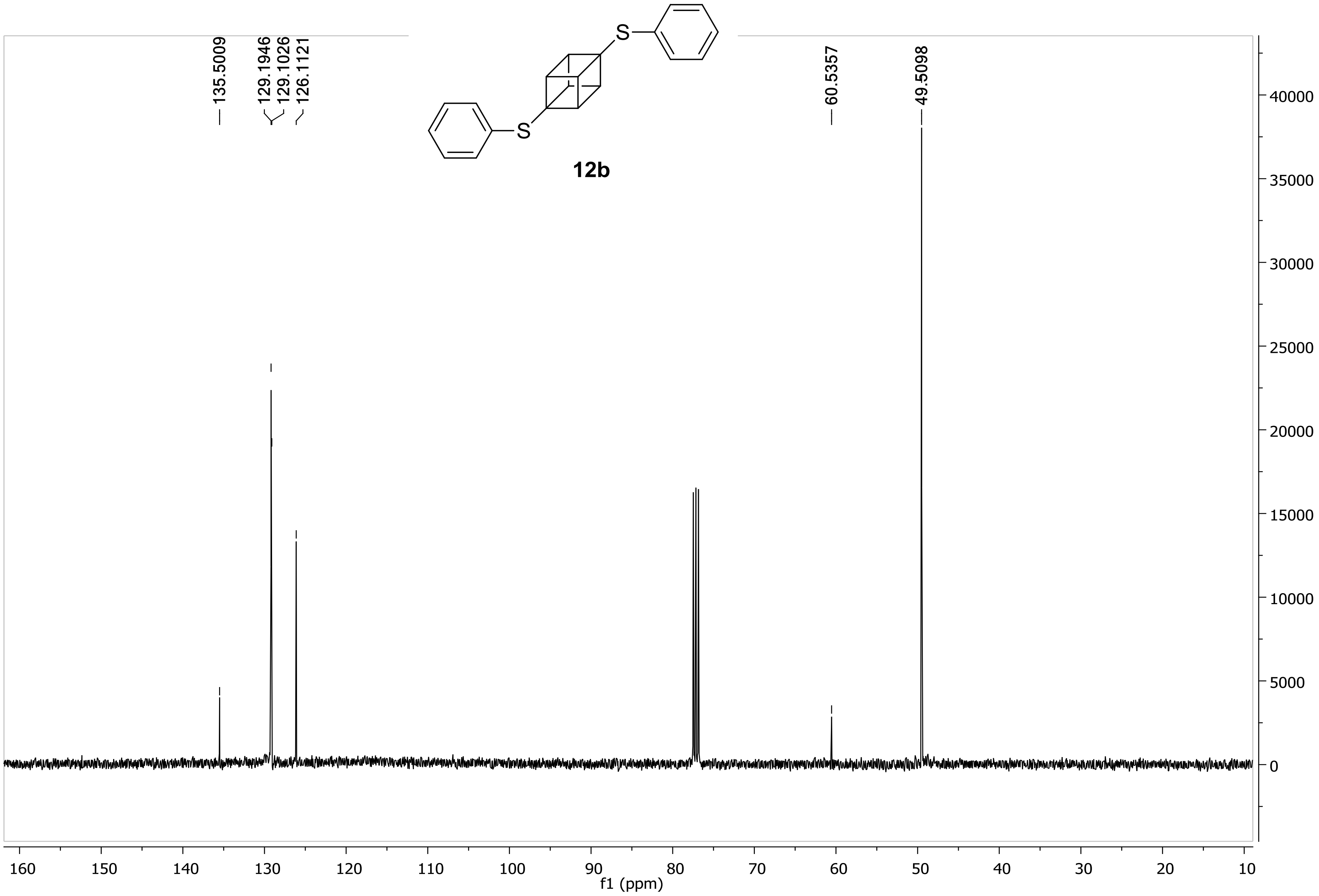

IR

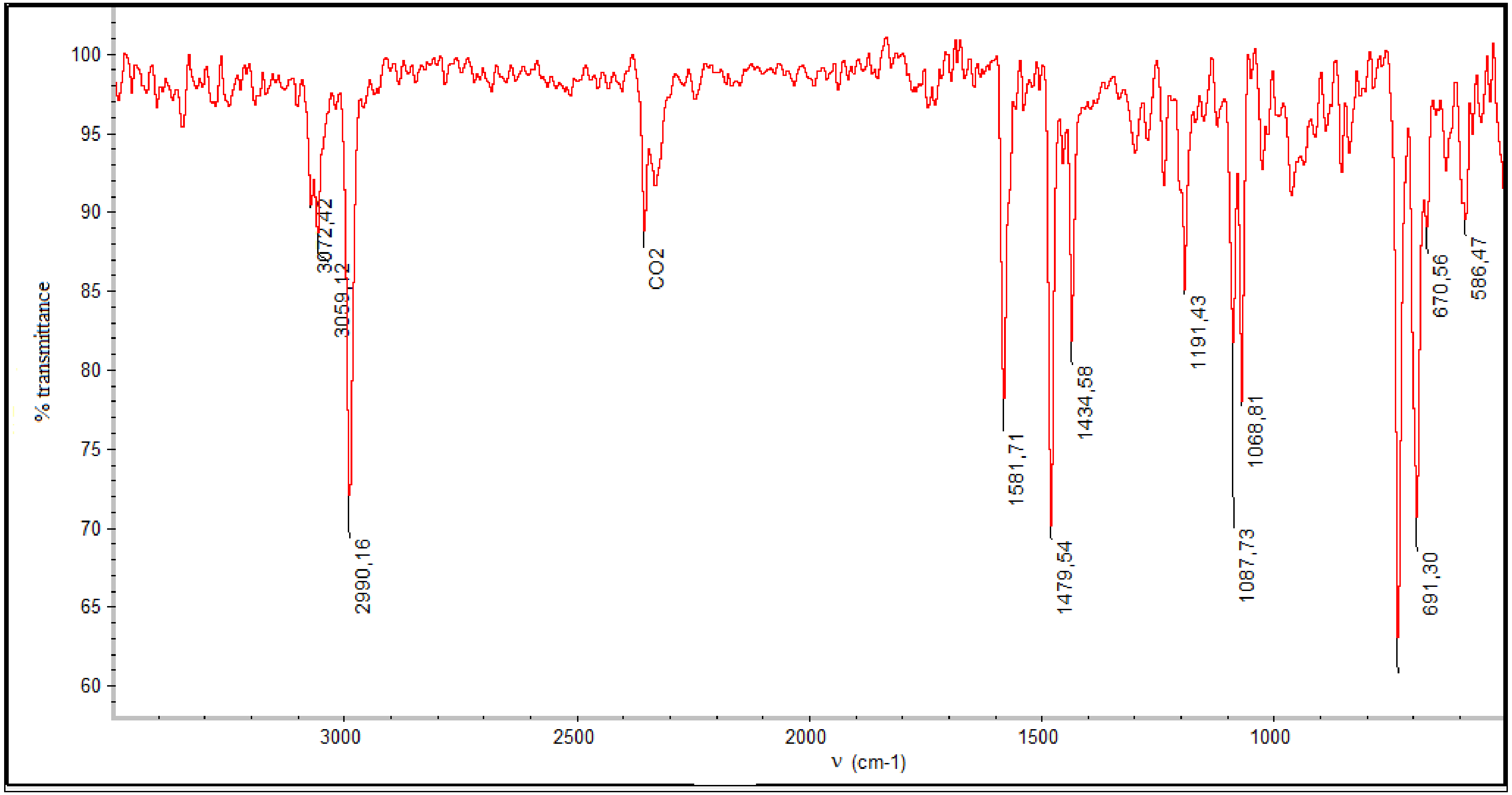

100
95
90
85
80
75
70
65
60
% transmittance
3000
2500
2000
1500
1000
ν (cm-1)
3072,42
3059,12
2990,16
CO2
1581,71
1479,54
1434,58
1191,43
1087,73
1068,81
691,30
670,56
586,47

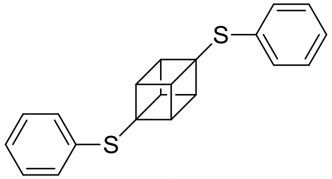

S
S

12b

(4-iodocuban-1-yl)(4-methoxyphenyl)sulfane (13a)

^{1}H-NMR (400 MHz, chloroform-*d*)

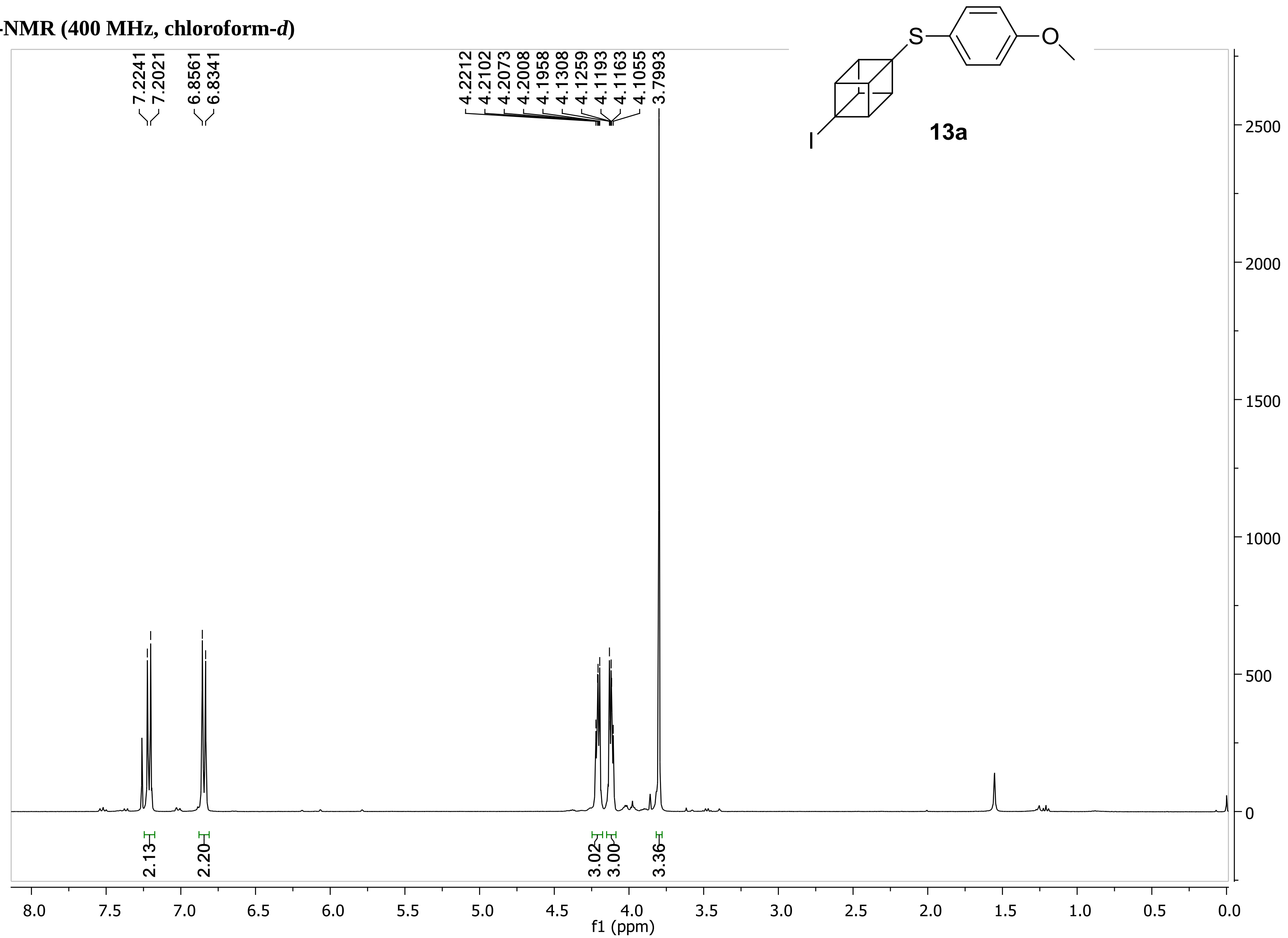

^{13}C-NMR (100 MHz, chloroform-*d*)

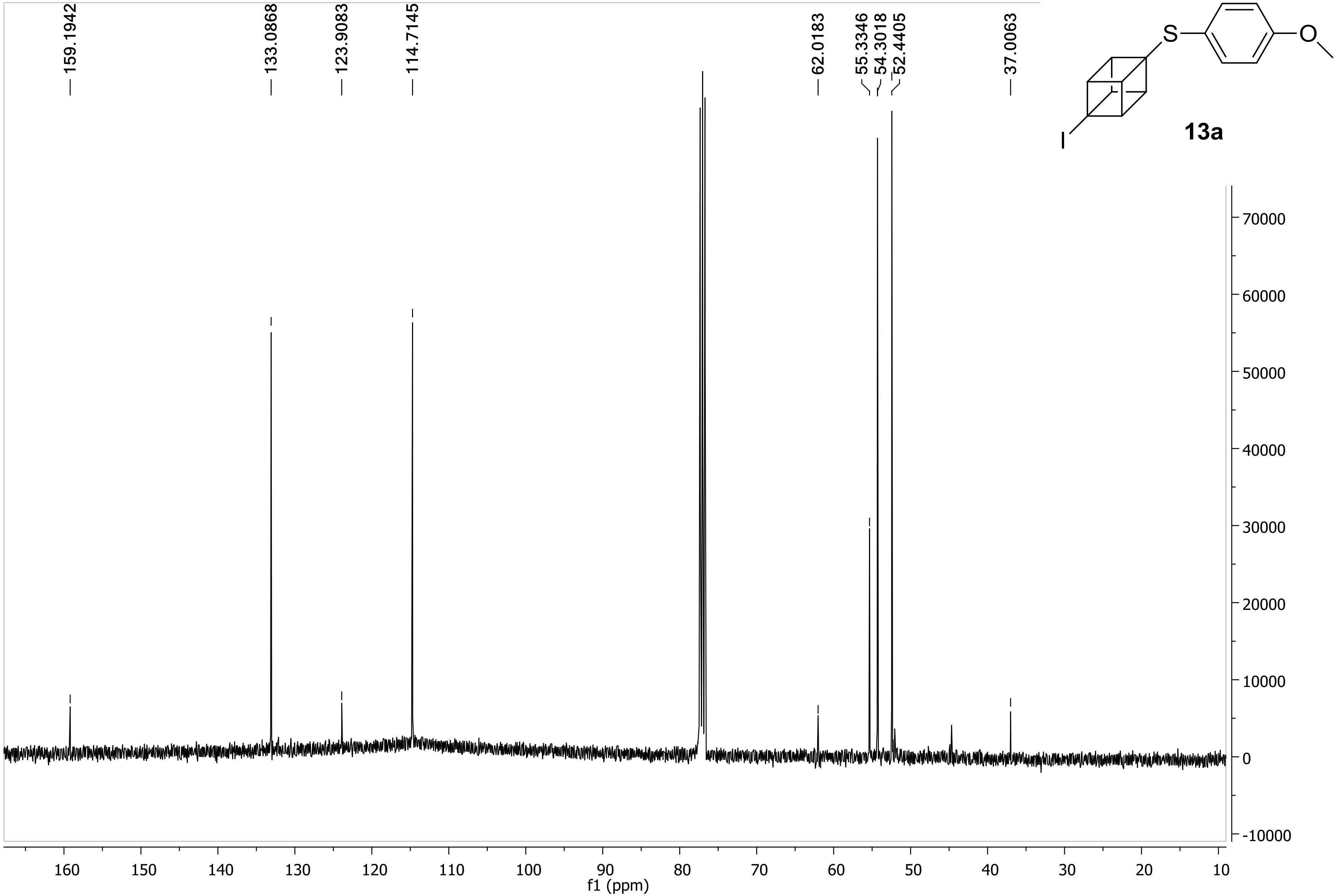

(4-iodocuban-1-yl)(phenyl)sulfane (13b)

^{1}H-NMR (400 MHz, chloroform-*d*)

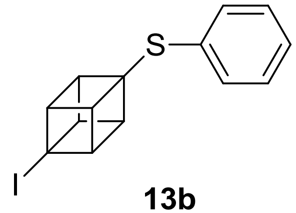

13b

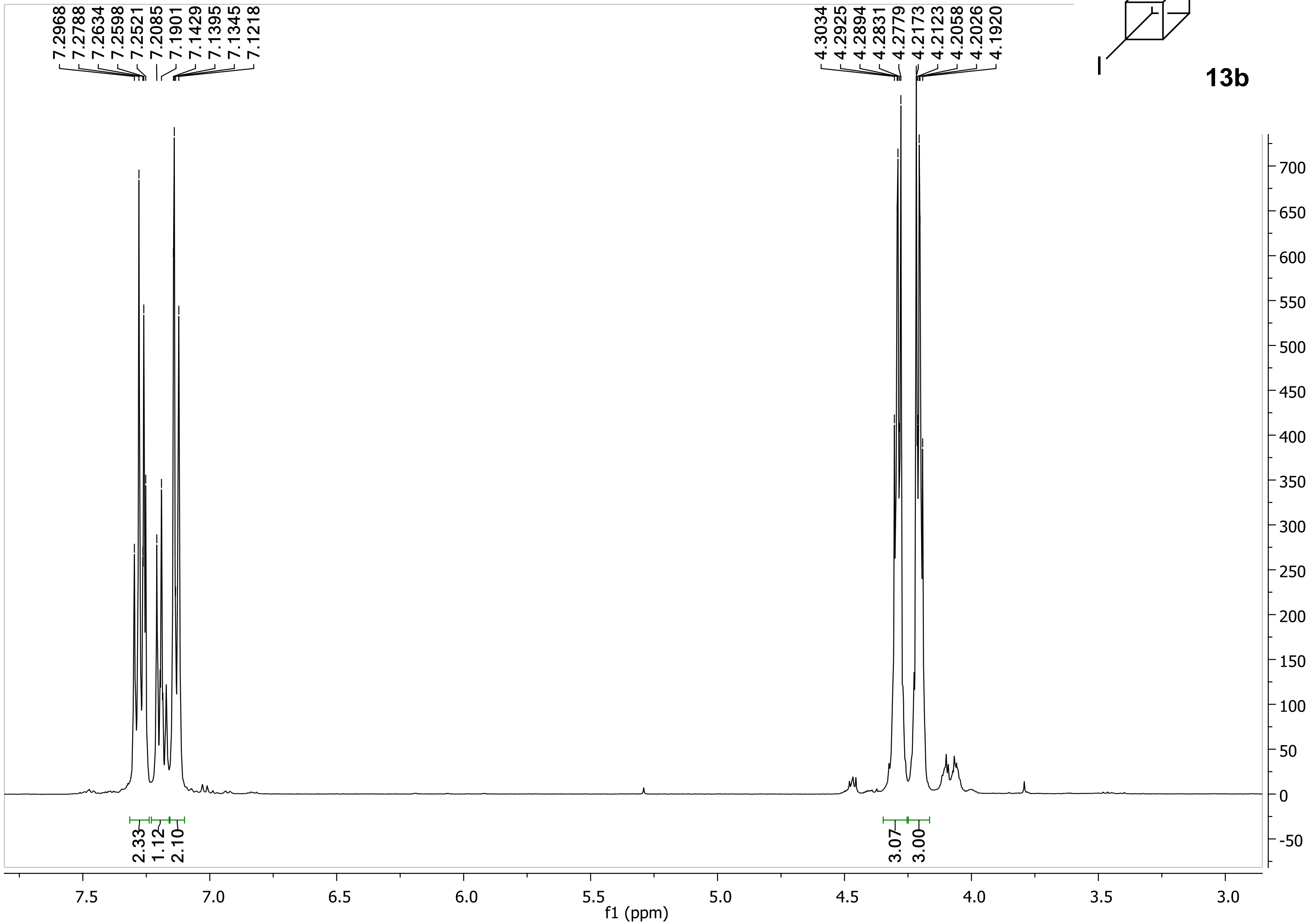

^{13}C-NMR (100 MHz, chloroform-*d*)

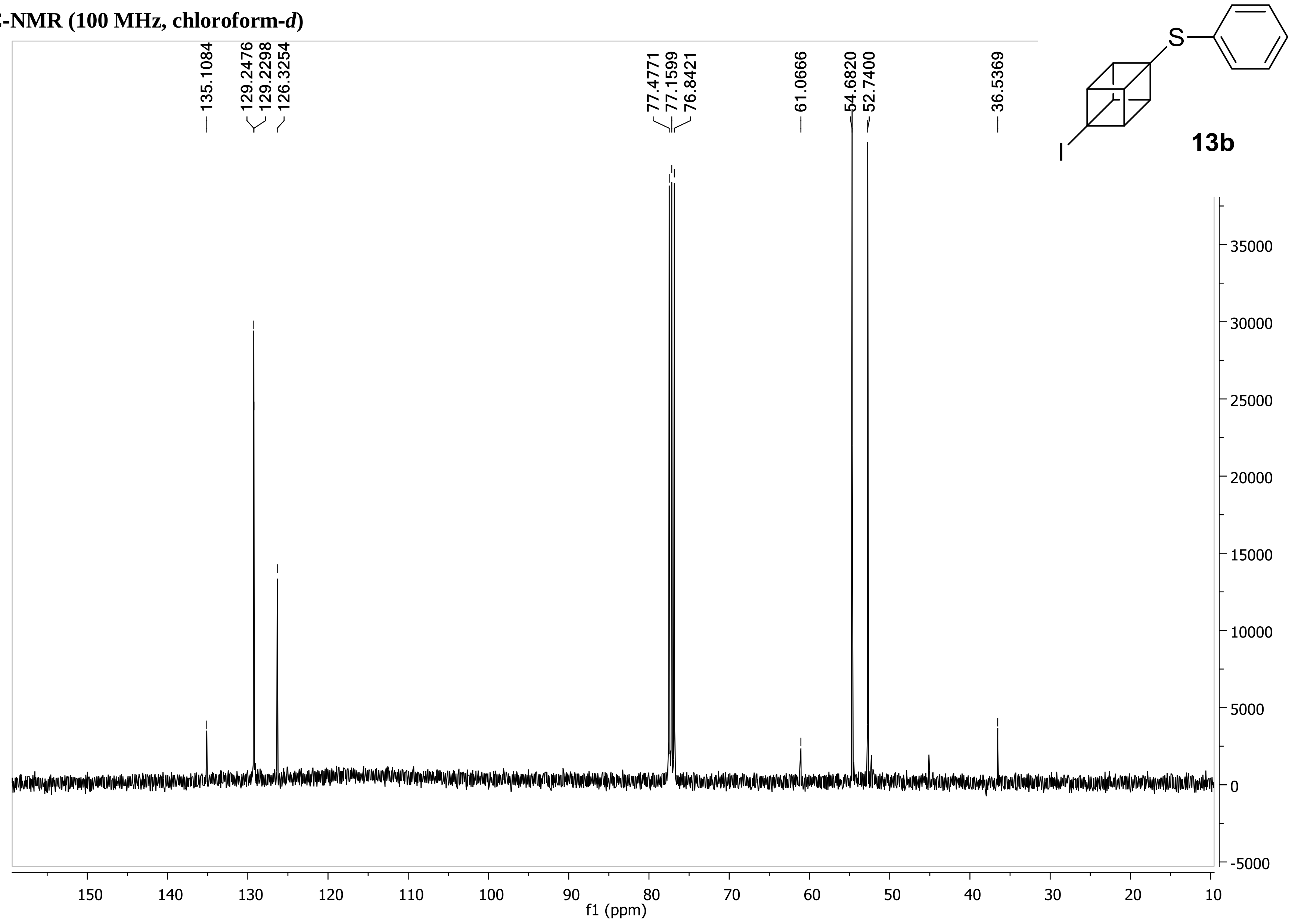

13b

(4-iodocuban-1-yl)(naphthalen-2-yl)sulfane (13c)

^{1}H- NMR (400 MHz, chloroform-*d*)

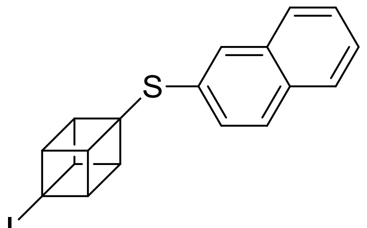

13c

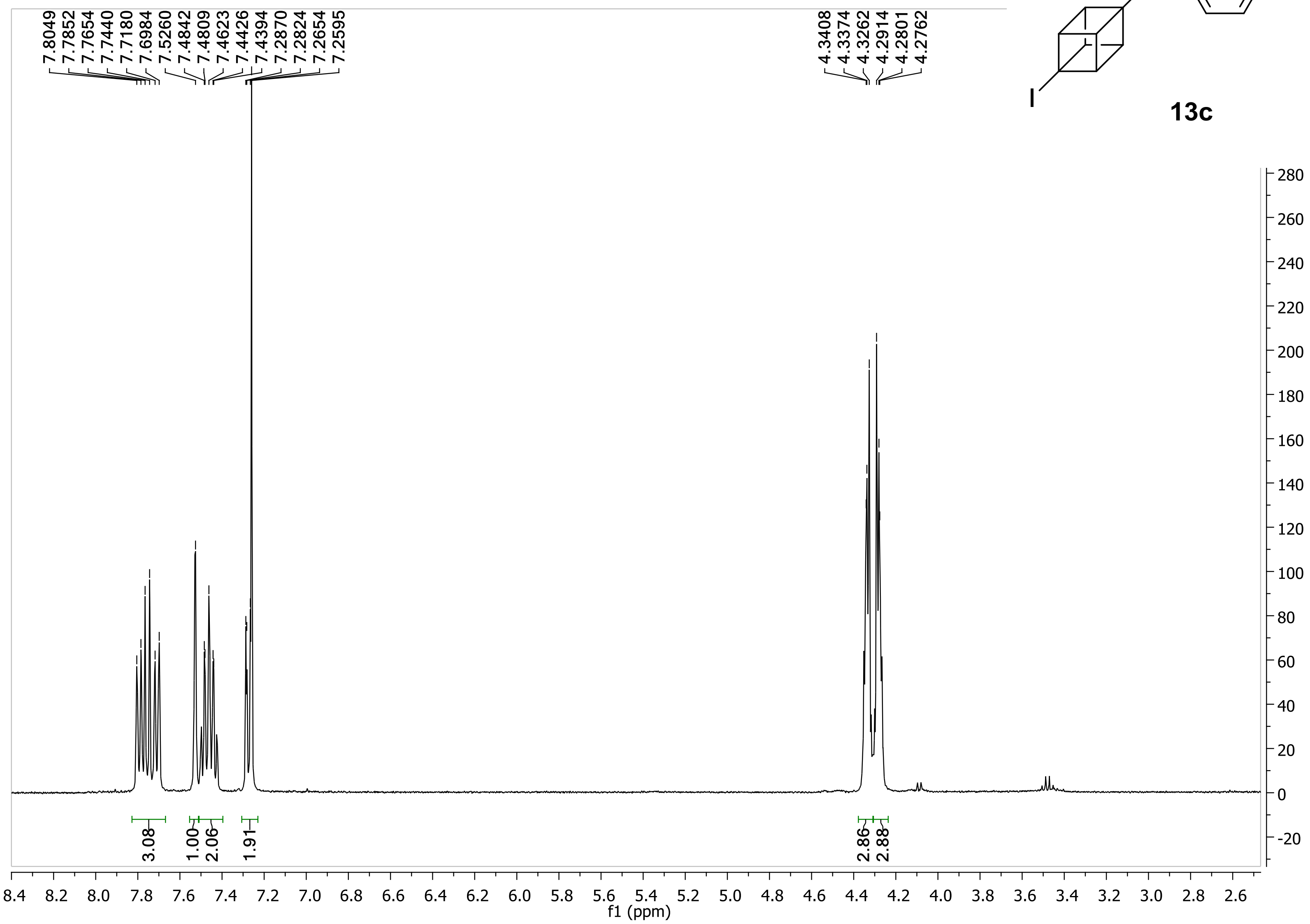

^{13}C-NMR (100 MHz, chloroform-*d*)

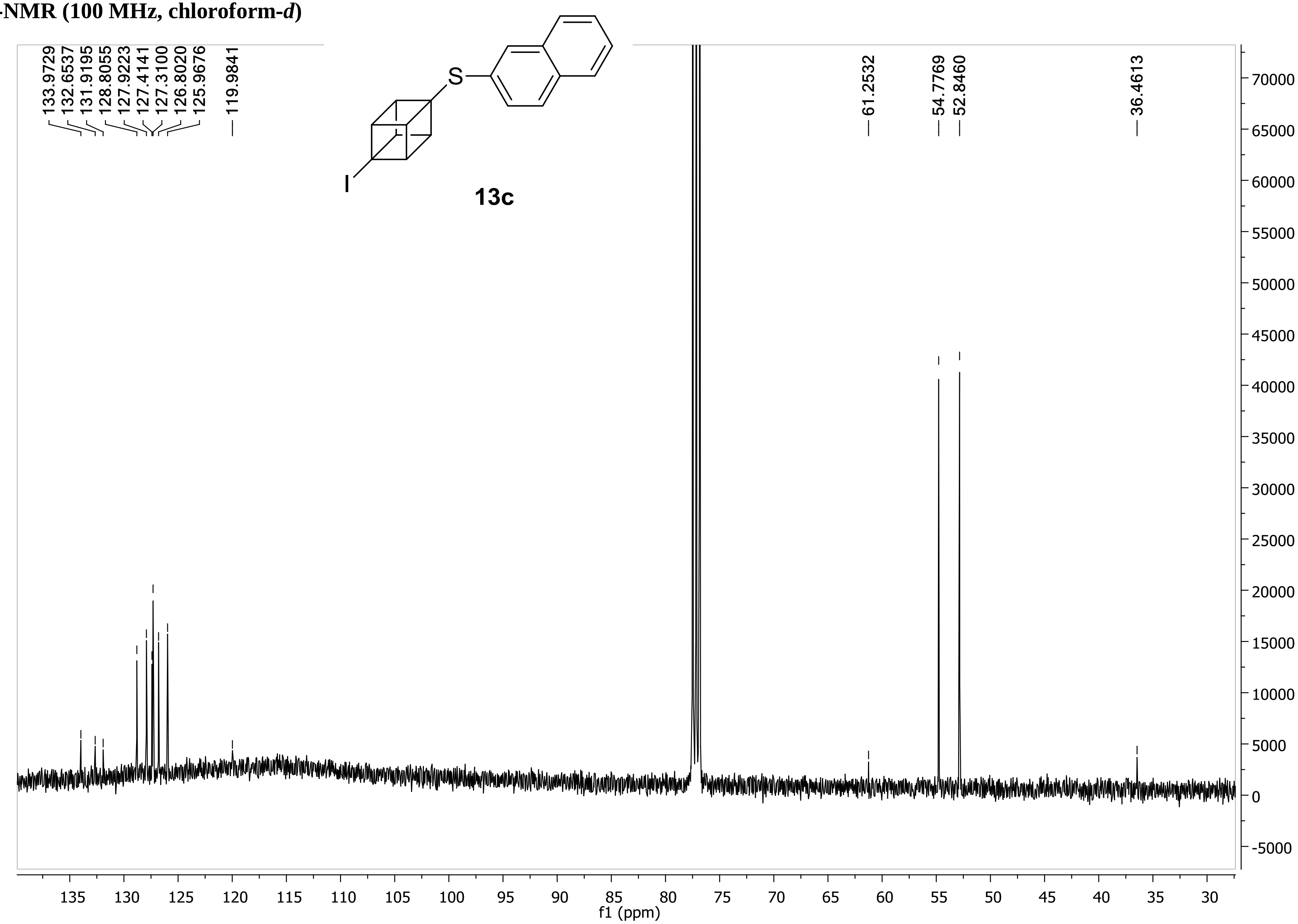

Cuban-1-yl(4-methoxyphenyl)sulfane (14a)

^{1}H- NMR (400 MHz, chloroform-*d*)

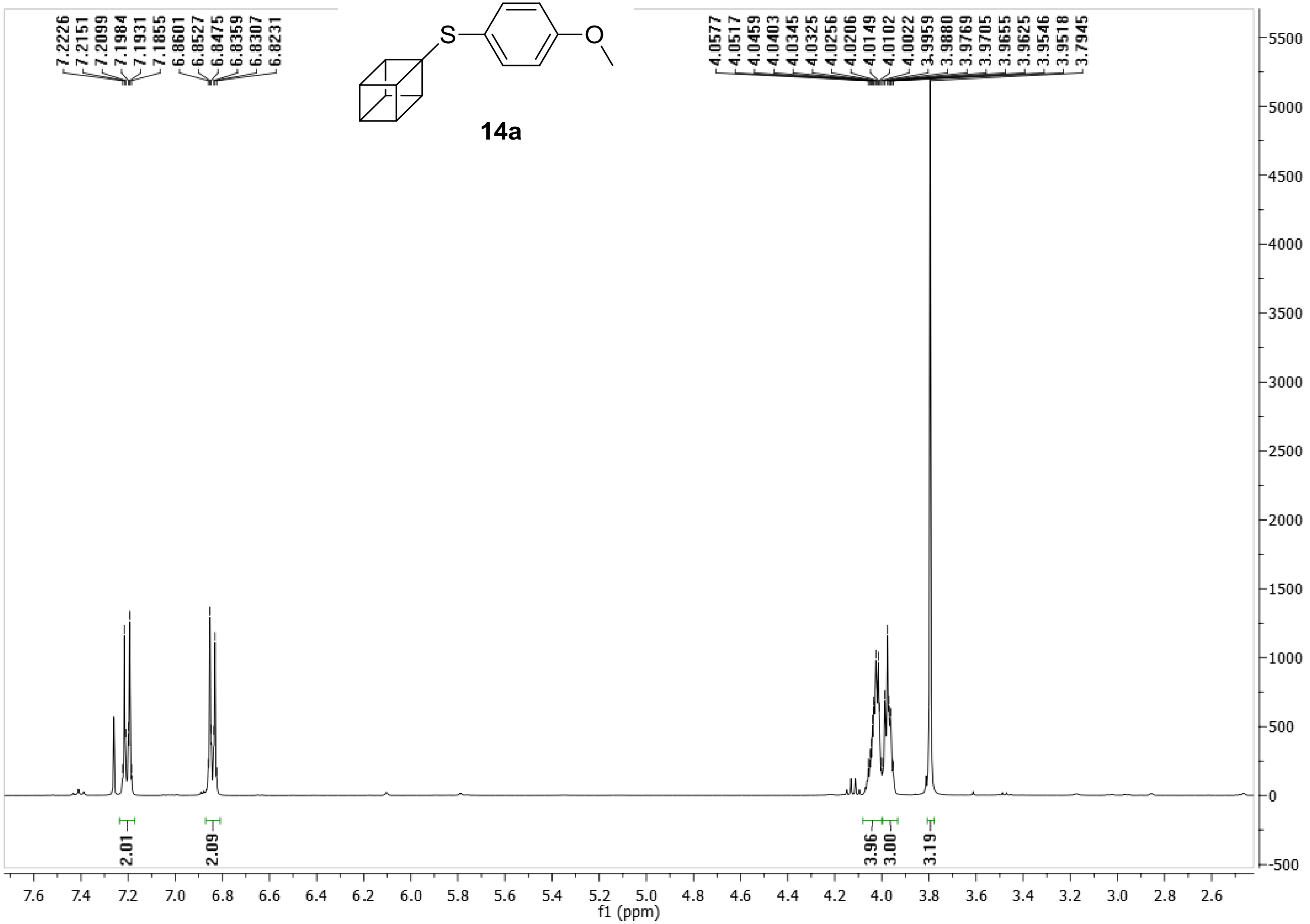

^{13}C-NMR (100 MHz, chloroform-*d*)

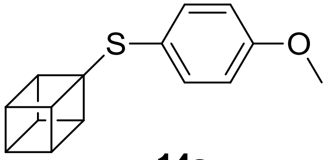

14a

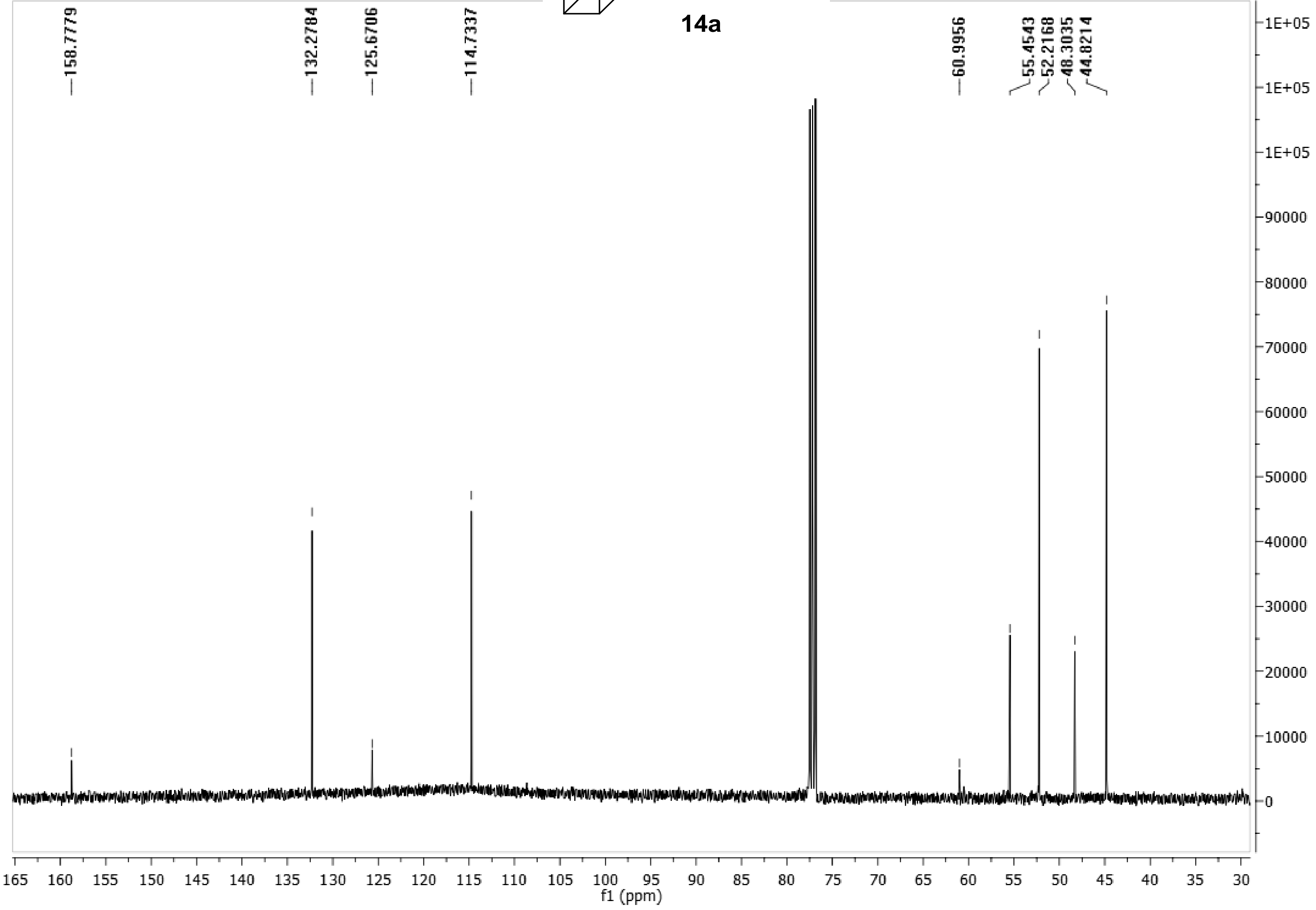

COSY (chloroform-*d*)

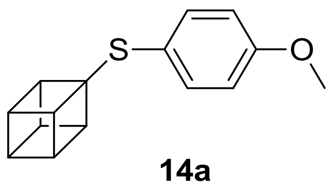

14a

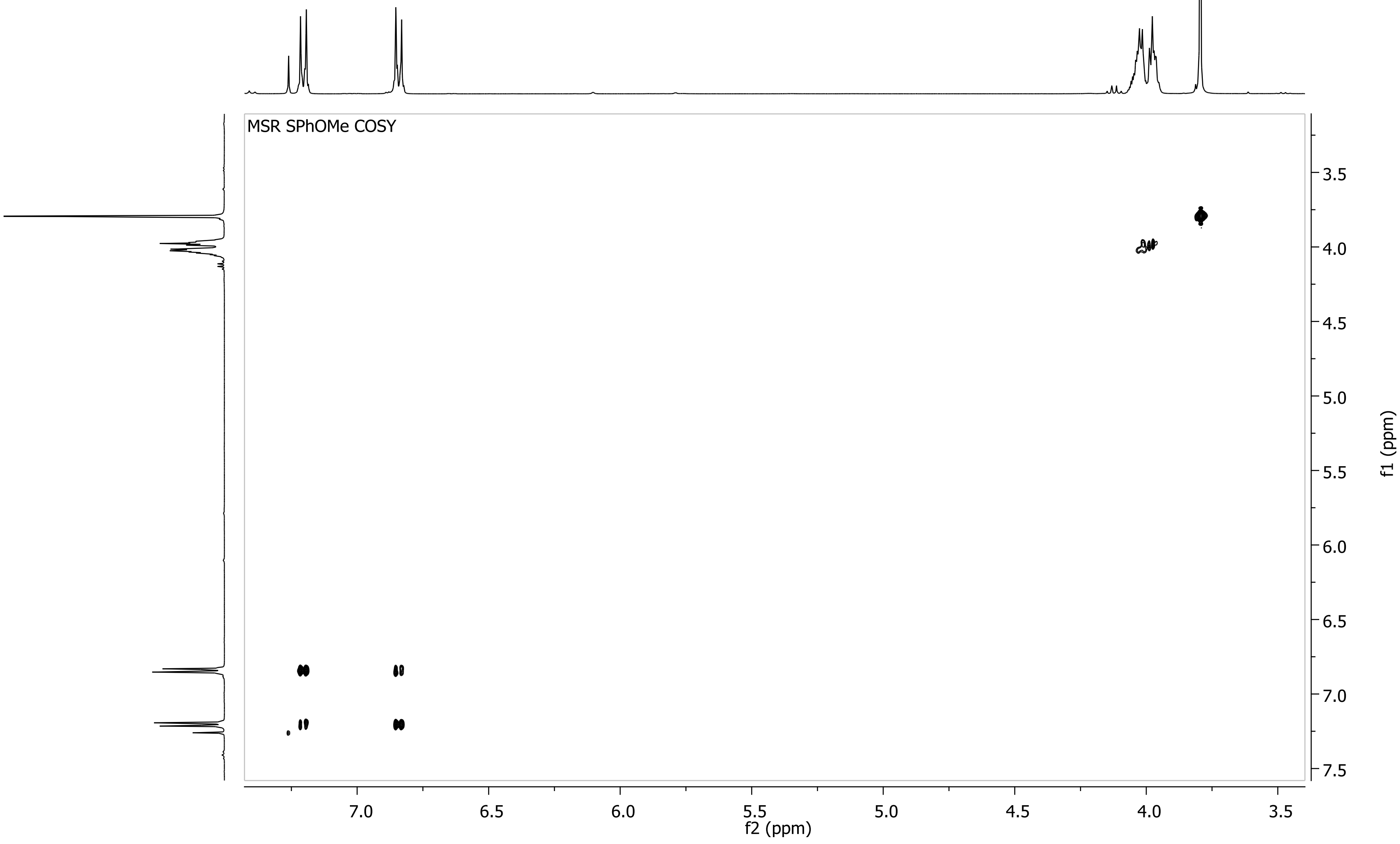

HSQC-DEPT (chloroform-*d*)

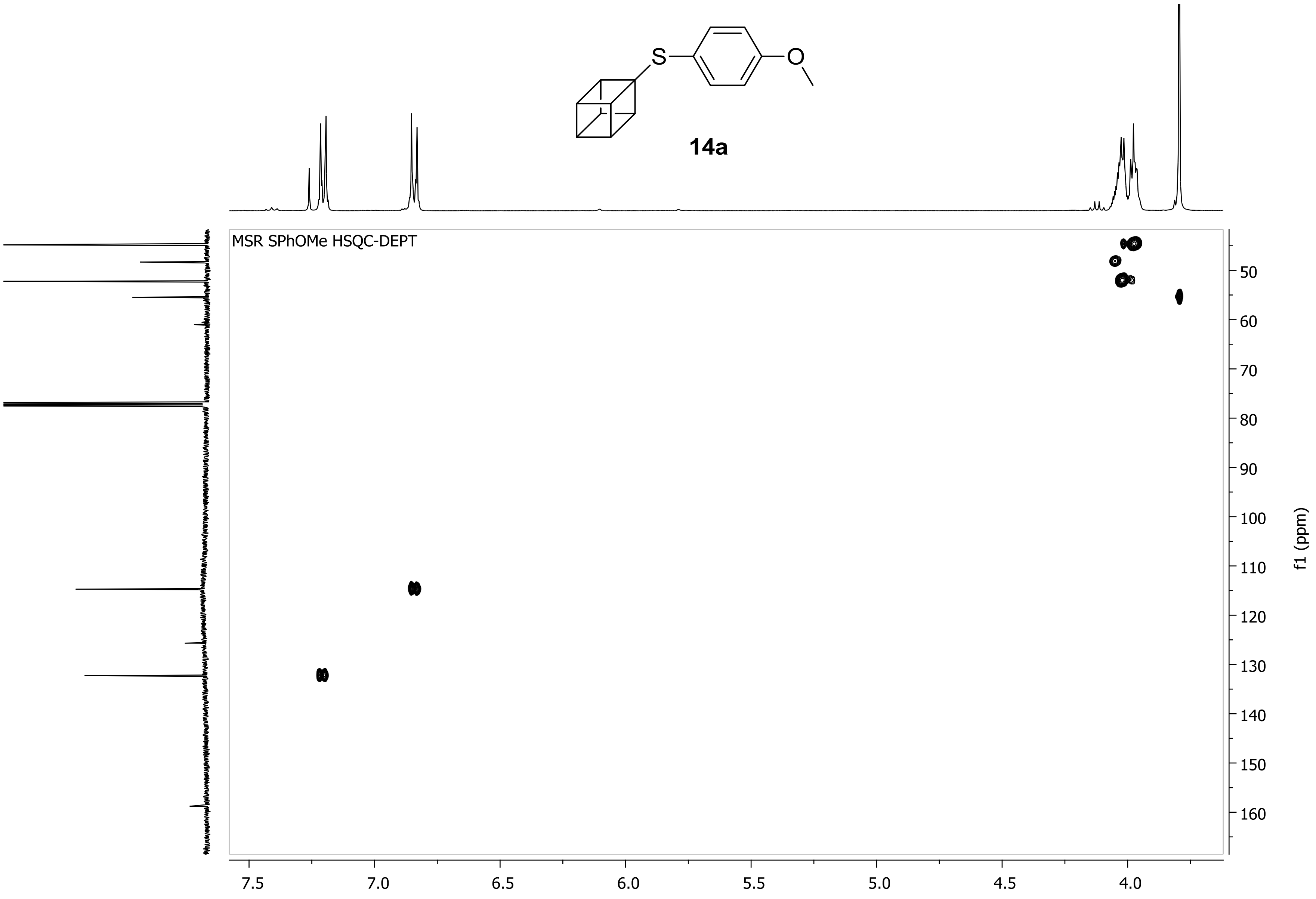

HMBC (chloroform-*d*)

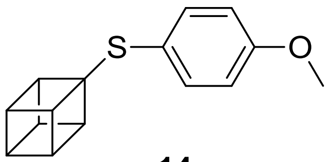

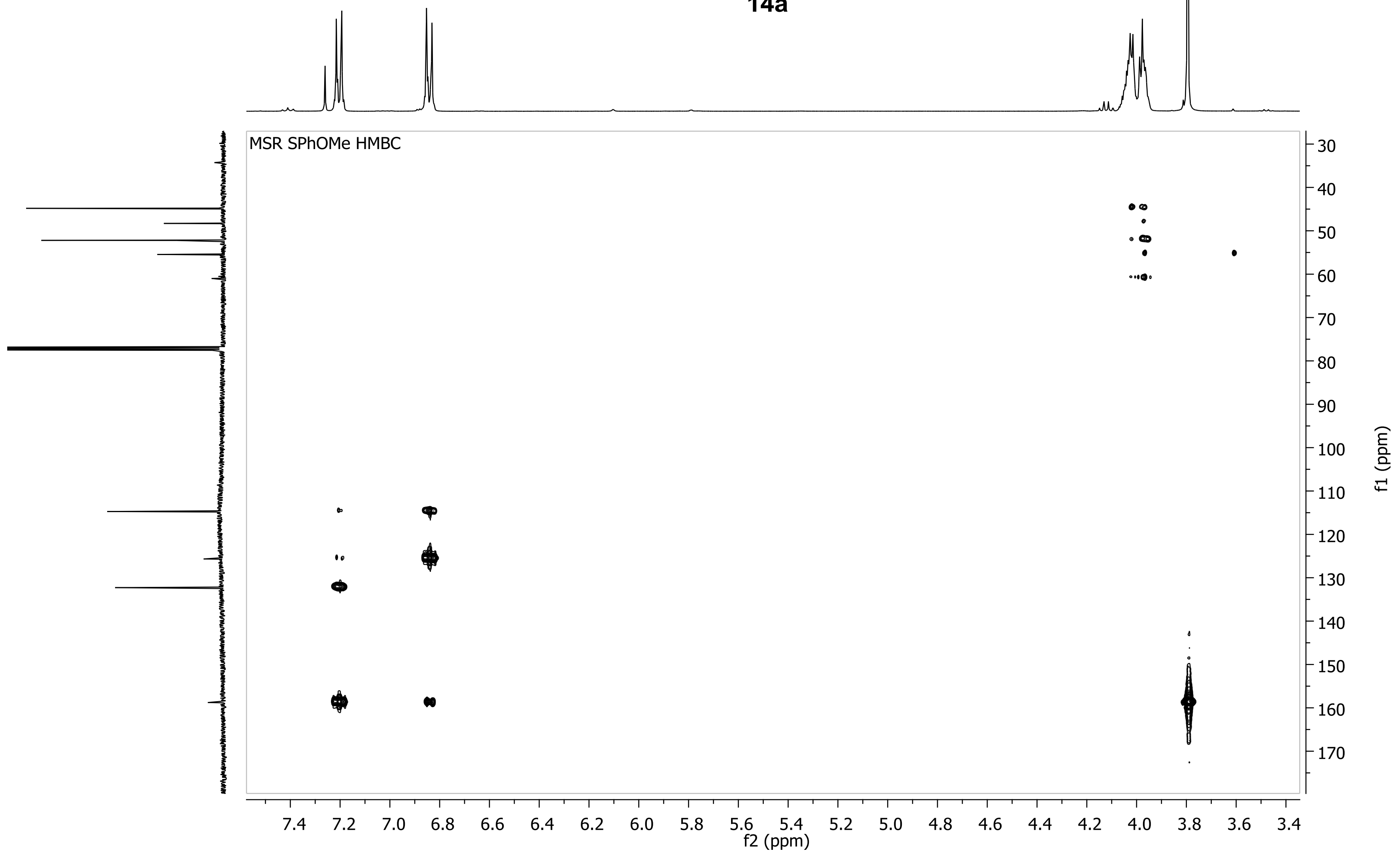

Cuban-1-yl(phenyl)sulfane (14b)

^{1}H- NMR (400 MHz, chloroform-*d*)

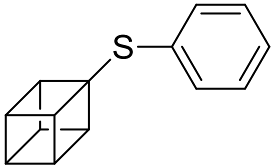

14b

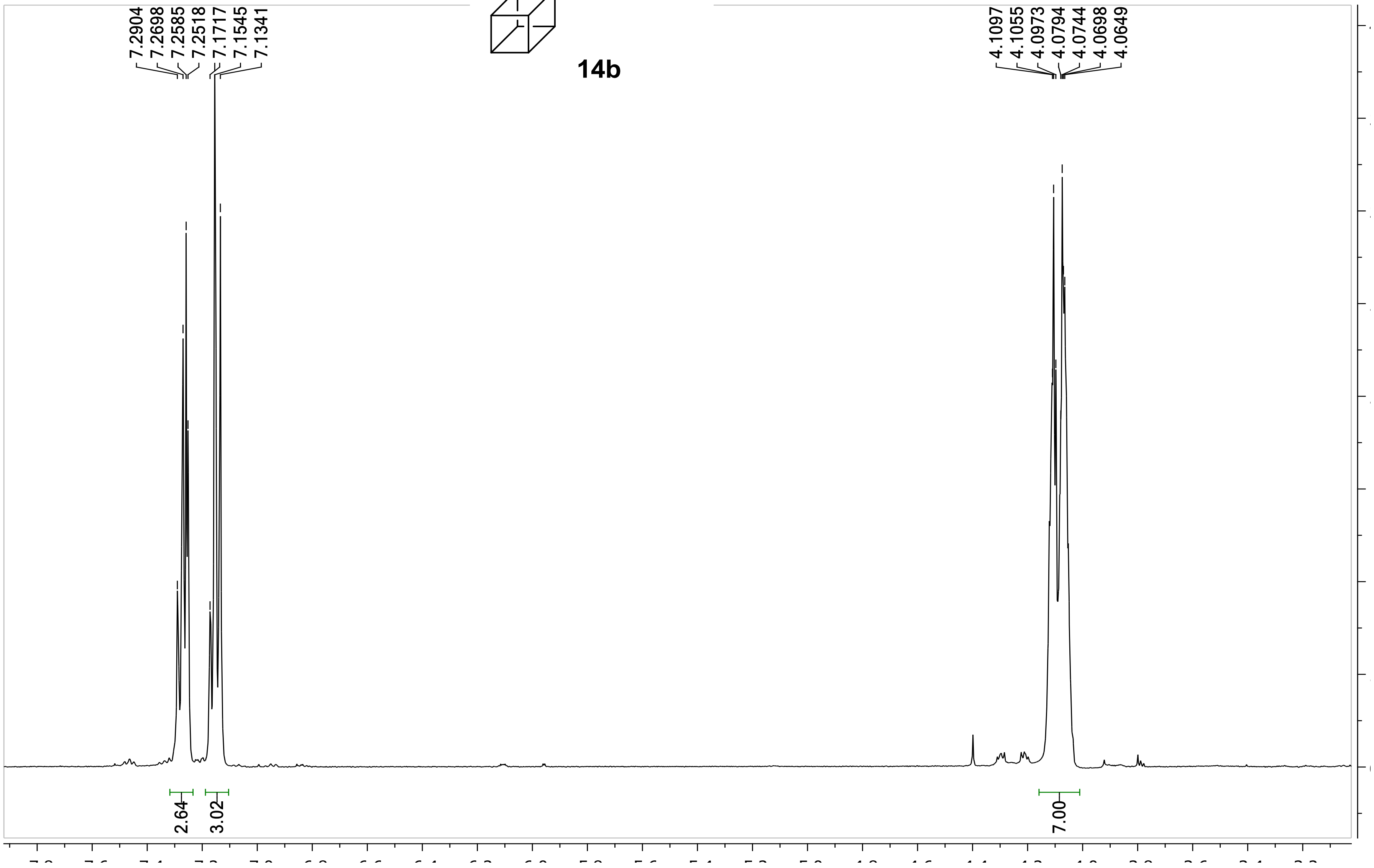

^{13}C-NMR (100 MHz, chloroform-*d*)

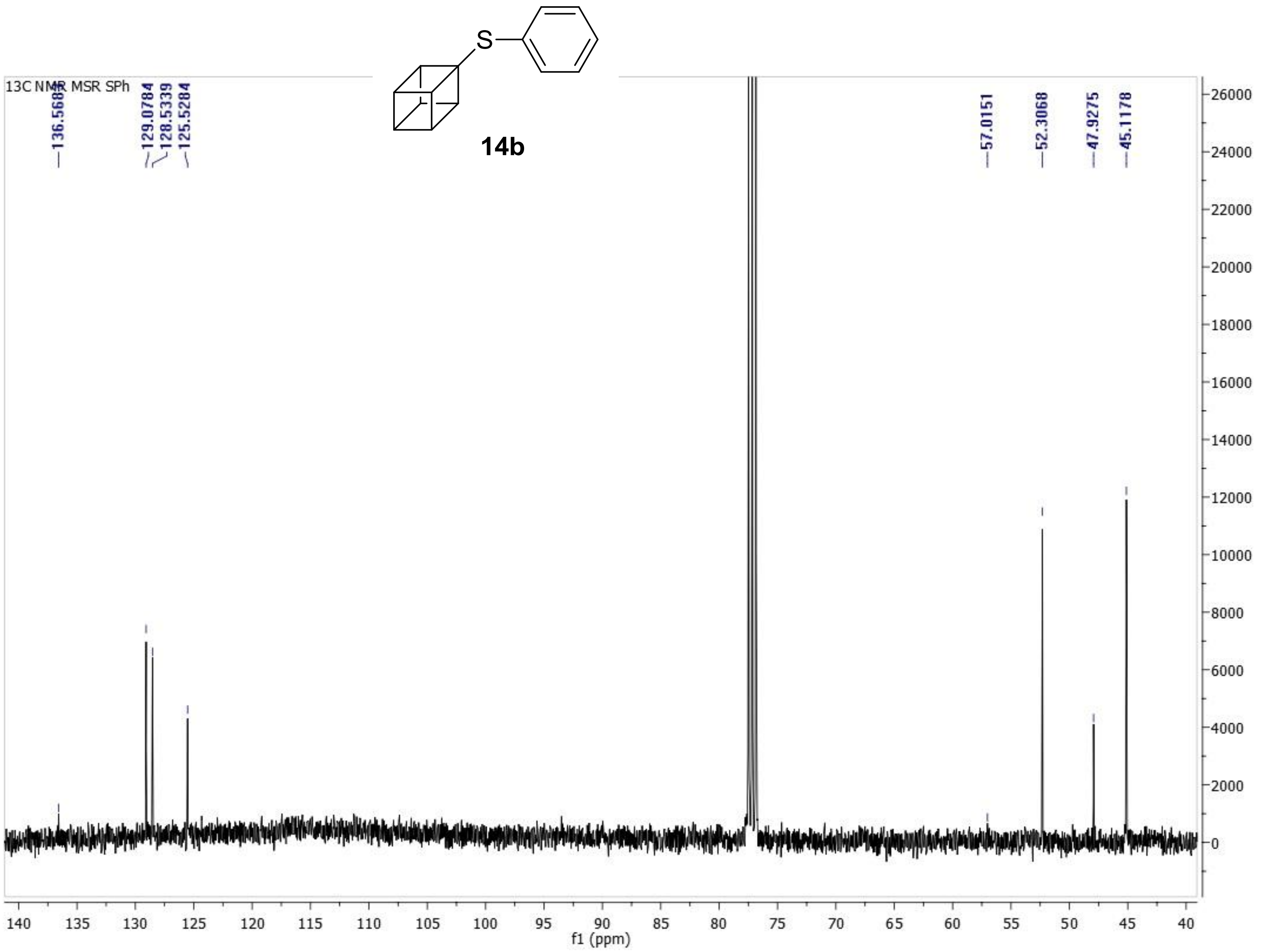

Cuban-1-yl diphenylphosphine oxide (14d)

^{1}H- NMR (400 MHz, chloroform-*d*)

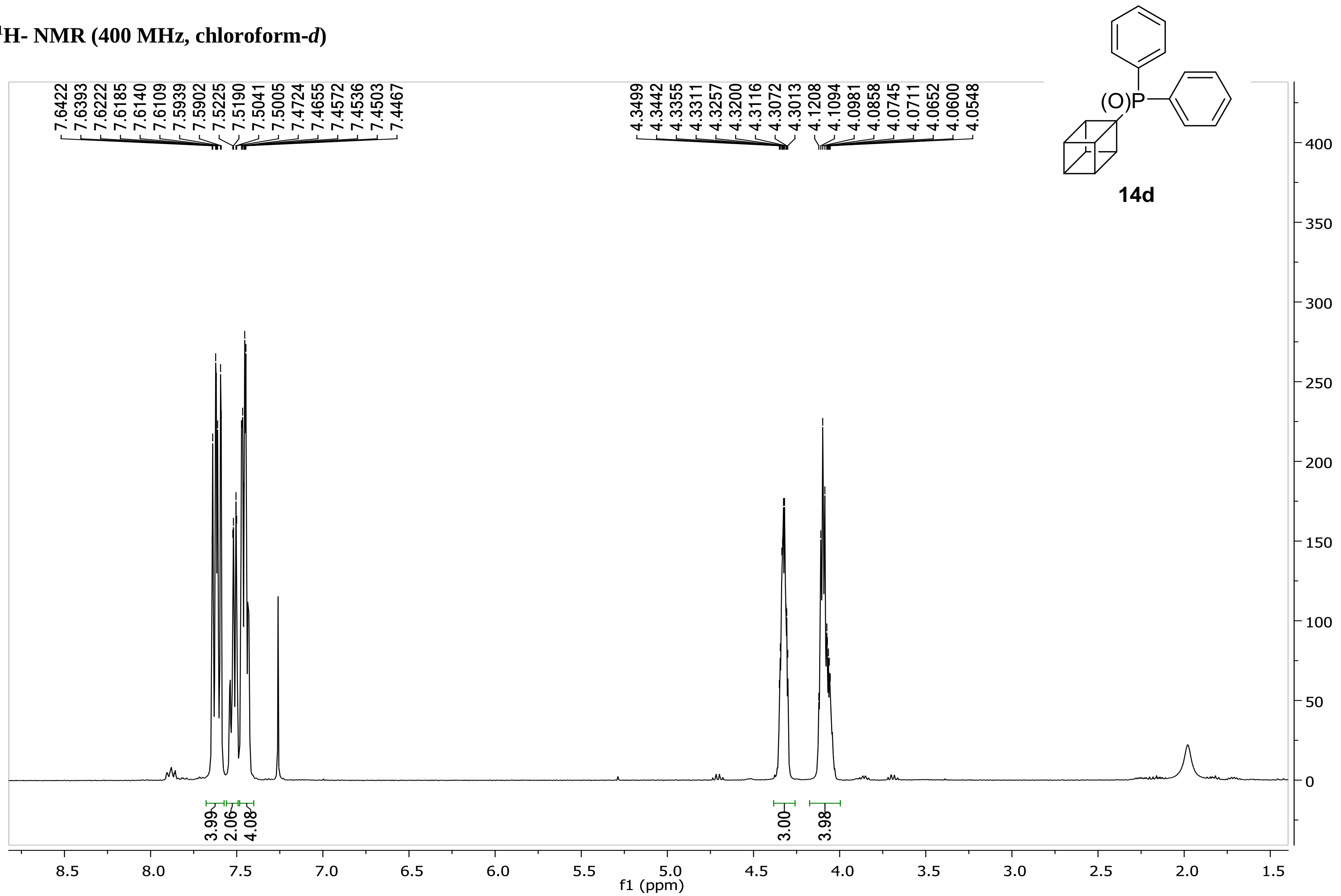

^{13}C-NMR (100 MHz, chloroform-*d*)

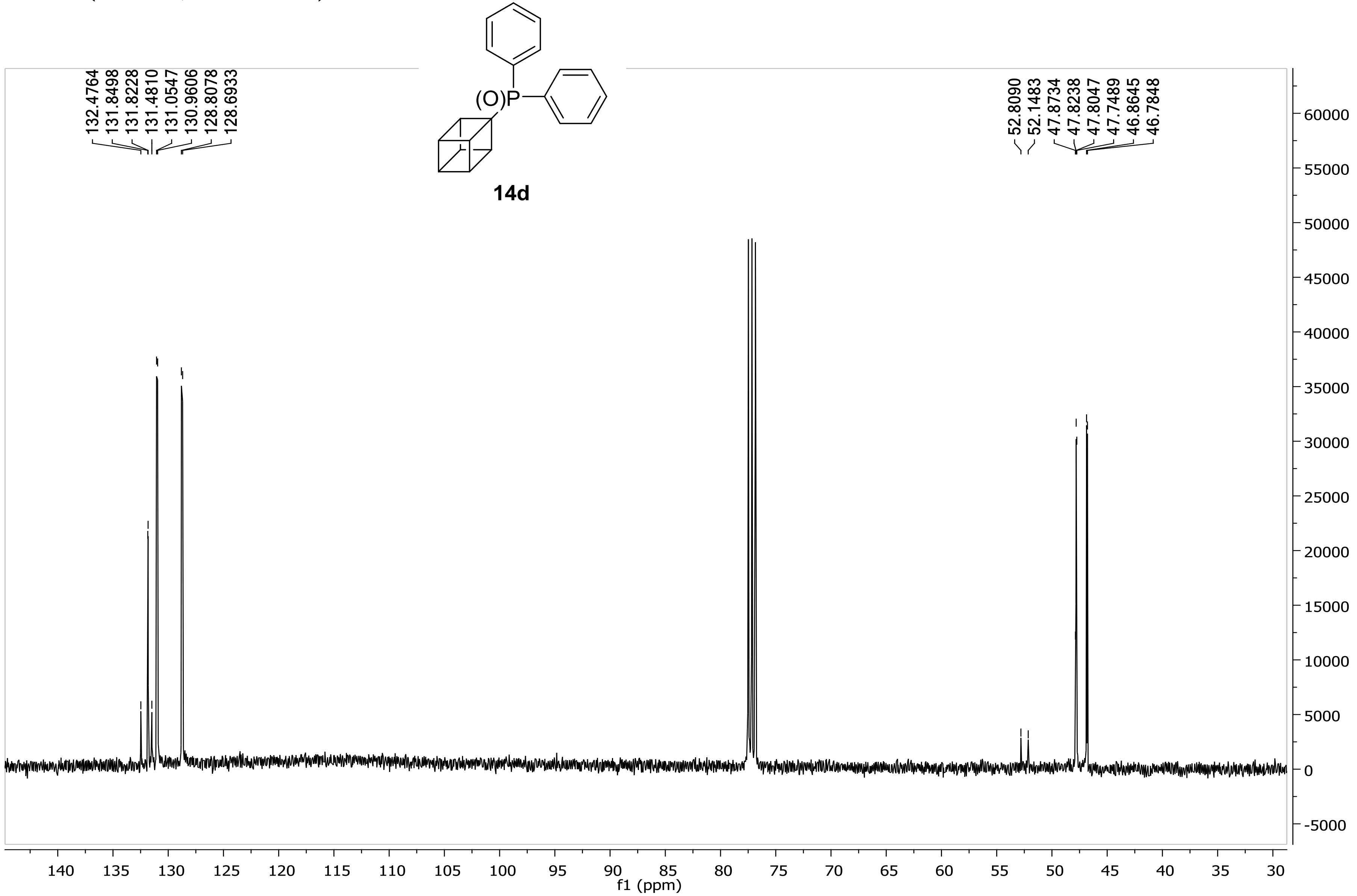

^{13}C-NMR (100 MHz, chloroform-*d*)

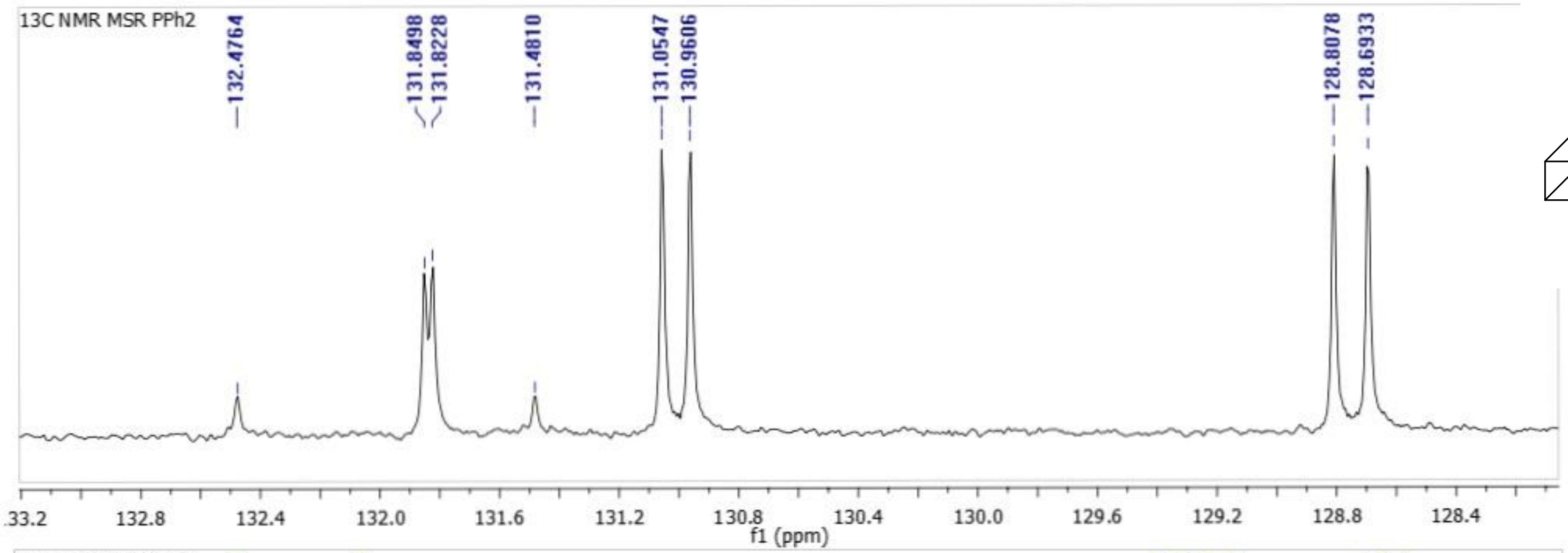

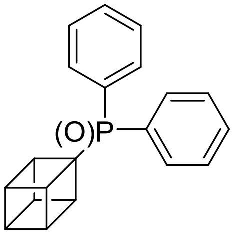

14d

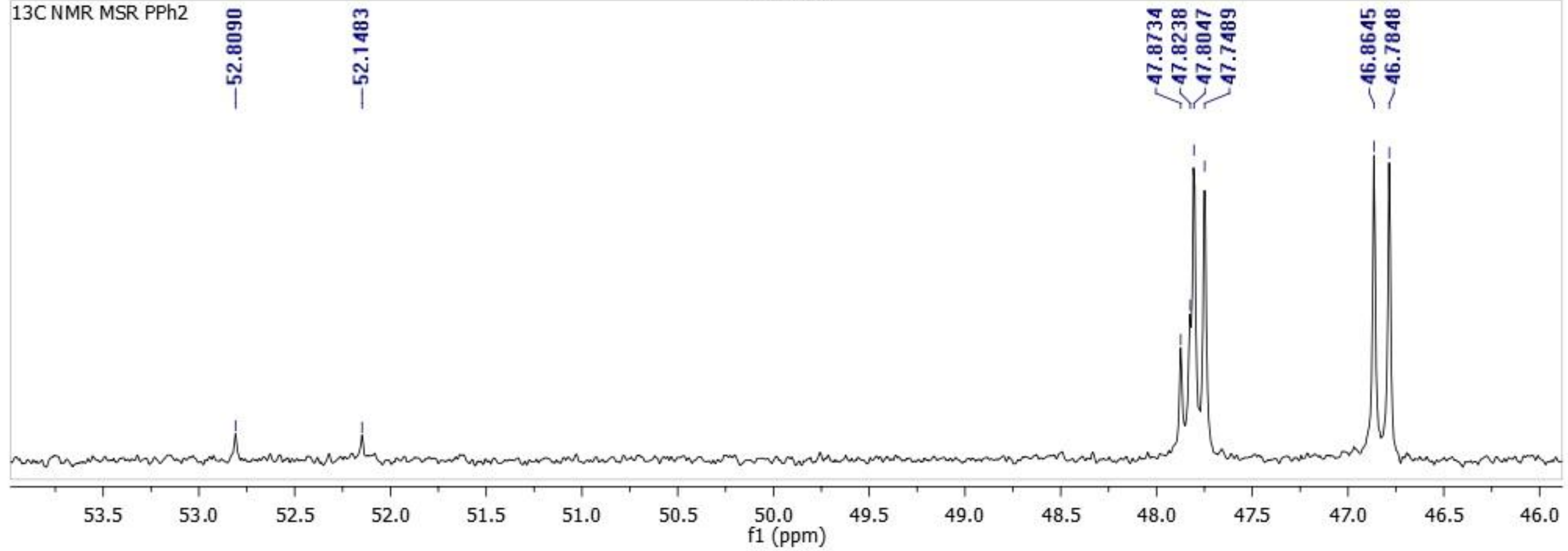

IR

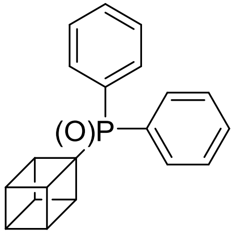
(O)P

14d

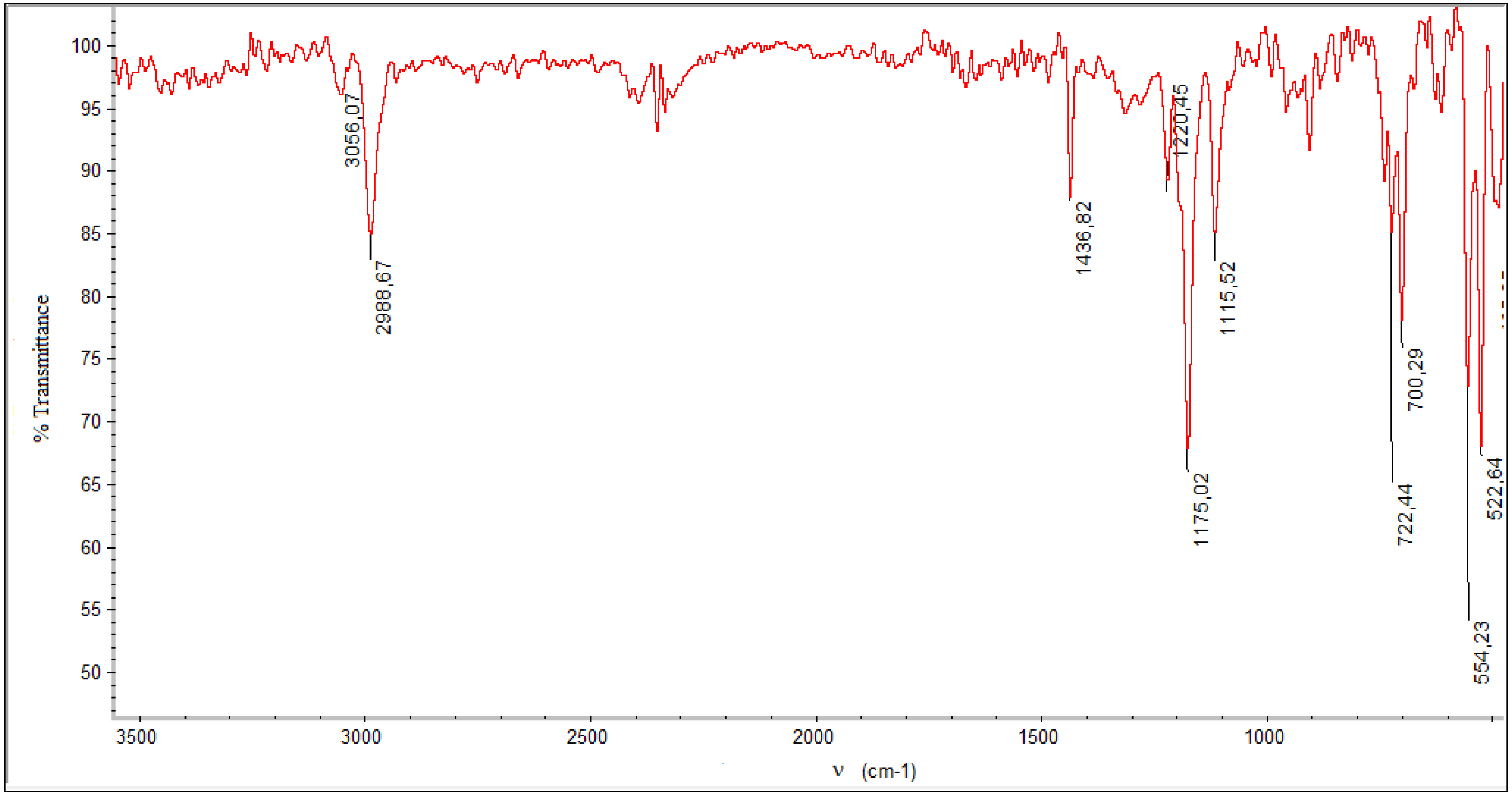
% Transmittance
100
95
90
85
80
75
70
65
60
55
50
3500
3000
2500
2000
1500
1000
ν (cm-1)
3056,07
2988,67
1436,82
1220,45
1175,02
1115,52
722,44
700,29
554,23
522,64

3. Computational Information

3.1 Test of different solvent´s models

Since the reactions were carried out in liquid ammonia but this solvent was not available in g09, the solvent employed was methanol (as in previous works from the group). To check the differences arisen from different solvent approximations the energies of 3 molecules were computed with methanol, DMSO and liquid ammonia build using parameters available in literature. (Sieffert, N.; Thakkar, A.; Bühl, M. ***Chem. Commun.***, 2018,**54**, 10431-10434; Budén, M. E; Dorn,V.B.; Gamba, M.; Pierini, A.B.; Rossi; R. A. *J. Org. Chem*., 2010, 75, 2206-2218) .

Almost no differences were observed until the 4th decimal of the energy (~0.1 to 0.3 kcal/mol). With this small energy we considered that the results obtained in the study of the mechanism would not significantly change in either (DMSO, NH3, or methanol used instead of NH3)

Molecule	Solvent	SCF Energy	ZPE Corr.	Sum of ee and thermal □G
iodocubanoradical	Methanol	-605.7025463	0.111679	-605.623883
	DMSO	-605.7025935	0.111675	-605.623934
	NH3	-605.7024765	0.111684	-605.623808
15-rad	Methanol	-536.6591589	0.164962	-536.530933
	DMSO	-536.6592657	0.164956	-536.531041
	NH3	-536.6590012	0.164971	-536.530772

3.2 Calculation of the redox potentials of phenylsulphide and dipheylphosphide anions

The overall calculated reaction is presented in the thermodynamic cycle shown in scheme 1 and eq. 1.

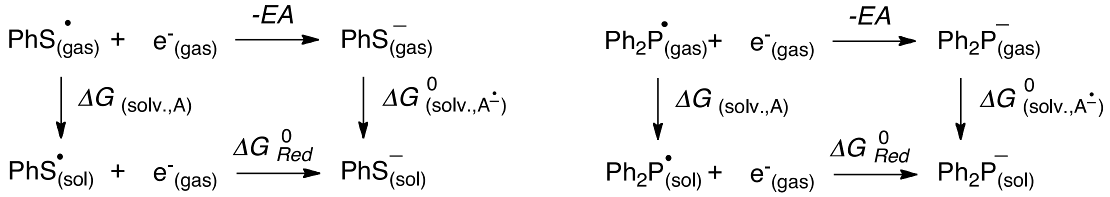

scheme 1

In previous works, this equation has been employed for the calculation of reduction.[1-4] In some cases the $\Delta\Delta G^0_{(Solv)}$ was calculated over the gas phase geometries, in other cases different solvent models were employed to improve its quality and hence the ΔG^0_{Red}. In the present work the ΔG^0_{Red} was directly calculated from the optimized geometries in solvent by employing the IEF-PCM continuum solvation model,[5] including the thermal corrections and the changes in enthalpy and entropy.

$$\Delta E=\Delta G^0_{(Red)}=-EA+\Delta\Delta G^0_{(Solv)} \quad (1)$$

Finally, the $\Delta G^{0}_{\text{red}}$ is related to the redox potential, E^{0}_{Red}, by the Faraday's equation (2)

$$E^{0}_{Red} = \frac{-\Delta G^{0}_{Red}}{n\,F} \quad (2)$$

where n is the number of electrons transferred (in this case is equal to 1) and F is the Faraday constant (96485 C mol^{-1} or 23.061 kcal mol^{-1} V^{-1}).
According to these equations, the Redox potential of both molecules was calculated:

Molecule	Electronic Energy (hartree)	ZPE Correction (hartree)	ΔG^{0} + ZPE (hartree)	ΔG^{o} (red) $(kcal/mol)$	E^{o}_{red} (V)
$PhS^{\bullet}$	-629.6816788	0.090641	-629.621524		
PhS^{-}	-629.8680842	0.09096	-629.806921	-116.338	~5.05
$Ph_2P^{\bullet}$	-804.4192103	0.182964	-804.275287		
Ph_2P^{-}	-804.5510342	0.182091	-804.406905	-82.591	~3.58
Method: M062X/def2TZVP – PCM: methanol					

Geometries. Spin densities of radicalary species:

$PhS^{\bullet}$ (radical)

C	-1.444035094526	1.318789519713	-0.036036930195
C	-0.051375692512	1.311955486295	-0.035550753254
C	0.639497963100	2.522649003509	-0.036083494537
C	-0.051264232651	3.733405937645	-0.037099153054
C	-1.443924330165	3.726699608243	-0.037571706583
C	-2.144688423749	2.522776659088	-0.037044008851
H	-1.977359938134	0.379207521172	-0.035623674105
H	0.485666821540	0.373744783189	-0.034758893870
H	0.485866044717	4.671566783848	-0.037527003522
H	-1.977162370089	4.666330631850	-0.038356357947
H	-3.224486895331	2.522825927394	-0.037412261598
S	2.456870727801	2.522566748055	-0.035441172485

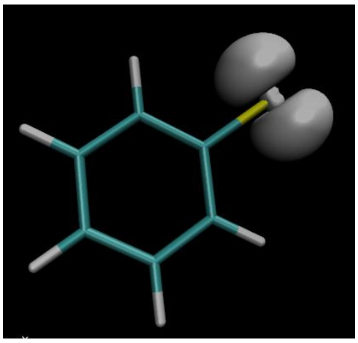

PhS^{-} (anion)

C	-1.449389385640	1.321980547058	-0.036041429827
C	-0.056597265234	1.322902023100	-0.035552946870
C	0.674975060683	2.522647438050	-0.036065525356
C	-0.056486706288	3.722459947957	-0.037097438311
C	-1.449278846116	3.723509139815	-0.037570306870
C	-2.159120419136	2.522777322472	-0.037048694248
H	-1.980858736711	0.379533935875	-0.035635673038
H	0.481350075112	0.384651121853	-0.034766240143
H	0.481549806580	4.660660052191	-0.037534460315
H	-1.980661706514	4.666004276583	-0.038349085301
H	-3.239657209479	2.522826479787	-0.037415637952
S	2.487779912742	2.522566325258	-0.035427971771

$Ph_2P^{\bullet}$

P	1.034921427728	1.059632864399	0.032440693934
C	-0.113715728954	2.478109958523	-0.260713690547

C	-1.373642491491	2.609285251139	0.341271136960
C	0.367838812084	3.527712809825	-1.056882562387
C	-2.136962271874	3.753864857544	0.133306701629
H	-1.753772331784	1.822500150571	0.978100265882
C	-0.399549919750	4.668221971335	-1.268493161214
H	1.344585930266	3.445238372685	-1.516194287456
C	-1.655016023824	4.783730176873	-0.673982947843
H	-3.105126296187	3.845141999647	0.604984156811
H	-0.018663560925	5.464583077609	-1.891618910159
H	-2.250954926517	5.670714063951	-0.834271123066
C	-0.101820057556	-0.375002609689	0.291222450011
C	0.376333661449	-1.423375412061	1.091231843267
C	-1.346794312377	-0.518893876636	-0.338348900187
C	-0.380378636545	-2.574952465485	1.279667576236
H	1.341974002627	-1.331259450436	1.571751202693
C	-2.099422786895	-1.674438150323	-0.153303581231
H	-1.723565870578	0.266565026007	-0.978740290306
C	-1.621315868876	-2.703093425210	0.657785612210
H	-0.002313051279	-3.370252037855	1.905865217513
H	-3.056093860932	-1.775098058917	-0.646021756976
H	-2.208704387809	-3.598872493497	0.799604864225

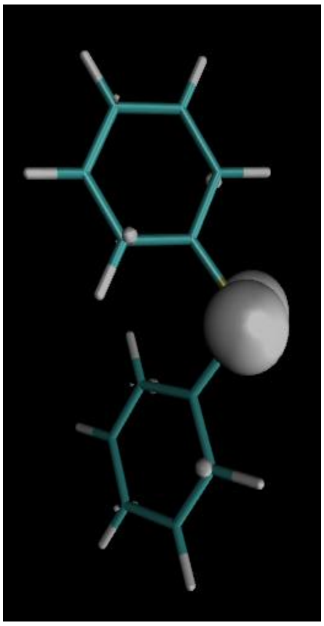

Ph_2P^-

P	1.041592252280	1.055809789045	0.014801407785
C	-0.112927529885	2.485423452075	-0.288706804885
C	-1.400113143847	2.628046810623	0.265100810109
C	0.377131989662	3.570167234260	-1.043223042657
C	-2.155251299128	3.780632050307	0.063055406863
H	-1.808806222626	1.831299475695	0.872393117918
C	-0.368896286858	4.729271320389	-1.232507531536
H	1.360023791707	3.490867872363	-1.492370882703
C	-1.647881185096	4.843336799967	-0.685720822413
H	-3.141550353665	3.854867206214	0.502891262538
H	0.044282891924	5.541532152351	-1.816224998021
H	-2.233779312506	5.738678178083	-0.838281092836
C	-0.101674566898	-0.386030235897	0.304347429572
C	0.384185028471	-1.459425920934	1.077218019553
C	-1.374180480116	-0.547897865158	-0.277590029356
C	-0.352062788073	-2.626311145731	1.257739006855
H	1.355564888817	-1.365178801219	1.547983127234
C	-2.120007526720	-1.707957948536	-0.084392519722
H	-1.777890322602	0.239821702942	-0.899872640881
C	-1.616822604164	-2.759395842440	0.683045114321
H	0.057312265691	-3.429547479823	1.856367469048
H	-3.095164365464	-1.797180322813	-0.545825311718
H	-2.195243670906	-3.660765881762	0.828434014934

3.3 Hypothetical mechanism involving formation of 1,4-dehydrocubane A

We propose that 1,4-diiodocubane **2** receives an electron from the nucleophile (ArS^-/Ph_2P^-) in a photoinduced process through a dissociative-ET pathway leading directly to 4-iodocubyl radical **17**. This intermediate can afford 1,4-dehydrocubane **A** by homolytic dissociation (with formation of iodine radicals) or by ET (with formation of iodide ions). Another possibility is its reaction with the anion, which is in excess, to afford the radical anion of the mono-iodosubstituted cubane **13** (Scheme 1-SI). Calculations indicated that the preferred reaction is the coupling with the

nucleophile to allow a typical $S_{RN}1$ pathway. Relevant energetic factors (ΔG°) of the three mechanistic possibilities are presented in the next Table 1-SI.[6,7]

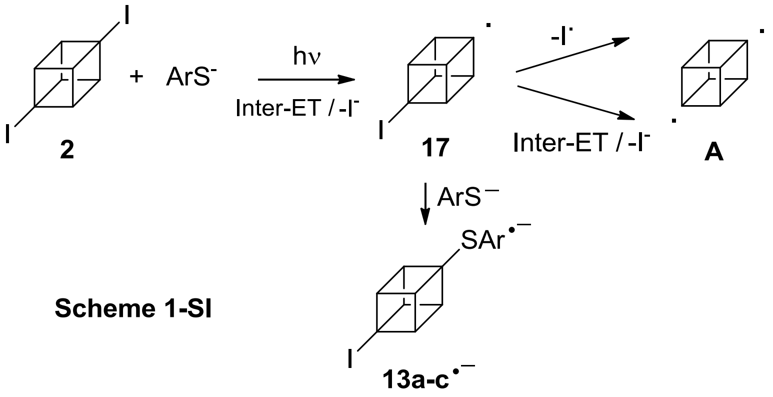

Scheme 1-SI

Table 1-SI. Energetic values involved in reactions shown at Scheme 1-SI

Reaction				ΔG° (kcal/mol) (298 K)
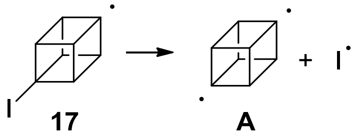				**53.656**
Compounds	Electronic Energy (hartrees)	ZPE (hartrees)	ΔG+ZPE (hartrees)	
4-Iodocubyl radical, **17**	-605.7025463	0.111679	-605.623883	
1-4 dehydrocubane diradical, **A**	-307.9851116	0.077481	-307.907631	
Iodide radical	-297.6132423	0.000000	-297.630745	
Reaction				ΔG° (kcal/mol) (298 K)
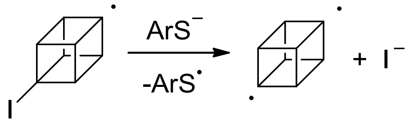				**41.822**
Compounds	Electronic Energy (hartrees)	ZPE (hartrees)	ΔG+ZPE (hartrees)	
4-Iodocubyl radical, **17**	-605.7025463	0.111679	-605.623883	
Thiophenoxide anion, **4**⁻	-629.8680842	0.09096	-629.806921	
1-4 dehydrocubane diradical, **A**	-307.9851116	0.077481	-307.907631	
Iodide anion	-297.8181529	0.000000	-297.835001	
Thiophenyl radical, **4**·	-629.6816788	0.090641	-629.621524	
Reaction				ΔG° (kcal/mol) (298 K)
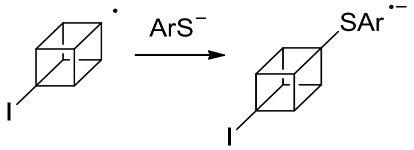				**11.0**
Compounds	Electronic Energy (hartrees)	ZPE (hartrees)	ΔG+ZPE (hartrees)	

4-Iodocubyl radical, **17**	-605.7025463	0.111679	-605.623883	
Thiophenoxide anion, **4**⁻	-629.8680842	0.09096	-629.806921	
4-iodocubyl-phenylsulfide radical anion, **13b**·⁻	-1235.5546359	0.202085	-1235.397200	
TS for the coupling	-1235.5476664	0.201420	-1235.391243	24.825 (Activation Energy)

All the calculations were performed with the Gaussian09 program,[8] the M06-2X DFT functional and the def2TZV basis set for C, H, O, S and P were employed. The def2TZVP[9] basis set and pseudo-potential was used for I. Calculations were performed with full geometry optimization including in all cases the effect of the solvent (methanol as polar solvent) through the Tomasi's polarized continuum model (IEFPCM)[5,10] as implemented in the Gaussian package. After refinement the characterization of stationary points was done by Hessian matrix calculations, with all positive eigenvalues for a minimum and only one negative eigenvalue for the TSs. The energy informed for TSs and radicals includes zero-point corrections.

Geometries:
4-Iodocubyl radical, **17**

C	-0.014345381477	-0.014648103207	-0.014015286728
C	-0.005284423796	-0.005322099047	1.556420974340
C	1.570938211811	-0.007604415900	1.522880294516
C	1.555668188226	-0.011751256001	-0.053607207463
C	1.527210130492	1.563919039645	-0.098699814437
C	-0.048706777697	1.555034262158	-0.053658063307
C	-0.044915416095	1.570394609689	1.522842072205
C	1.518914870821	1.555417645645	1.466205843640
I	-1.232703670740	-1.263086986913	-1.190015814411
H	-0.620080820437	-0.634870728982	2.186070440799
H	2.129164186334	2.180378297153	-0.752199292284
H	2.207435560399	-0.624505889030	2.142328411990
H	-0.676809870119	2.192035548328	2.142267295812
H	-0.697620557750	2.147489526206	-0.685087489113
H	2.163401385395	-0.646371878440	-0.685028744015

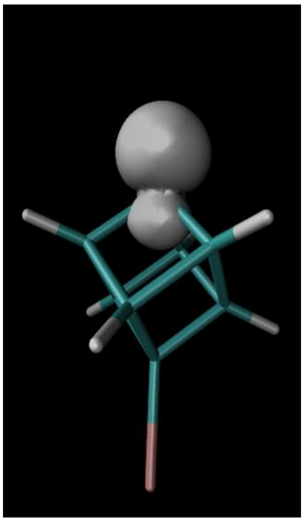

1,4-dehydrocubane diradical, **A**

C	-0.014345381477	-0.014648103207	-0.014015286728
C	-0.005284423796	-0.005322099047	1.556420974340
C	1.570938211811	-0.007604415900	1.522880294516
C	1.555668188226	-0.011751256001	-0.053607207463
C	1.527210130492	1.563919039645	-0.098699814437
C	-0.048706777697	1.555034262158	-0.053658063307
C	-0.044915416095	1.570394609689	1.522842072205
C	1.518914870821	1.555417645645	1.466205843640
I	-1.232703670740	-1.263086986913	-1.190015814411
H	-0.620080820437	-0.634870728982	2.186070440799
H	2.129164186334	2.180378297153	-0.752199292284
H	2.207435560399	-0.624505889030	2.142328411990
H	-0.676809870119	2.192035548328	2.142267295812
H	-0.697620557750	2.147489526206	-0.685087489113
H	2.163401385395	-0.646371878440	-0.685028744015

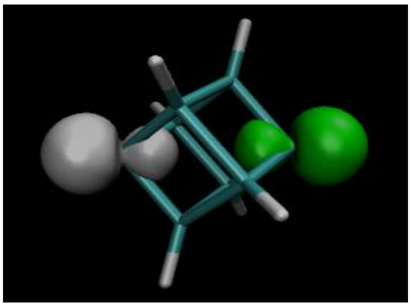

References

1 Namazian, M.; Coote, M. L. *J. Phys. Chem. A* **2007**, *111*, 7227-7232.

2 Speelman, A. L.; Gillmore, J. G. *J. Phys. Chem. A* **2008**, *112*, 5684-5690.

3 Lynch, E. J.; Speelman, A. L.; Curry, B. A.; Murillo, C. S.; Gillmore, J. G. *J. Org. Chem.*, **2012**, *77*, 6423-6430.

4 Namazian, M.; Lin, C. Y.; Coote, M. L. *J. Chem. Theory Comput.* **2010**, *6*, 2721-2725.

5 Tomasi, J.; Mennucci, B.; Cammi, R. *Chem. Rev.* **2005**, *105*, 2999-3093.

6 According to the calculations, the most stable structure of **A** corresponds to a singlet diradical in agreement with previous reports.

7 (a) Hassenrück, H. D.; Radziszewki, J. G.; Balaji, V. ; Murthy, G. S. Mc Kinley, A. J.; David, D.E, Lynch, V. M.; Martin J-D; Michl, J. *J. Am. Chem.Soc*. **1990**, *112*, 873. (b) Hrovat, D. A.; Borden W. T. *J. Am. Chem.Soc*., **1990**, *112*, 875.

8 Gaussian 09, Revision E.01, Frisch, M. J.; Trucks, G. W.; Schlegel, H. B.; Scuseria, G. E.; Robb, M. A.; Cheeseman, J. R.; Scalmani, G.; Barone, V.; Mennucci, B.; Petersson, G. A.; Nakatsuji, H.; Caricato, M.; Li, X.; Hratchian, H. P.; Izmaylov, A. F.; Bloino, J.; Zheng, G.; Sonnenberg, J. L.; Hada, M.; Ehara, M.; Toyota, K.; Fukuda, R.; Hasegawa, J.; Ishida, M.; Nakajima, T.; Honda, Y.; Kitao, O.; Nakai, H.; Vreven, T.; Montgomery, J. A., Jr.; Peralta, J. E.; Ogliaro, F.; Bearpark, M.; Heyd, J. J.; Brothers, E.; Kudin, K. N.; Staroverov, V. N.; Kobayashi, R.; Normand, J.; Raghavachari, K.; Rendell, A.; Burant, J. C.; Iyengar, S. S.; Tomasi, J.; Cossi, M.; Rega, N.; Millam, N. J.; Klene, M.; Knox, J. E.; Cross, J. B.; Bakken, V.; Adamo, C.; Jaramillo, J.; Gomperts, R.; Stratmann, R. E.; Yazyev, O.; Austin, A. J.; Cammi, R.; Pomelli, C.; Ochterski, J. W.; Martin, R. L.; Morokuma, K.; Zakrzewski, V. G.; Voth, G. A.; Salvador, P.; Dannenberg, J. J.; Dapprich, S.; Daniels, A. D.; Farkas, Ö.; Foresman, J. B.; Ortiz, J. V.; Cioslowski, J.; Fox, D. J. Gaussian, Inc., Wallingford CT, 2009.

9 a) Weigend, A.; Ahlrichs, R. *Phys. Chem. Chem. Phys.* **2005**, *7*, 3297. b) Weigend, F. *Phys. Chem. Chem. Phys.*, **2006**, *8*, 1057.

10 Scalmani, G.; Frisch, M. J. *J. Chem. Phys.* **2010**, *132*, 114110.

3.4 Calculations involved in Schemes 4 and 5

3.4.1 Cartesian coordinates and energies (au) at PCM(methanol)-[M06-2X/def2-TZVP]. Spin densities of radicalary species. Imaginary frequencies of Transition States.

I^-

E (PCM(methanol)-[M06-2X/def2-TZVP])= -297.818216336

1

E (PCM(methanol)-[M06-2X/def2-TZVP])= -834.375006343

C	-0.842921	0.039270	0.000572
C	-0.016382	0.777171	-1.087925
C	0.829035	1.449674	0.039020
C	1.816091	0.255589	0.001191
C	0.973797	-0.499235	1.083013
C	-0.009943	0.709386	1.127832
C	0.129349	-1.172471	-0.039534
C	0.967885	-0.431247	-1.122906
H	1.395088	-1.004410	1.945455
H	-0.427500	1.278961	-1.956812
H	-0.415349	1.157082	2.028385
H	1.121419	2.493741	0.069660
H	1.384742	-0.881797	-2.017163
H	-0.166913	-2.215062	-0.071068
C	3.293718	0.350749	0.000055
O	3.927038	1.377435	-0.000804
O	3.855037	-0.860501	-0.000463
C	5.285004	-0.891206	0.000228
H	5.672579	-0.396937	-0.889046
H	5.671153	-0.394878	0.889020
H	5.559512	-1.941097	0.001771
I	-2.954814	-0.108626	0.000012

$1^{-•}$

E (PCM(methanol)-[M06-2X/def2-TZVP])= -834.419503826

C	-0.833862	0.045533	0.014886
C	-0.031674	1.213529	-0.615357
C	0.842727	1.269391	0.677178
C	1.835989	0.233211	0.076504
C	0.997722	-0.931553	0.677676
C	0.016943	0.105182	1.311215
C	0.125695	-0.995673	-0.616210
C	0.934812	0.172257	-1.251113
H	1.422821	-1.790192	1.189332
H	-0.468753	2.070291	-1.118559
H	-0.383704	0.078437	2.319416
H	1.143869	2.179517	1.188120

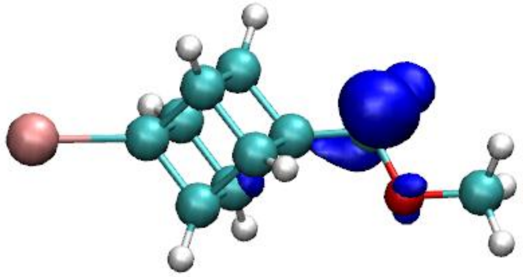

H	1.300582	0.198863	-2.273334
H	-0.186852	-1.903409	-1.122446
C	3.297738	0.368931	0.188075
O	3.897896	1.451065	-0.136092
O	3.892960	-0.870190	-0.206650
C	5.271480	-0.932650	0.050290
H	5.821130	-0.163694	-0.496717
H	5.472226	-0.799903	1.122116
H	5.622436	-1.917319	-0.259254
I	-2.958793	-0.111104	-0.016709

15•

E (PCM(methanol)-[M06-2X/def2-TZVP])= -536.659167544

C	2.626399	-0.320808	0.000431
C	1.915106	0.466606	1.118990
C	1.164723	1.266981	0.005177
C	0.057926	0.183732	-0.000136
C	0.807720	-0.624150	-1.111108
C	1.917484	0.475490	-1.113309
C	1.555446	-1.431185	-0.005150
C	0.805388	-0.632995	1.105611
H	0.329827	-1.046074	-1.989877
H	2.353444	0.897826	2.012967
H	2.357804	0.913811	-2.002850
H	0.981653	2.336837	0.009150
H	0.325641	-1.062027	1.979912
H	1.712240	-2.504659	-0.009289
C	-1.399667	0.440217	-0.000270
O	-1.917271	1.529824	-0.000127
O	-2.091646	-0.702208	-0.000420
C	-3.516120	-0.573266	0.000261
H	-3.844230	-0.036985	0.889123
H	-3.845246	-0.037545	-0.888560
H	-3.906223	-1.585850	0.000818

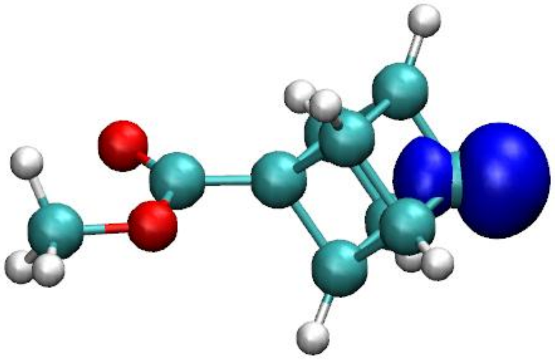

PhS⁻

E (PCM(methanol)-[M06-2X/def2-TZVP])= -629.954663140

C	-0.580831	0.000001	0.000024
C	0.160880	-1.194996	0.000003
C	0.160880	1.194996	0.000003
C	1.548483	-1.195343	0.000002
H	-0.374823	-2.137034	-0.000005
C	1.548485	1.195342	0.000002
H	-0.374820	2.137036	-0.000005
C	2.259241	0.00000	0.000006
H	2.079707	-2.140269	-0.000004

H	2.079709	2.140268	-0.000004
H	3.341416	-0.000002	0.000004
S	-2.333375	0.00000	-0.000014

Ph_2P^-

E (PCM(methanol)-[M06-2X/def2-TZVP])= -804.712954471

P	-0.000070	-1.618637	0.000344
C	-1.439373	-0.501883	-0.016730
C	-2.661345	-0.988224	0.486791
C	-1.476465	0.779453	-0.596968
C	-3.835036	-0.252444	0.414152
H	-2.681974	-1.969664	0.950110
C	-2.644829	1.524702	-0.654964
H	-0.571775	1.192961	-1.026284
C	-3.839219	1.018367	-0.150953
H	-4.751961	-0.669598	0.814639
H	-2.624990	2.508660	-1.109908
H	-4.750492	1.600553	-0.196816
C	1.439422	-0.502055	0.017076
C	1.476762	0.779116	0.597691
C	2.661057	-0.988157	-0.487392
C	2.645130	1.524360	0.655327
H	0.572272	1.192450	1.027597
C	3.834793	-0.252369	-0.415126
H	2.681435	-1.969397	-0.951144
C	3.839262	1.018197	0.150478
H	2.625545	2.508165	1.110609
H	4.751498	-0.669347	-0.816298
H	4.750546	1.600392	0.196044

$8^{\bullet-}$

E (PCM(methanol)-[M06-2X/def2-TZVP])= -1166.61393893

C	-4.573523	1.928233	0.387639
C	-4.096895	1.653551	-0.925148
C	-3.337870	0.545083	-1.197936
C	-2.999992	-0.403081	-0.167073
C	-3.445310	-0.090344	1.167162
C	-4.203075	1.023162	1.421944
S	-1.943806	-1.743388	-0.478954
C	-0.293669	-1.082898	-0.223641
C	0.249220	-0.463727	1.103665
C	1.538779	-1.334856	0.974273
C	2.216019	-0.199134	0.166608
C	1.669391	-0.800588	-1.171616
C	1.000523	-1.947661	-0.356168
C	0.381986	0.066605	-1.040454

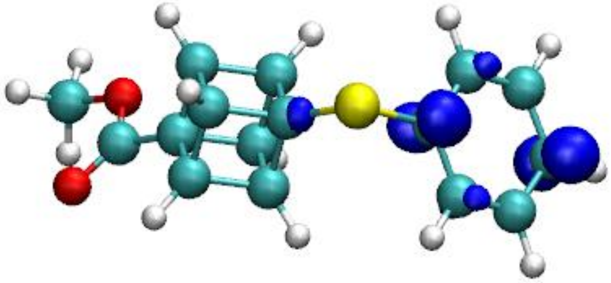

C	0.922087	0.677947	0.284996
H	2.268431	-0.921375	-2.069289
H	-0.338493	-0.326512	2.006522
H	1.018473	-3.001103	-0.620797
H	2.021260	-1.883400	1.777476
H	0.924515	1.732229	0.544712
H	-0.098997	0.619033	-1.842103
H	-2.989104	0.363770	-2.209585
H	-3.179543	-0.761315	1.977779
H	-4.339245	2.334245	-1.735733
H	-4.529214	1.212289	2.440349
H	-5.158298	2.812701	0.599567
C	3.591596	0.304960	0.366722
O	4.366878	-0.085535	1.206167
O	3.889347	1.271455	-0.508215
C	5.197646	1.836065	-0.398739
H	5.330610	2.292255	0.580977
H	5.954146	1.066712	-0.544510
H	5.265073	2.587421	-1.179122

8-TS$^{-\bullet}$

E (PCM(methanol)-[M06-2X/def2-TZVP])= -1166.60040615 Neg-Freq= -270.3

C	-4.608566	1.474490	1.157768
C	-4.241819	1.812749	-0.151198
C	-3.551465	0.926829	-0.952914
C	-3.185859	-0.371195	-0.492539
C	-3.572130	-0.692790	0.840934
C	-4.261991	0.203788	1.632061
S	-2.203483	-1.420149	-1.426290
C	-0.123410	-0.892731	-0.844382
C	0.018940	0.296988	0.140509
C	1.047774	-0.527088	0.979489
C	2.192334	0.044751	0.106624
C	2.050278	-1.146483	-0.900269
C	0.904288	-1.723199	-0.014552
C	1.025876	-0.319901	-1.731904
C	1.169426	0.873889	-0.741611
H	2.898900	-1.700734	-1.295182
H	-0.812147	0.870892	0.547146
H	0.804720	-2.768348	0.272026
H	1.087323	-0.591905	2.064262
H	1.320145	1.918239	-1.005321
H	1.018898	-0.239637	-2.816891
H	-3.271118	1.216609	-1.959276
H	-3.308999	-1.667785	1.235171
H	-4.500084	2.789629	-0.545968

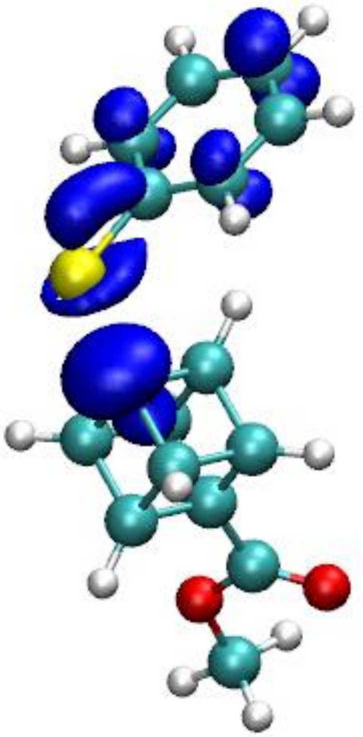

H	-4.535849	-0.085732	2.641036
H	-5.148785	2.173163	1.782273
C	3.483840	0.567661	0.595156
O	3.838264	0.610614	1.749278
O	4.246243	1.004372	-0.415316
C	5.521514	1.538128	-0.055723
H	5.399556	2.394649	0.605575
H	6.122388	0.779493	0.443433
H	5.990649	1.841774	-0.986281

8

E (PCM(methanol)-[M06-2X/def2-TZVP])= -1166.56808560

C	-4.428077	1.950033	0.367756
C	-3.996377	1.678151	-0.924109
C	-3.246435	0.538363	-1.182306
C	-2.923657	-0.333067	-0.145067
C	-3.360882	-0.060848	1.148755
C	-4.110448	1.079868	1.402919
S	-1.949251	-1.787245	-0.470114
C	-0.335650	-1.090930	-0.212103
C	0.204652	-0.462791	1.110811
C	1.494587	-1.332951	0.985372
C	2.169637	-0.203168	0.166977
C	1.622671	-0.817423	-1.164859
C	0.955379	-1.957134	-0.338724
C	0.334909	0.050166	-1.041550
C	0.874018	0.671813	0.279506
H	2.215449	-0.949509	-2.064001
H	-0.378053	-0.318646	2.015334
H	0.970078	-3.011860	-0.593855
H	1.974173	-1.877085	1.792157
H	0.868955	1.727681	0.529726
H	-0.144197	0.595427	-1.848587
H	-2.911615	0.318057	-2.188013
H	-3.115952	-0.745577	1.950782
H	-4.244831	2.351663	-1.734118
H	-4.448730	1.286480	2.410127
H	-5.014120	2.837708	0.568098
C	3.545687	0.304829	0.364654
O	4.314131	-0.073272	1.214846
O	3.845285	1.255394	-0.524777
C	5.154067	1.821950	-0.421429
H	5.286083	2.289063	0.553112
H	5.909947	1.050505	-0.558459
H	5.221008	2.563870	-1.210643

10⁻•

E (PCM(methanol)-[M06-2X/def2-TZVP])= -1341.41737877

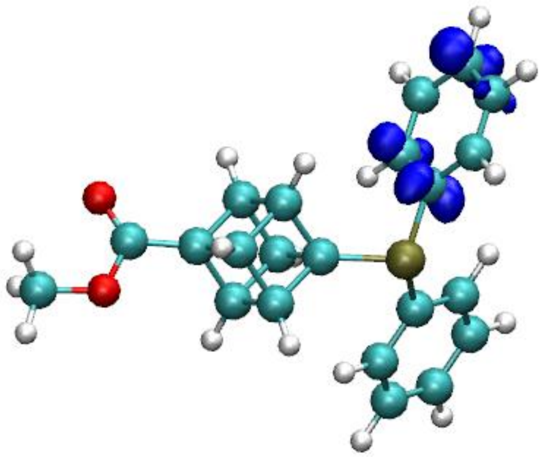

C	-1.557154	0.992353	-0.798798
C	-1.479392	-0.615304	1.379781
C	-2.790167	0.338356	-0.122856
C	-1.006475	1.392934	0.602951
C	-2.240212	0.737585	1.291674
C	-0.228295	0.039899	0.691497
C	-2.031967	-1.025529	-0.016977
C	-0.801626	-0.369219	-0.710496
H	-1.605526	1.639854	-1.669320
H	-1.442303	-1.255328	2.257614
H	-0.578018	2.358023	0.860313
H	-2.851109	1.180726	2.072664
H	-2.473896	-1.985009	-0.270391
H	-0.223221	-0.825537	-1.509290
P	1.513304	-0.043802	1.307739
C	1.960955	-1.597519	0.393627
C	3.186179	-1.713719	-0.261898
C	1.112350	-2.707540	0.404711
C	3.554721	-2.899297	-0.887166
H	3.848593	-0.855072	-0.285198
C	1.478690	-3.897199	-0.212172
H	0.148263	-2.644688	0.900494
C	2.703405	-3.997328	-0.862482
H	4.507363	-2.965408	-1.399077
H	0.807352	-4.747159	-0.187894
H	2.990141	-4.922123	-1.346972
C	2.332416	1.260622	0.448001
C	2.154050	1.497496	-0.971669
C	3.190176	2.163894	1.137484
C	2.802667	2.548435	-1.594421
H	1.513894	0.841012	-1.550726
C	3.827166	3.202331	0.506648
H	3.342266	2.018818	2.204308
C	3.646714	3.420048	-0.899048
H	2.648452	2.699729	-2.659578
H	4.470569	3.860792	1.079690
H	4.144572	4.236431	-1.405241
C	-4.201534	0.507875	-0.528076
O	-4.603456	1.268269	-1.375782
O	-5.011563	-0.284801	0.182994
C	-6.407460	-0.175190	-0.102509
H	-6.753429	0.839102	0.090760
H	-6.601820	-0.431005	-1.142899
H	-6.902782	-0.877788	0.560117

10-TS⁻•

E (PCM(methanol)-[M06-2X/def2-TZVP])= -1341.37921878. Neg-Freq= -135.27

C	1.329391	-0.654727	-0.623631
C	1.595702	0.511120	1.825587
C	2.661243	-0.217475	0.038342
C	0.883578	-1.295650	0.728783
C	2.220780	-0.852091	1.402432
C	0.275198	0.067936	1.136999
C	2.036968	1.147987	0.473292
C	0.697356	0.713303	-0.202061
H	1.251402	-1.119387	-1.603436
H	1.708687	0.973193	2.802411
H	0.423047	-2.274888	0.831428
H	2.868616	-1.475307	2.013661
H	2.532394	2.107603	0.349980
H	0.085132	1.351198	-0.836285
P	-2.853239	0.184953	1.381910
C	-2.462687	1.590894	0.294642
C	-2.791569	1.679592	-1.067452
C	-1.840268	2.716011	0.861919
C	-2.482168	2.802617	-1.822969
H	-3.301290	0.850954	-1.545172
C	-1.548568	3.848646	0.116600
H	-1.558381	2.685349	1.909484
C	-1.853990	3.898392	-1.240042
H	-2.742412	2.825402	-2.875180
H	-1.062131	4.692026	0.593158
H	-1.610972	4.773629	-1.828892
C	-2.699659	-1.277770	0.321958
C	-1.933016	-1.373788	-0.853764
C	-3.294353	-2.472781	0.774529
C	-1.782573	-2.574440	-1.532533
H	-1.430407	-0.493029	-1.233584
C	-3.133329	-3.675598	0.105498
H	-3.896288	-2.448211	1.677759
C	-2.378393	-3.740287	-1.063011
H	-1.178753	-2.601069	-2.433091
H	-3.607324	-4.569803	0.493802
H	-2.257928	-4.676672	-1.592214
C	4.014925	-0.391697	-0.526720
O	4.283559	-0.988126	-1.541798
O	4.942268	0.186169	0.245726
C	6.296832	0.046974	-0.187224
H	6.574609	-1.005284	-0.225645
H	6.426210	0.491109	-1.172836

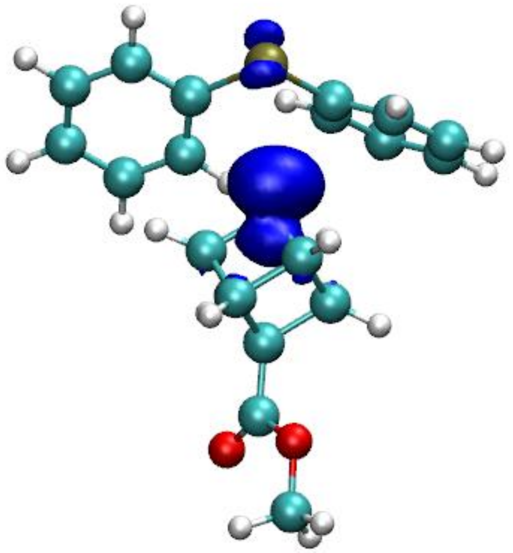

H	6.899352	0.571537	0.547521

10

E (PCM(methanol)-[M06-2X/def2-TZVP])= -1341.36694700

C	-1.704124	1.154179	-0.411943
C	-1.347063	-1.063010	1.108655
C	-2.809806	0.132311	-0.040442
C	-1.204301	1.139601	1.062857
C	-2.308369	0.111577	1.445574
C	-0.225164	-0.035560	0.723384
C	-1.846278	-1.054852	-0.366643
C	-0.743020	-0.023848	-0.756658
H	-1.864219	2.041346	-1.016430
H	-1.196024	-1.948487	1.718813
H	-0.933770	2.016089	1.644489
H	-2.963748	0.170098	2.308502
H	-2.131543	-1.929405	-0.943383
H	-0.115427	-0.083162	-1.641048
P	1.485362	-0.015542	1.340293
C	2.244140	-1.419296	0.443453
C	3.633199	-1.561940	0.500937
C	1.501859	-2.383676	-0.235758
C	4.264224	-2.629047	-0.119069
H	4.226012	-0.825764	1.033557
C	2.132968	-3.462342	-0.846979
H	0.423365	-2.307501	-0.296857
C	3.513356	-3.585448	-0.794403
H	5.342026	-2.719373	-0.070787
H	1.541216	-4.203200	-1.369508
H	4.003909	-4.422352	-1.274466
C	2.077403	1.421356	0.356900
C	2.456947	1.330150	-0.983177
C	2.082350	2.672511	0.973468
C	2.827020	2.466265	-1.689383
H	2.470599	0.365249	-1.476385
C	2.443179	3.811997	0.264611
H	1.804886	2.755843	2.018511
C	2.817284	3.709508	-1.068302
H	3.122470	2.381576	-2.727586
H	2.439702	4.776291	0.756605
H	3.105706	4.594267	-1.621360
C	-4.235134	0.212258	-0.431061
O	-4.752328	1.142221	-1.000528
O	-4.903675	-0.882893	-0.058483
C	-6.300305	-0.901440	-0.364574
H	-6.806147	-0.076349	0.134145

H	-6.450790	-0.821713	-1.439900
H	-6.672810	-1.852462	0.002110

16-reduction

E (PCM(methanol)-[M06-2X/def2-TZVP])= -537.333453462

C	2.650969	-0.296539	0.000799
C	1.873122	0.482026	1.107059
C	1.102641	1.278086	0.007267
C	-0.002119	0.191251	-0.000406
C	0.771000	-0.605314	-1.106095
C	1.876642	0.494566	-1.098908
C	1.541754	-1.394893	-0.007235
C	0.767542	-0.617897	1.098057
H	0.300448	-1.032759	-1.986057
H	2.309868	0.915533	2.000995
H	2.316338	0.938166	-1.986425
H	0.910334	2.346404	0.012853
H	0.294162	-1.055459	1.971503
H	1.715788	-2.465951	-0.013055
C	-1.461302	0.432916	-0.000550
O	-1.993592	1.516565	-0.000107
O	-2.145123	-0.716471	-0.000753
C	-3.569748	-0.599698	0.000523
H	-3.903066	-0.066191	0.889275
H	-3.904873	-0.066771	-0.887886
H	-3.952087	-1.615371	0.001272
H	3.719796	-0.485381	0.001334

16-reduction-TS

E (PCM(methanol)-[M06-2X/def2-TZVP])= -593.192486780, Neg.Freq=-1693.1

C	1.295859	0.599476	-1.215033
C	0.353078	-0.441733	1.103901
C	2.137167	-0.063992	-0.089051
C	0.277960	-0.577493	-1.101397
C	1.133515	-1.243346	0.018649
C	-0.503076	0.237236	-0.020664
C	1.373874	0.733442	1.000079
C	0.516061	1.399535	-0.122736
H	1.673524	1.010744	-2.145216
H	-0.059159	-0.848250	2.022031
H	-0.191056	-1.094171	-1.932753
H	1.384401	-2.297427	0.074125
H	1.815627	1.253160	1.844009
H	0.249161	2.450222	-0.175871
H	3.389337	-0.194380	-0.142174
N	4.698599	-0.385662	0.028820

H	4.829624	0.241887	0.826907
H	4.649754	-1.314656	0.456055
C	-1.975884	0.373674	0.031124
O	-2.581090	1.415273	0.099466
O	-2.572039	-0.821415	0.001044
C	-4.000853	-0.814166	0.055584
H	-4.340810	-0.350463	0.980213
H	-4.406871	-0.268370	-0.794605
H	-4.304909	-1.855322	0.018725

NH_3

E (PCM(methanol)-[M06-2X/def2-TZVP])= -56.5551042794

N	0.00000	0.00000	0.115302
H	0.00000	0.938000	-0.269038
H	-0.812332	-0.469000	-0.269038
H	0.812332	-0.469000	-0.269038

$NH_2^{\bullet}$

E (PCM(methanol)-[M06-2X/def2-TZVP])= -55.8743600454

N	0.00000	0.00000	0.141016
H	0.00000	0.804431	-0.493556
H	0.00000	-0.804431	-0.493556

2

E (PCM(methanol)-[M06-2X/def2-TZVP])= -903.519254608

C	-0.445663	-0.209862	1.263676
C	0.445679	0.209956	-1.263662
C	-1.325956	0.000423	0.000065
C	0.444880	-1.199292	0.449767
C	-0.445666	-0.988949	-0.813539
C	1.325962	-0.000386	-0.000066
C	-0.444909	1.199366	-0.449781
C	0.445685	0.989018	0.813489
H	-0.821170	-0.376448	2.267170
H	0.821238	0.376565	-2.267137
H	0.819883	-2.152323	0.806327
H	-0.821604	-1.774916	-1.459173
H	-0.819906	2.152397	-0.806337
H	0.821670	1.774992	1.459096
I	3.435168	-0.000040	0.000033
I	-3.435171	0.000004	-0.000026

17

E (PCM(methanol)-[M06-2X/def2-TZVP])= -605.806842164

C	-2.142416	-0.866181	0.955982
C	-1.257694	0.863326	-0.952827

C	-2.988006	-0.000773	-0.000479
C	-1.256712	-1.257180	-0.270899
C	-2.142111	-0.395187	-1.228185
C	-0.387569	0.000219	0.000350
C	-2.143578	1.260937	0.271667
C	-1.258022	0.393923	1.224108
H	-2.492275	-1.555197	1.717194
H	-0.878889	1.545969	-1.706240
H	-0.877414	-2.250794	-0.485088
H	-2.491137	-0.709386	-2.205949
H	-2.494371	2.264333	0.488027
H	-0.880198	0.705375	2.192305
I	1.727753	0.000098	0.000027

13b$^{-\bullet}$

E (PCM(methanol)-[M06-2X/def2-TZVP])= -1235.76278131

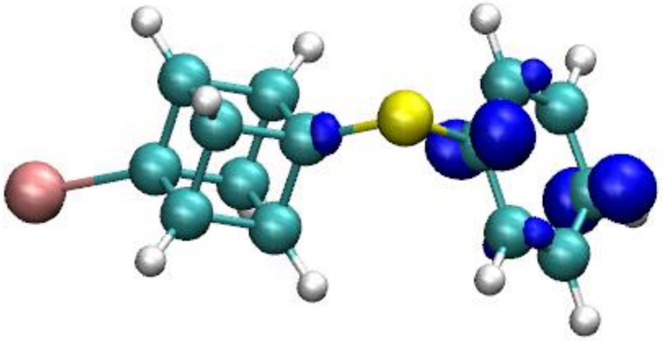

C	-4.927640	-2.031408	0.000188
C	-4.550439	-1.396082	1.216166
C	-3.872330	-0.204677	1.225884
C	-3.528479	0.471384	0.000081
C	-3.871794	-0.205085	-1.225650
C	-4.549889	-1.396497	-1.215833
S	-2.491245	1.863589	0.000075
C	-0.836234	1.165881	0.000030
C	-0.238415	0.238881	-1.106933
C	1.061298	1.102068	-1.109788
C	1.639296	0.183736	-0.000051
C	1.061275	1.101837	1.109866
C	0.469813	2.024561	0.000129
C	-0.238438	0.238650	1.106809
C	0.353281	-0.686337	-0.000154
I	3.618784	-0.590014	-0.000121
H	1.603022	1.433877	1.989493
H	-0.775372	-0.099694	-1.987612
H	0.504364	3.109747	0.000243
H	1.603063	1.434291	-1.989334
H	0.327882	-1.770981	-0.000269
H	-0.775414	-0.100110	1.987405
H	-3.595689	0.252850	2.170427
H	-3.594739	0.252131	-2.170224
H	-4.804536	-1.861474	2.163789
H	-4.803559	-1.862209	-2.163414
H	-5.452495	-2.976601	0.000231

13b$^{\bullet-}$**-TS**

E (PCM(methanol)-[M06-2X/def2-TZVP])= -1235.74940822. Neg-Freq= -246.9

C	0.604475	-0.119185	1.028537
C	0.431485	1.676301	-1.002728
C	1.714371	0.232261	0.002780
C	0.324270	1.406579	1.199850
C	1.449771	1.750217	0.176305
C	-0.698212	1.334567	0.020790
C	0.711377	0.151323	-1.178926
C	-0.415346	-0.182456	-0.151091
H	0.704702	-0.848639	1.827215
H	0.370243	2.395796	-1.816076
H	0.177016	1.909214	2.153028
H	2.221149	2.504140	0.306076
H	0.895656	-0.363361	-2.117730
H	-1.177067	-0.948796	-0.279136
C	-3.682780	0.422658	-0.008310
C	-3.981424	-0.262871	1.204762
C	-4.003869	-0.268592	-1.212386
C	-4.551686	-1.520075	1.205149
H	-3.747743	0.223961	2.144731
C	-4.574996	-1.524259	-1.197043
H	-3.786011	0.212833	-2.159014
C	-4.857900	-2.179338	0.008942
H	-4.761791	-2.002826	2.153663
H	-4.803851	-2.011181	-2.138965
H	-5.302170	-3.165512	0.015552
S	-2.838992	1.913609	-0.016955
I	3.656915	-0.642003	-0.007506

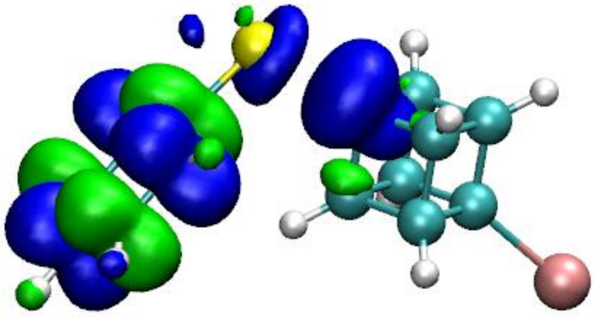

13b

E (PCM(methanol)-[M06-2X/def2-TZVP])= -1235.71640352

C	-4.885132	-1.989128	0.000061
C	-4.524495	-1.395369	1.202779
C	-3.800559	-0.210429	1.204923
C	-3.432469	0.382738	0.000038
C	-3.800323	-0.210599	-1.204837
C	-4.524251	-1.395543	-1.202670
S	-2.474991	1.883565	0.000024
C	-0.856914	1.150647	-0.000007
C	-0.258373	0.228939	-1.108883
C	1.036751	1.098200	-1.109505
C	1.620943	0.181762	-0.000020
C	1.036749	1.098169	1.109492
C	0.440568	2.017084	0.000006
C	-0.258375	0.228909	1.108841
C	0.336075	-0.691834	-0.000033

I	3.595972	-0.584418	-0.000031
H	1.572867	1.436205	1.989441
H	-0.789831	-0.111785	-1.991757
H	0.463418	3.101697	0.000020
H	1.572870	1.436261	-1.989444
H	0.310635	-1.775974	-0.000049
H	-0.789843	-0.111838	1.991701
H	-3.520066	0.259445	2.139070
H	-3.519655	0.259150	-2.138995
H	-4.808487	-1.852868	2.141744
H	-4.808048	-1.853180	-2.141625
H	-5.451678	-2.911274	0.000070

13d

E (PCM(methanol)-[M06-2X/def2-TZVP])= -1410.51551002

C	3.047426	-3.804228	-1.136560
C	3.073719	-2.559377	-1.754270
C	2.786077	-1.413226	-1.026488
C	2.473632	-1.496071	0.331397
C	2.458943	-2.749327	0.943154
C	2.737799	-3.898604	0.213235
C	0.273901	0.092463	0.794659
C	-0.758540	-1.029421	1.156941
C	-1.780929	0.054862	1.609984
C	-2.324603	0.107007	0.156510
C	-1.319982	1.232910	-0.211371
C	-0.764883	1.177787	1.245673
C	-0.301200	0.141714	-0.663485
C	-1.314512	-0.983314	-0.296678
I	-4.382224	0.127402	-0.350154
H	-1.565065	2.135862	-0.761095
H	-0.517732	-1.934680	1.706338
H	-0.537315	2.041729	1.862549
H	-2.390966	0.017853	2.505949
H	-1.553834	-1.845987	-0.909120
H	0.291036	0.177163	-1.572796
H	2.814048	-0.446852	-1.516407
H	2.232464	-2.826735	2.000826
H	3.318656	-2.481362	-2.806029
H	2.721628	-4.864414	0.701972
H	3.274553	-4.696849	-1.705282
P	2.000669	-0.038334	1.347357
C	2.811083	1.325747	0.434477
C	2.107015	2.352324	-0.192442
C	4.207819	1.368956	0.424483
C	2.782936	3.393840	-0.820096

H	1.024299	2.355089	-0.199128
C	4.882296	2.399269	-0.211640
H	4.771962	0.583466	0.916271
C	4.169417	3.417825	-0.835572
H	2.220938	4.183566	-1.302483
H	5.964708	2.412093	-0.216373
H	4.693972	4.225960	-1.328881

13d•–

E (PCM(methanol)-[M06-2X/def2-TZVP])= E=-1123.9904298

C	0.591041780680	0.280063216859	-0.346703968358
C	0.832990251243	0.005053769053	1.007311216925
C	2.115780280099	-0.001147507851	1.542414963775
C	3.258218410717	0.286669619490	0.693379427027
C	2.997709641957	0.557914564658	-0.678979797414
C	1.720731077962	0.560416634217	-1.197159554054
C	4.957651308741	-1.277180526702	2.429189473696
C	4.338494722321	-2.651859855339	1.979303190317
C	5.626629777202	-3.403372414789	2.481724019273
C	4.958005794252	-3.547141936209	3.893553039542
C	5.586128693785	-2.187897116969	4.363121079646
C	6.246723296041	-2.034212207483	2.944285648228
C	4.297856478943	-1.446472997927	3.852746795046
C	3.666517926907	-2.810628401083	3.390621970776
I	4.950294641849	-5.341074182291	5.054726084197
H	6.078626056533	-2.022701406232	5.313458913116
H	3.844178838925	-2.813340668208	1.028570390362
H	7.268173376641	-1.721598478955	2.759221202070
H	6.149476320066	-4.193607578824	1.957632276658
H	2.650536558209	-3.136295796762	3.574126758423
H	3.780103751159	-0.662077946453	4.392066327184
H	2.265815412872	-0.208617360458	2.594959058358
H	3.838888016332	0.768592323460	-1.333045251812
H	-0.006946993445	-0.212180964196	1.659763222808
H	1.565139088489	0.771657132220	-2.247894392796
H	-0.414163510151	0.277476430138	-0.744550639963
P	4.958227774208	0.245101118986	1.316746214086
C	4.997327747262	1.485158859739	2.750736315937
C	5.983627417392	1.363078511424	3.735419697697
C	4.097110564500	2.548247851449	2.825720897558
C	6.074149937720	2.290655931248	4.771679879452
H	6.686713969258	0.538933942009	3.694713217754
C	4.180072956042	3.475283547884	3.866147868456
H	3.328315497563	2.638912908759	2.068167729613
C	5.170010119397	3.350347320843	4.840208745248
H	6.845299603958	2.186822778353	5.523003910991

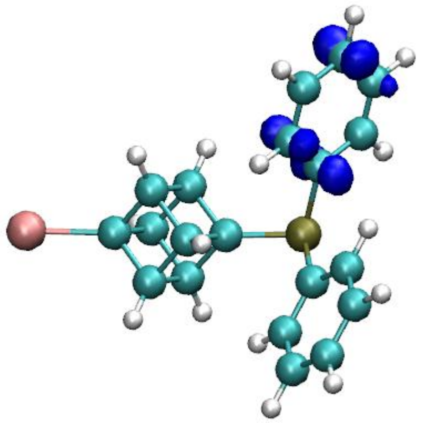

H	3.471267317632	4.290579174178	3.918960556834
H	5.236946786741	4.070846371765	5.643261213344

14b•

E (PCM(methanol)-[M06-2X/def2-TZVP])= -938.002171856

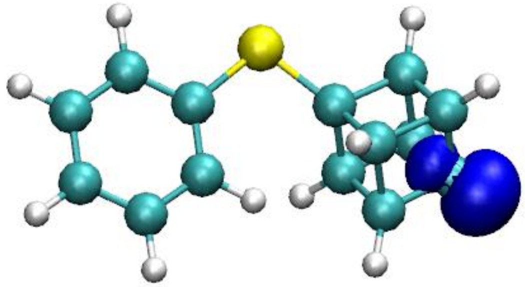

C	2.970692	-0.047820	-1.118146
C	1.531427	0.559140	1.116788
C	3.334317	0.949579	0.000059
C	2.567875	-1.058770	0.000139
C	2.970577	-0.047687	1.118345
C	1.128200	-0.458023	0.000029
C	1.927823	1.579595	-0.000049
C	1.531542	0.559009	-1.116808
H	3.548139	-0.293621	-2.003003
H	0.940128	0.786439	1.998864
H	2.788174	-2.122256	0.000212
H	3.547935	-0.293383	2.003289
H	1.673791	2.635120	-0.000124
H	0.940336	0.786205	-1.998973
C	-1.646076	-0.517844	-0.000021
C	-2.897525	-1.137163	0.000012
C	-1.571126	0.872852	-0.000106
C	-4.053771	-0.374400	-0.000039
H	-2.961596	-2.218763	0.000078
C	-2.737771	1.629187	-0.000151
H	-0.616560	1.380179	-0.000143
C	-3.980553	1.014297	-0.000119
H	-5.016888	-0.868723	-0.000012
H	-2.665924	2.709412	-0.000216
H	-4.884352	1.608971	-0.000157
S	-0.234186	-1.577581	0.000037

14d•

E (PCM(methanol)-[M06-2X/def2-TZVP])= -1112.79997302

C	2.805981	-0.726842	0.936382
C	1.325576	-2.436001	-0.582312
C	3.028084	-2.239082	0.724551
C	2.583411	-0.609366	-0.602270
C	2.840475	-2.134435	-0.802776
C	1.060578	-0.903878	-0.390618
C	1.539883	-2.565361	0.959959
C	1.292225	-1.035063	1.153509
H	3.399265	-0.043439	1.535726
H	0.720534	-3.102754	-1.190809
H	2.976068	0.183892	-1.233311
H	3.461380	-2.569022	-1.579593

H	1.123666	-3.349935	1.585111
H	0.665618	-0.592634	1.923436
P	-0.175863	0.077618	-1.289097
C	-1.749965	-0.477880	-0.536899
C	-2.913751	0.215247	-0.880544
C	-1.853211	-1.574230	0.317096
C	-4.144336	-0.163590	-0.367760
H	-2.853333	1.065402	-1.551910
C	-3.089603	-1.963251	0.822259
H	-0.972828	-2.137476	0.599801
C	-4.235558	-1.257526	0.486423
H	-5.034093	0.390799	-0.637950
H	-3.151854	-2.817484	1.484521
H	-5.196066	-1.558809	0.884170
C	0.098768	1.676417	-0.421504
C	-0.452472	1.970843	0.825934
C	0.943569	2.610433	-1.021487
C	-0.164588	3.172205	1.458471
H	-1.116344	1.259588	1.303289
C	1.240981	3.809170	-0.384181
H	1.368416	2.401237	-1.997174
C	0.684542	4.092684	0.855474
H	-0.602787	3.389467	2.424462
H	1.899565	4.524061	-0.860819
H	0.907215	5.029870	1.349275

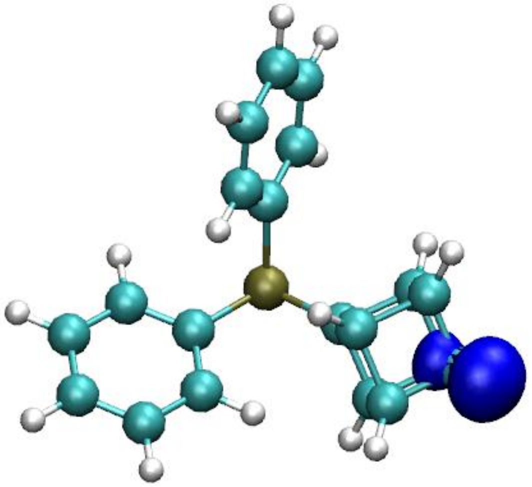

12b$^{-\bullet}$

E (PCM(methanol)-[M06-2X/def2-TZVP])= -1567.95513041

C	0.337040	1.329061	-0.000306
C	-0.423071	-1.269607	0.000199
C	0.692781	0.292711	1.107123
C	0.692747	0.292296	-1.107355
C	-0.776633	-0.234105	-1.109244
C	-0.776603	-0.233682	1.109255
H	0.616465	2.378582	-0.000507
H	-0.712307	-2.316911	0.000402
H	1.300457	0.493530	1.984552
H	1.300396	0.492789	-1.984877
H	-1.384356	-0.431008	-1.988000
H	-1.384297	-0.430253	1.988104
C	1.050284	-0.750650	0.000073
C	-1.131446	0.804619	-0.000190
S	2.470901	-1.847362	0.000245
C	3.842569	-0.785868	0.000157
C	4.398041	-0.271121	1.226219
C	4.398044	-0.271336	-1.225994

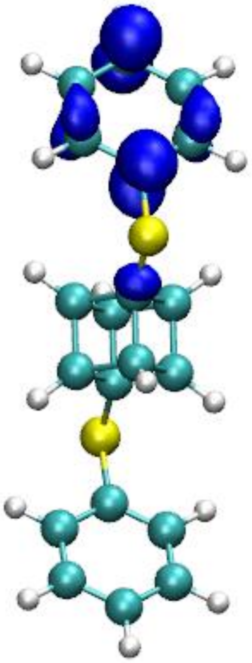

C	5.440396	0.619176	1.216756
H	3.985681	-0.612098	2.170532
C	5.440395	0.618966	-1.216684
H	3.985681	-0.612472	-2.170247
C	6.009827	1.090600	-0.000005
H	5.837366	0.970938	2.164376
H	5.837362	0.970569	-2.164365
H	6.826610	1.798939	-0.000066
S	-2.517484	1.918951	-0.000407
C	-3.830352	0.716449	-0.000151
C	-4.343436	0.241261	1.204608
C	-4.342752	0.240007	-1.204703
C	-5.357593	-0.707046	1.202872
H	-3.948104	0.619647	2.138833
C	-5.356914	-0.708293	-1.202551
H	-3.946850	0.617378	-2.139097
C	-5.864564	-1.183045	0.000264
H	-5.752647	-1.072498	2.142113
H	-5.751417	-1.074745	-2.141635
H	-6.657256	-1.920163	0.000421

12d$^{-\bullet}$

E (PCM(methanol)-[M06-2X/def2-TZVP])= -1917.55528892

C	-0.760397	-0.635645	0.111865
C	0.546892	0.554422	-1.941231
C	-1.419616	0.285432	-0.971630
C	0.020916	-1.374357	-1.012884
C	-0.622743	-0.459524	-2.098579
C	1.212547	-0.368216	-0.862312
C	-0.232769	1.296413	-0.817759
C	0.405331	0.380458	0.267748
H	-1.292430	-1.116146	0.928897
H	1.085977	1.034353	-2.754058
H	0.144660	-2.453001	-1.085725
H	-1.052683	-0.795702	-3.038702
H	-0.359642	2.373956	-0.739828
H	0.807342	0.720436	1.217399
P	2.937813	-0.975735	-0.981361
C	3.830890	0.646420	-1.041959
C	5.150809	0.638157	-1.503521
C	3.302330	1.865148	-0.607460
C	5.916681	1.794232	-1.530809
H	5.581813	-0.297089	-1.847981
C	4.065707	3.029095	-0.632088
H	2.282662	1.917608	-0.244689
C	5.374268	2.999468	-1.092808

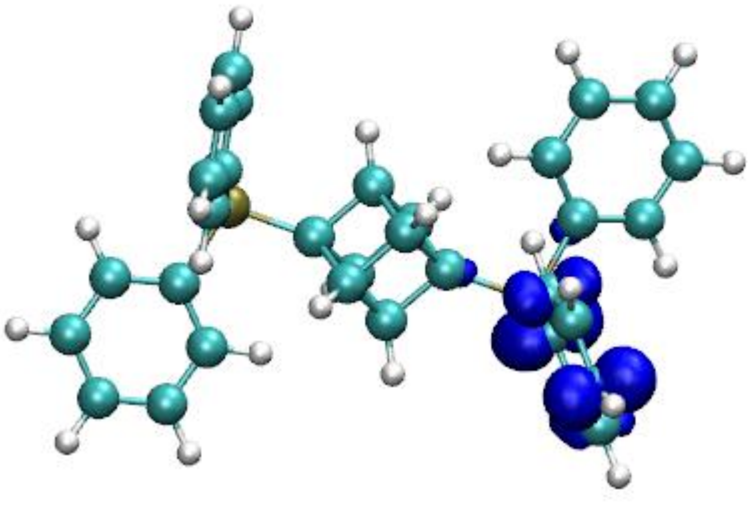

H	6.934693	1.759517	-1.899332
H	3.634806	3.961322	-0.286464
H	5.967765	3.904754	-1.111319
C	3.326370	-1.636265	0.616376
C	3.290284	-0.851963	1.832941
C	3.855918	-2.960532	0.741992
C	3.718346	-1.377873	3.031481
H	2.937898	0.172724	1.794319
C	4.269730	-3.473595	1.944104
H	3.924182	-3.577272	-0.150371
C	4.205960	-2.692959	3.137825
H	3.677140	-0.754429	3.920395
H	4.650560	-4.488774	1.983861
H	4.534865	-3.093562	4.087084
P	-3.127594	0.891868	-1.088003
C	-4.106516	-0.645762	-0.917042
C	-3.554528	-1.917801	-1.060101
C	-5.481994	-0.534090	-0.697385
C	-4.358127	-3.050742	-0.980566
C	-6.281242	-1.662599	-0.604496
C	-5.720034	-2.927316	-0.748755
H	-2.492378	-2.039839	-1.234142
H	-5.929880	0.448336	-0.590264
H	-3.913374	-4.031270	-1.095461
H	-7.343741	-1.556766	-0.425328
H	-6.343286	-3.809913	-0.683880
C	-3.260790	1.606732	0.601357
C	-3.045516	2.977562	0.743921
C	-3.495112	0.840992	1.744491
C	-3.050920	3.570619	2.000536
C	-3.511342	1.433709	2.999419
C	-3.285759	2.799135	3.130261
H	-2.874470	3.586712	-0.136851
H	-3.671980	-0.224243	1.652373
H	-2.879917	4.635442	2.095209
H	-3.698727	0.828785	3.877743
H	-3.298608	3.259522	4.109960

12b•–-TS

E (PCM(methanol)-[M06-2X/def2-TZVP])= -1567.94122128 Neg-Freq=-265.7

C	-0.700762	-2.853041	0.144067
C	0.379469	-0.387849	-0.127337
C	0.156068	-2.379857	-1.069639
C	0.305721	-2.201127	1.140325
C	-0.482270	-0.854656	1.086754
C	-0.631280	-1.033596	-1.125618

H	-1.170578	-3.828207	0.255066
H	0.832149	0.596242	-0.236426
H	0.413595	-2.993564	-1.930440
H	0.683086	-2.672950	2.045438
H	-0.776036	-0.243285	1.937541
H	-1.041994	-0.562290	-2.016158
C	1.163520	-1.725770	-0.072689
C	-1.486219	-1.508291	0.088638
S	3.381209	-1.563935	-0.228103
C	3.705122	0.112759	-0.083272
C	3.823989	0.746228	1.187579
C	3.746234	0.975119	-1.216940
C	3.978000	2.113385	1.304731
H	3.791485	0.129470	2.078523
C	3.901970	2.339523	-1.085273
H	3.648654	0.536366	-2.203660
C	4.018596	2.939612	0.175787
H	4.067753	2.550657	2.293565
H	3.930154	2.955339	-1.977897
H	4.137642	4.010320	0.273975
S	-3.261739	-1.474490	0.204581
C	-3.510654	0.281751	0.062987
C	-3.672194	0.866387	-1.191067
C	-3.533616	1.080344	1.204310
C	-3.850447	2.238976	-1.301543
H	-3.661119	0.242206	-2.075535
C	-3.713261	2.452611	1.090294
H	-3.412723	0.622258	2.177918
C	-3.870624	3.033247	-0.161836
H	-3.975917	2.687388	-2.278824
H	-3.730203	3.067441	1.981036
H	-4.011649	4.102885	-0.249307

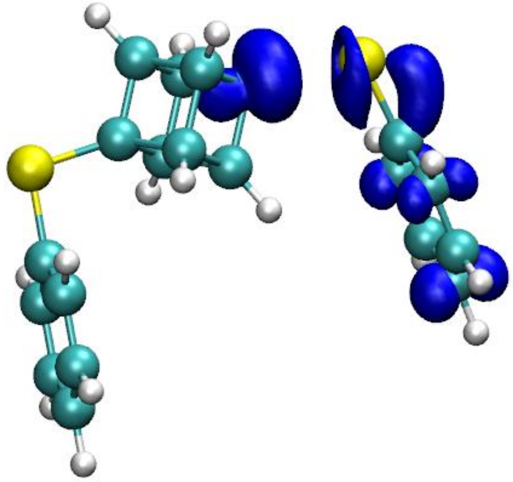

12d$^{\bullet-}$-TS

E (PCM(methanol)-[M06-2X/def2-TZVP])= -1917.51809151 Neg-Freq= -135.1

C	0.647462	-0.866950	0.542411
C	-0.369953	0.807329	2.416364
C	1.440024	-0.066899	1.602084
C	-0.361703	-1.272724	1.661828
C	0.452159	-0.478846	2.727774
C	-1.199788	0.012555	1.349860
C	0.639042	1.223104	1.299686
C	-0.175125	0.426929	0.235870
H	1.063041	-1.546180	-0.200267
H	-0.805339	1.475077	3.157373
H	-0.791575	-2.263469	1.794737

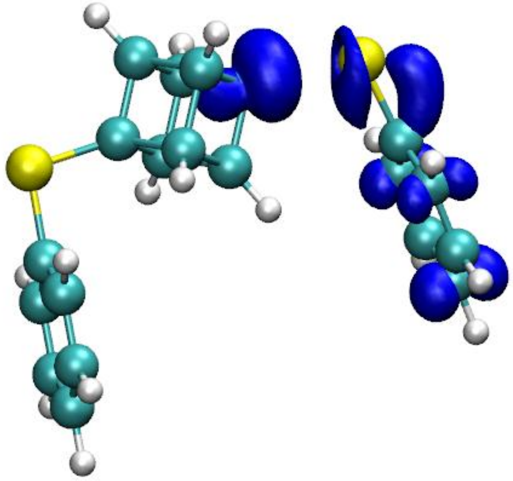

H	0.699159	-0.839120	3.722888
H	1.042360	2.222643	1.155042
H	-0.444715	0.793920	-0.752809
P	-3.015095	-0.004242	1.344578
C	-3.373848	1.461157	0.296293
C	-4.493429	1.492951	-0.535542
C	-2.571825	2.600880	0.382475
C	-4.799662	2.631260	-1.269071
H	-5.128682	0.618432	-0.618353
C	-2.878238	3.739476	-0.351926
H	-1.695254	2.605096	1.021145
C	-3.993059	3.758493	-1.180446
H	-5.670133	2.635584	-1.913069
H	-2.240481	4.611626	-0.279534
H	-4.231143	4.645122	-1.753790
C	-3.401642	-1.372787	0.186298
C	-2.523141	-1.814838	-0.805244
C	-4.634094	-2.010552	0.322909
C	-2.874884	-2.866807	-1.639649
H	-1.557881	-1.338959	-0.930509
C	-4.995426	-3.051950	-0.523207
H	-5.317404	-1.689764	1.101411
C	-4.113653	-3.483722	-1.503921
H	-2.181674	-3.205279	-2.399509
H	-5.959433	-3.530896	-0.407747
H	-4.386493	-4.302232	-2.157627
P	4.474548	-0.005980	0.784112
C	3.861259	-1.491345	-0.067174
C	3.568152	-2.626125	0.708749
C	3.714568	-1.629812	-1.456715
C	3.140083	-3.815498	0.138477
C	3.266264	-2.811476	-2.030633
C	2.970448	-3.916678	-1.238911
H	3.657038	-2.557890	1.788257
H	3.959737	-0.793554	-2.100983
H	2.918120	-4.664935	0.774312
H	3.156131	-2.872882	-3.107418
H	2.619764	-4.838148	-1.685829
C	3.864094	1.383007	-0.207143
C	4.483292	2.635677	-0.021493
C	2.741156	1.366195	-1.054585
C	4.014133	3.787379	-0.632606
C	2.279248	2.515538	-1.679001
C	2.907660	3.740130	-1.477244
H	5.353462	2.697602	0.625057
H	2.203207	0.438013	-1.205370

H	4.520245	4.729632	-0.456186
H	1.404938	2.457087	-2.318356
H	2.543760	4.636506	-1.962617

14b

E (PCM(methanol)-[M06-2X/def2-TZVP])= -938.676890058

C	2.936836	-0.091751	-1.104579
C	1.499903	0.514140	1.111328
C	3.360094	0.925133	0.000002
C	2.514370	-1.106057	0.000046
C	2.936807	-0.091691	1.104628
C	1.075661	-0.501508	0.000011
C	1.920546	1.526763	-0.000033
C	1.499932	0.514080	-1.111350
H	3.515180	-0.339769	-1.988582
H	0.912992	0.747980	1.994583
H	2.725962	-2.171183	0.000078
H	3.515128	-0.339661	1.988660
H	1.680210	2.585679	-0.000065
H	0.913044	0.747871	-1.994633
C	-1.700977	-0.514301	0.000001
C	-2.965613	-1.106936	0.000007
C	-1.598059	0.875081	-0.000025
C	-4.106025	-0.320662	-0.000012
H	-3.052119	-2.186998	0.000027
C	-2.748992	1.655230	-0.000043
H	-0.632475	1.361413	-0.000031
C	-4.004457	1.066230	-0.000037
H	-5.079263	-0.794883	-0.000007
H	-2.654910	2.733818	-0.000062
H	-4.895966	1.679260	-0.000052
S	-0.309078	-1.599439	0.000026
H	4.277308	1.505000	-0.000002

14b-TS

E (PCM(methanol)-[M06-2X/def2-TZVP])= -994.535682288. Neg-Freq: -1689.01

C	2.500108	-0.578280	-1.053701
C	1.083875	0.167710	1.140966
C	2.990681	0.338615	0.096354
C	1.915057	-1.550929	0.015159
C	2.422784	-0.632676	1.167943
C	0.572345	-0.755949	-0.012011
C	1.664362	1.143816	0.068530
C	1.159570	0.219249	-1.085200
H	3.073909	-0.878073	-1.924321

H	0.502125	0.457413	2.010857
H	1.985135	-2.634204	-0.008656
H	2.933392	-0.973297	2.062368
H	1.568658	2.224881	0.091805
H	0.638789	0.547225	-1.979845
C	-2.177789	-0.402256	-0.033622
C	-3.509956	-0.820586	-0.011443
C	-1.888924	0.960593	-0.029391
C	-4.533535	0.112278	0.012806
H	-3.740791	-1.879317	-0.012193
C	-2.923482	1.888455	-0.003766
H	-0.867222	1.313361	-0.047848
C	-4.246338	1.473197	0.016829
H	-5.561501	-0.226828	0.029891
H	-2.685428	2.944660	-0.001182
H	-5.047443	2.200079	0.036597
S	-0.941481	-1.662897	-0.072609
H	4.132482	0.869603	0.171572
N	5.318710	1.456368	0.027473
H	5.713626	0.810814	-0.661770
H	5.014446	2.256046	-0.534554

14d

E (PCM(methanol)-[M06-2X/def2-TZVP])= -1113.47198784

C	-2.126877	-1.735122	1.181255
C	-2.714336	-0.211473	-0.969377
C	-3.564736	-1.442153	0.646773
C	-1.646662	-2.011252	-0.275390
C	-3.079913	-1.716281	-0.809946
C	-1.266687	-0.498722	-0.432990
C	-3.196817	0.066283	0.486692
C	-1.766250	-0.224895	1.027840
H	-1.887808	-2.350681	2.043664
H	-2.937793	0.401733	-1.838712
H	-1.010917	-2.836702	-0.584082
H	-3.608120	-2.313624	-1.546491
H	-3.818759	0.903823	0.788465
H	-1.237416	0.381159	1.759018
C	0.462175	1.697885	-0.454084
C	1.699839	2.154478	-0.004076
C	-0.647124	2.538531	-0.332507
C	1.824885	3.419865	0.557489
H	2.572348	1.516231	-0.081491
C	-0.522751	3.800098	0.231740
H	-1.621128	2.204860	-0.675219

C	0.715862	4.245798	0.678057
H	2.792684	3.757660	0.906395
H	-1.394815	4.435281	0.323917
H	0.814811	5.229552	1.118595
P	0.249230	0.066357	-1.271541
H	-4.483610	-1.820889	1.083428
C	1.545095	-0.929691	-0.441771
C	1.409762	-1.434929	0.853225
C	2.712985	-1.207150	-1.150523
C	2.418730	-2.199098	1.421458
C	3.732153	-1.958865	-0.577224
C	3.583952	-2.458925	0.708641
H	0.513323	-1.230998	1.426600
H	2.824760	-0.833477	-2.162121
H	2.297947	-2.590971	2.423450
H	4.635129	-2.161578	-1.138830
H	4.371214	-3.053023	1.154848

14d-TS

E (PCM(methanol)-[M06-2X/def2-TZVP])= -1169.33306707. Neg-Freq=-1660.3

C	2.489093	0.715949	0.584473
C	1.902451	-1.364623	-1.054723
C	3.444829	-0.434691	0.171909
C	2.047772	0.837021	-0.904804
C	3.019057	-0.309680	-1.317155
C	0.917361	-0.215722	-0.646963
C	2.341078	-1.493275	0.437683
C	1.372599	-0.339804	0.847498
H	2.730533	1.551127	1.234432
H	1.654535	-2.194804	-1.710261
H	1.906887	1.770645	-1.442866
H	3.681666	-0.295084	-2.176267
H	2.466077	-2.429156	0.974031
H	0.708094	-0.352926	1.706765
C	-1.732500	-1.226421	-0.467113
C	-3.125931	-1.131535	-0.513629
C	-1.159301	-2.324932	0.171051
C	-3.923993	-2.097467	0.078555
H	-3.590472	-0.287701	-1.012663
C	-1.959594	-3.301290	0.755249
H	-0.082728	-2.432931	0.220028
C	-3.341322	-3.188656	0.714896
H	-5.001800	-2.002613	0.040293
H	-1.497351	-4.147853	1.247001
H	-3.963271	-3.946198	1.174074
P	-0.746797	0.053595	-1.327504

H	4.636549	-0.566450	0.540326
N	5.850427	-0.635463	1.115025
H	5.949962	0.356480	1.347791
H	5.559173	-1.063152	1.998449
C	-1.172852	1.561047	-0.365392
C	-1.581009	1.529759	0.968991
C	-1.017434	2.796810	-0.993492
C	-1.827756	2.709220	1.657682
C	-1.254448	3.978720	-0.302017
C	-1.662904	3.935564	1.023788
H	-1.713367	0.577636	1.469417
H	-0.712153	2.833722	-2.033440
H	-2.147992	2.672196	2.691328
H	-1.128956	4.930830	-0.801681
H	-1.857596	4.854588	1.561689

3.4.2 HOMO-LUMO Levels of selected compounds

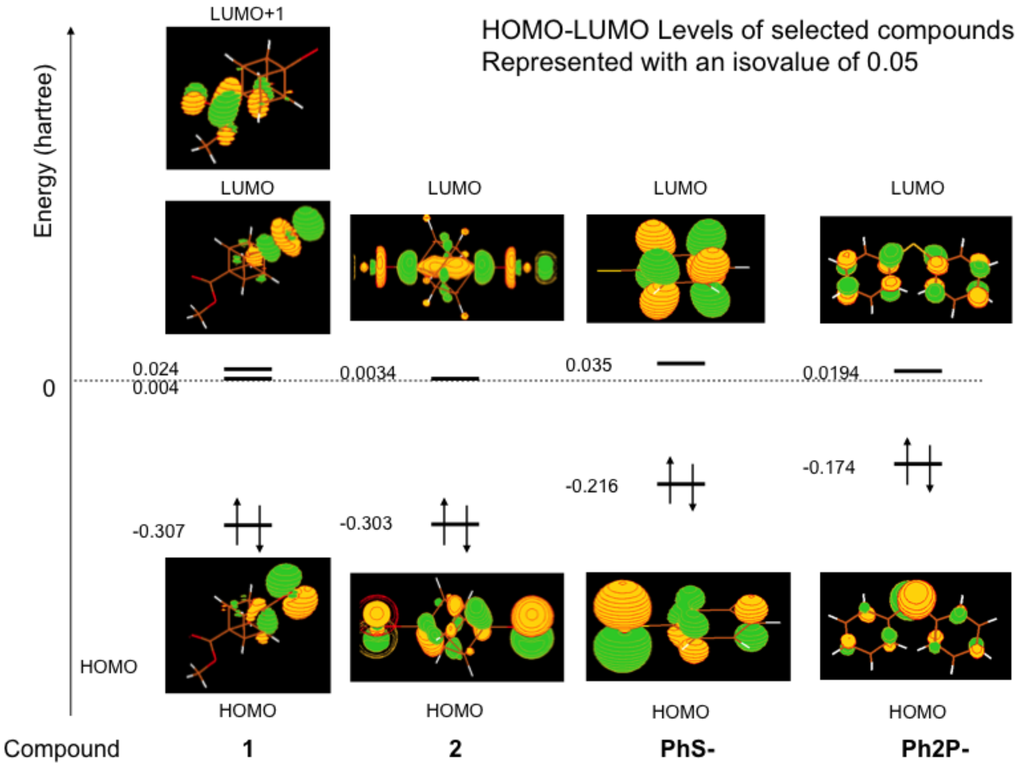

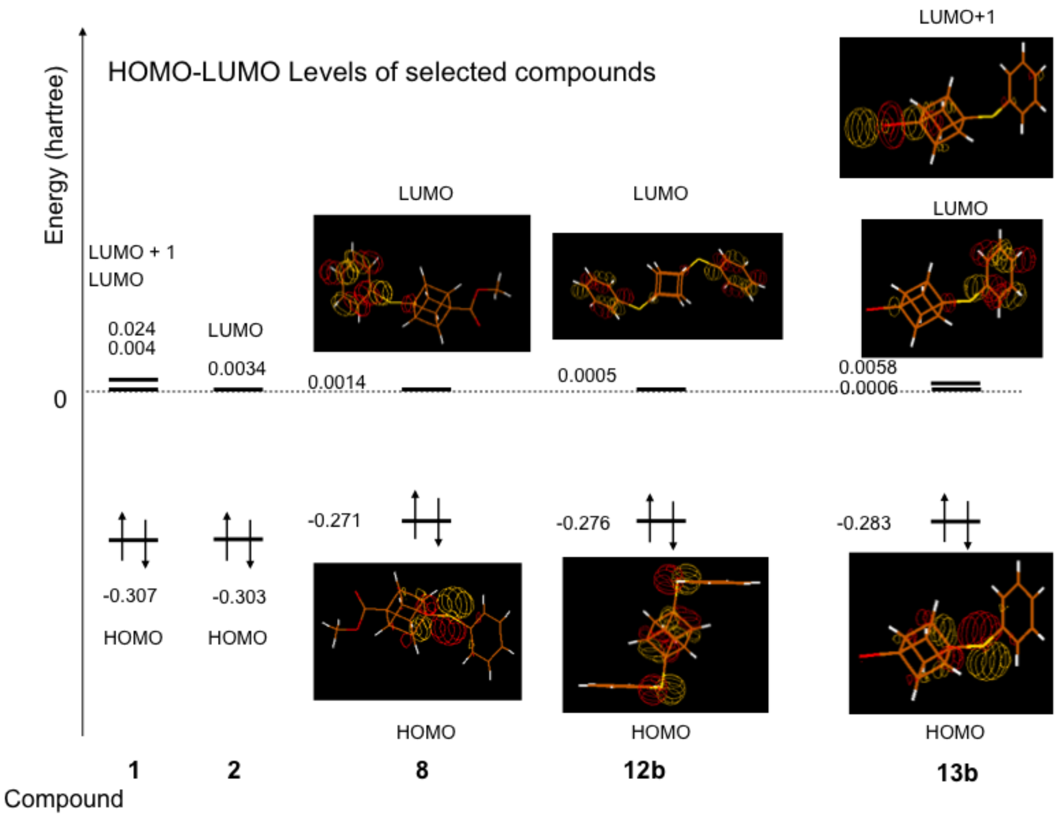

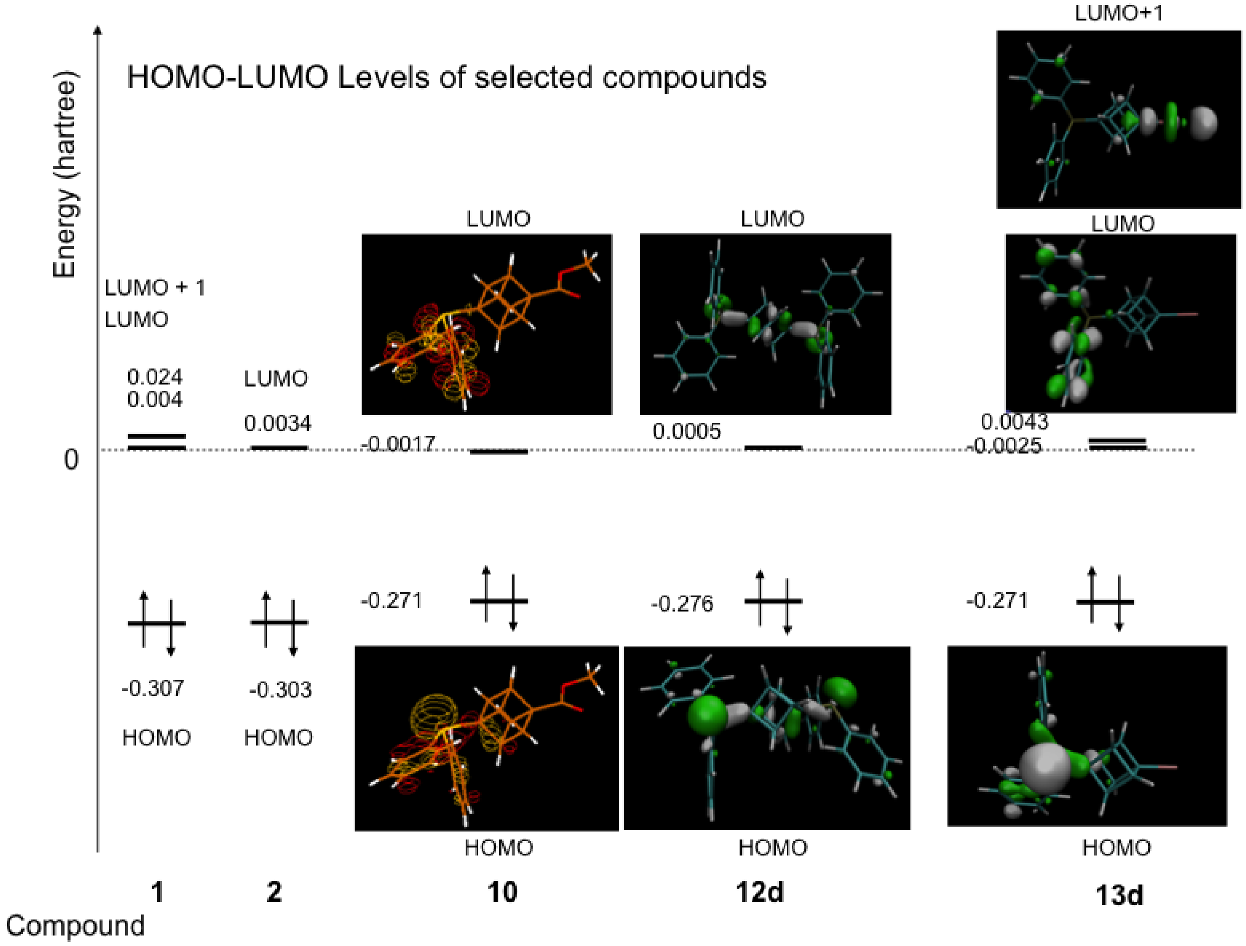
HOMO-LUMO Levels of selected compounds
Energy (hartree)
LUMO+1
LUMO
LUMO
LUMO
LUMO + 1
LUMO
0.024
0.004
LUMO
0.0034
-0.0017
0.0005
0.0043
-0.0025
0
-0.271
-0.276
-0.271
-0.307
-0.303
HOMO
HOMO
HOMO
HOMO
HOMO
1
2
10
12d
13d
Compound

3.4.3 Estimation of the Activation barrier by Marcus and Savéant's models

There can be different scenarios for dissociative electron transfer (DET) from the nucleophiles to the cubane derivatives. If ET and cleavage of the C-I bond in the iodocubane occur in different steps and involve the formation of the cubane-halide radical anion, the outer-sphere ET model is applicable and the activation barrier may be estimated from the outer-sphere Marcus-Hush model. Otherwise, when the ET and aryl halide bond cleavage are concerted (concerted dissociative ET), Savéant's model is used.

The reorganization energy λ_0 according to Marcus equation can be calculated with:

$$\lambda_0 = (332\ kcal/mol)\left(\frac{1}{2a_1}+\frac{1}{2a_2}-\frac{1}{R}\right)\left(\frac{1}{\varepsilon_{op}}-\frac{1}{\varepsilon}\right) \quad (1)$$

where a_1 and a_2 are the radii of the reactant molecules and $R = a_1 + a_2$. ε_{op} is the optical dielectric constant and ε is the static dielectric constant. The inner reorganization energy was estimated to be $\lambda_i \approx 0$.

The Marcus equation is:

$$\Delta G^{\ddagger}_{ET} = \Delta G^{\ddagger}_{0}\left(1+\frac{\Delta G_r}{4\Delta G^{\ddagger}_{0}}\right)^2 \quad (2)$$

where ΔG_r is the reaction energy and $\Delta G_0^{\ddagger}$ is the intrinsic barrier. Within the Marcus Model the equation 3 was used, while in the Savéant Model equation 4 was used:

$$\Delta G^{\ddagger}_{0} = \frac{\lambda}{4} = \frac{\lambda_i+\lambda_0}{4} \quad (3)$$

$$\Delta G^{\ddagger}_{0} = \frac{\lambda}{4} = \frac{\lambda_i+\lambda_0+BDFE}{4} \quad (4)$$

where BDFE stand for bond dissociation free energy of diiodocubane (60.0 kcal/mol).

Single Electron Transfer from $8^{\bullet-}$ to 1 by Marcus-Hush Model

Solvent	ΔG (kcal/mol)	ε_{op}	ε	λ_0 (kcal/mol)	λ_i(kcal/mol)	λ(kcal/mol)	$\Delta G_{ET}^{\ddagger}$
Methanol	+3.1	32.6	1.76	17.6	0.0	17.6	+6.1
$a_0(7^{-\bullet})$	5.38 Å						
a_0(1a)	4.79 Å						

Single Electron Transfer from $10^{\bullet-}$ to 1 by Marcus-Hush Model

Solvent	ΔG (kcal/mol)	ε_{op}	ε	λ_0 (kcal/mol)	λ_i(kcal/mol)	λ(kcal/mol)	ΔG S75
Methanol	+6.1	32.6	1.76	17.6	0.0	17.6	+8.0
$a_0(9^{-\bullet})$	5.51 Å						
a_0(1a)	4.79 Å						

Single Electron Transfer from 13b$^{\bullet -}$ to 2 by Savéant Model

Solvent	ΔG (kcal/mol)	ε_{op}	ε	λ_0 (kcal/mol)	λ_i(kcal/mol)	λ(kcal/mol)	$\Delta G_{ET}^{\ddagger}$
Methanol	-44.5	32.6	1.76	18.6	0.0	79.0	+3.8
$a_0(12b^{-\bullet})$	5.05 Å						
a_0(1b)	4.55 Å						

Single Electron Transfer from 13d$^{\bullet -}$ to 2 by Savéant Model

Solvent	ΔG (kcal/mol)	ε_{op}	ε	λ_0 (kcal/mol)	λ_i(kcal/mol)	λ(kcal/mol)	$\Delta G_{ET}^{\ddagger}$
Methanol	-40.2	32.6	1.76	18.2	0.0	78.6	+4.7
$a_0(12d^{-\bullet})$	5.36 Å						
a_0(1b)	4.55 Å						

3.3.3 DFT functional and post-HF methods for Activation Gibb Energies ($\Delta G^{\ddagger}$, in kcal/mol), Activation Enthalpy ($\Delta H^{\ddagger}$, in kcal/mol)

Table 1. Computed Activation Gibb Energies ($\Delta G^{\ddagger}$, in kcal/mol), Activation Enthalpy ($\Delta H^{\ddagger}$, in kcal/mol) in the solvent and Kinetic parameters computed (log k) for the reaction between radical **15 and $^{-}$SPh**. The numbers in parenthesis are in the gas phase.

Functional	$\Delta H^{\ddagger}$	$\Delta G^{\ddagger}$	Log k
M06-2X/def2-TZVP (results presented in the manuscript)	8.7 (-5.2)	17.6 (4.2)	1.6
M06-HF/def2-TZVP	6.3(-8.3)	15.9 (1.6)	3.8
M06-L/def2-TZVP	9.2 (-4.7)	17.9 (5.0)	1.2
M06/def2-TZVP	7.2 (-5.8)	18.4 (5.5)	3.0
B3LYP/def2-TZVP	10.9 (-2.9)	19.2 (6.5)	-0.3
B3LYP+D3(BJ)/def2-TZVP	5.8 (-8.2)	14.3 (2.2)	4.3
B3PW91+D3(BJ)/def2-TZVP	4.1 (-9.0)	12.9 (1.4)	5.9
BH&HLYP/def2-TZVP	15.6 (2.0)	27.1 (12.4)	-4.6
PBE0/def2-TZVP	8.2 (-4.7)	16.3 (5.1)	2.1
PBE0+D3(BJ)/def2-TZVP	4.9 (-8.0)	14.0 (1.8)	5.1
TSPPh+D3(BJ)/def2-TZVP	3.4 (-9.5)	12.0 (1.4)	6.4
wB97/def2-TZVP	12.0 (-1.8)	22.6 (8.7)	-1.4
wB97X/def2-TZVP	11.6(-2.1)	22.8 (7.8)	-1.0

wB97XD/def2-TZVP	9.5 (-4.4)	21.3 (4.4)	1.0
DPLNO-CCSD(T)/cc-pVTZ//M06-2X/def2-TZVP	7.2 (-5.8)	16.0 (3.0)	3.0

Kinetic parameters computed (log k) were calculated from the Eyring-Polanyi equation (see below). This equation comes actually from the extension of Arrhenius equation but employing the Transition State Theory. The logarithmic expression is

$$\log k = \log \kappa \frac{k_B T}{h} - \frac{\Delta G^{\mp}}{2.303RT}$$

$$\log k = \log \kappa \frac{k_B T}{h} + \frac{\Delta S^{\mp}}{2.303R} - \frac{\Delta H^{\mp}}{2.303RT}$$

where κ is the transmission coefficient (normally taken as the unit), k_B is the Boltzmann's constant, *h* is Planck's constant, $\Delta S^{\ddagger}$ activation entropy and $\Delta H^{\ddagger}$ activation enthalpy.
